# Ribosome-mediated biosynthesis of pyridazinone oligomers in vitro

**DOI:** 10.1038/s41467-022-33701-2

**Published:** 2022-10-24

**Authors:** Joongoo Lee, Jaime N. Coronado, Namjin Cho, Jongdoo Lim, Brandon M. Hosford, Sangwon Seo, Do Soon Kim, Camila Kofman, Jeffrey S. Moore, Andrew D. Ellington, Eric V. Anslyn, Michael C. Jewett

**Affiliations:** 1 Department of Chemical and Biological Engineering, Northwestern University, Evanston, IL 60208 USA; 2 Department of Chemical Engineering, Pohang University of Science and Technology (POSTECH), Pohang, 37673 Republic of Korea; 3 Department of Chemistry, University of Texas at Austin, Austin, TX 78712 USA; 4 Department of Chemistry, Korea Advanced Institute of Science and Technology (KAIST), Daejeon, 34141 Republic of Korea; 5 Center for Catalytic Hydrocarbon Functionalizations, Institute for Basic Science (IBS), Daejeon, 34141 Republic of Korea; 6 Department of Chemistry, University of Illinois at Urbana-Champaign, Urbana, IL 61801 USA; 7 Beckman Institute for Advanced Science and Technology, University of Illinois at Urbana-Champaign, Urbana, IL 61801 USA; 8 Department of Chemistry and Biochemistry, Institute for Cellular and Molecular Biology, University of Texas at Austin, Austin, TX 78712 USA; 9 Interdisplinary Biological Sciences Graduate Program, Evanston, IL 60208 USA; 10 Chemistry of Life Processes Institute, Evanston, IL 60208 USA; 11 Robert H. Lurie Comprehensive Cancer Center, Evanston, IL 60208 USA; 12 Simpson Querrey Institute, Evanston, IL 60208 USA; 13 Center for Synthetic Biology, Northwestern University and Biological Engineering, 2145 Sheridan Road, Evanston, IL 60208 USA

**Keywords:** Catalytic RNA, Synthetic biology

## Abstract

The ribosome is a macromolecular machine that catalyzes the sequence-defined polymerization of L-α-amino acids into polypeptides. The catalysis of peptide bond formation between amino acid substrates is based on entropy trapping, wherein the adjacency of transfer RNA (tRNA)-coupled acyl bonds in the P-site and the α-amino groups in the A-site aligns the substrates for coupling. The plasticity of this catalytic mechanism has been observed in both remnants of the evolution of the genetic code and modern efforts to reprogram the genetic code (e.g., ribosomal incorporation of non-canonical amino acids, ribosomal ester formation). However, the limits of ribosome-mediated polymerization are underexplored. Here, rather than peptide bonds, we demonstrate ribosome-mediated polymerization of pyridazinone bonds via a cyclocondensation reaction between activated γ-keto and α-hydrazino ester monomers. In addition, we demonstrate the ribosome-catalyzed synthesis of peptide-hybrid oligomers composed of multiple sequence-defined alternating pyridazinone linkages. Our results highlight the plasticity of the ribosome’s ancient bond-formation mechanism, expand the range of non-canonical polymeric backbones that can be synthesized by the ribosome, and open the door to new applications in synthetic biology.

## Abstract (web summary)

Ribosomes have evolved to polymerize L-α-amino acids into proteins comprising a peptide backbone. Here, a pyridazinone backbone is formed using ribosomes in vitro, producing a variety of sequence-defined alternating block-copolymers.

## Introduction

Ribosomes have evolved to prefer l-α-amino acid substrates and to polymerize these substrates via peptide (i.e., amide) bond formation^1–3^. Peptide bond formation is catalyzed by the peptidyl transferase center (PTC) and is based on entropy trapping^1,4^, wherein a nucleophilic α-amino group of the A-site aminoacyl-transfer RNA (tRNA) consecutively attacks an electrophilic ester linkage of the P-site tRNA carrying the growing polymeric chain (i.e., (poly)peptide). Despite a preference for the 20 canonical amino acids, ribosome-mediated polymerization has shown plasticity^5^. For example, genetic code reprogramming technologies have been used to site-specifically incorporate hundreds of distinct non-canonical amino acids (ncAAs) into peptides and proteins to expand the range of genetically encoded chemistry^6–10^. These ncAAs have included α-, β-, γ-, δ-, ε-, ζ-, cyclic, D-, and N-alkylated amino acids^11–19^, as well as alternative monomers (e.g., non-amino carboxylic acids, hydroxy acids, aminoxy acids, hydrazino acids, and thioacids)^20–24^.

While genetic code expansion has extended the limits of monomers amenable to ribosome-mediated polymerization, their polymeric structures remain confined to a much smaller chemical space composed of amide linkages, (-CONH-)^14–16,25,26^, or close analogs such as esters (-COO-)^22,27^, thioesters (-COS-)^28^, or thioamides (-CSNH-)^24^. Yet, the model that the ribosome employs entropic catalysis to accelerate peptide bond formation by positioning substrates, reorganizing water in the PTC, and stabilizing reaction intermediates suggests that a broader range of alternatives may be possible.

**Fig. 1:**
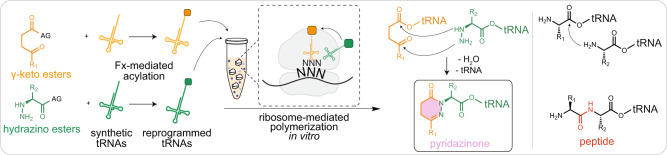
Ribosome-catalyzed formation of pyridazinone bonds in vitro. Genetic code reprogramming using the flexizyme system enables the acylation of non-canonical substrates with tRNA. Upon flexizyme-mediated tRNA acylation of keto (orange) and hydrazino (green) activated esters, the programmed keto-tRNA (orange) and hydrazinyl-tRNA (green) were added to an in vitro transcription and translation platform using purified components and allowed to decode two consecutive codons programmed on an mRNA strand. The translation mixture produced a pyridazinone bond (pink). For comparison, the typical peptide bond (red) is shown on the right. Fx flexizyme, AG activating group, CME cyanomethyl ester, DNB dinitrobenzylester, and ABT amino-derivatized benzylthio ester.

In this work, we set out to identify alternative polymer backbone linkages suitable for ribosome-mediated polymerization. Given the high structural dependence of peptide bond formation in the PTC, we designed monomers that closely resembled the structure of the proteinogenic amino acids, such that the reactive components would be oriented correctly for bond formation to occur. As a donor, we chose to use a monomer that possesses a nucleophilic hydrazine group in place of the α-amine. As an acceptor, we selected γ-keto esters. We surmised that coupling and rearrangement of activated hydrazines and γ-keto esters by the ribosome could produce 6-membered heterocyclic rings called pyridazinones (Fig. 1). Several features supported our design choice. First, the heterocyclic literature is replete with reactions between hydrazines and keto esters to form pyrazolones, pyridazinones, and other heterocycles^29–31^, as these structural motifs are often found as key pharmacophores^32,33^. Second, several recent efforts have shown the ability to incorporate α-hydrazino monomers into peptides by the ribosome in vitro^26,34^. Third, cyclocondensation of hydrazine and a keto ester begins with hydrazone formation followed by cyclization, which is an amide forming step, a specialty of the ribosome.

## Results

### Design of non-canonical monomers for an alternative polymer backbone

Ribosome-catalyzed formation of pyridazinone bonds required the activated γ-keto and hydrazino ester monomers and the subsequent charging of these monomers to tRNAs. Because these monomers do not have associated aminoacyl-tRNA synthetases necessary for tRNA acylation, we decided to charge tRNAs with the flexizyme (Fx) system. Flexizymes are aminoacyl-tRNA synthetase-like ribozymes that catalyze the acylation of tRNA with diverse substrates^35,36^. Because Fx recognizes the 3′-CCA sequence of tRNA and either the benzyl group of an acyl substrate or the leaving group of the activated ester substrate^37^, virtually any monomer can be acylated so long as it possesses an appropriate activating group (e.g., cyanomethyl ester (CME), dinitrobenzylester (DNB), or (2-aminoethyl)amidocarboxybenzyl thioester (ABT)). For acylation of phenylalanine or tyrosine, for example, CME (non-aromatic leaving group) is preferably used as an ester leaving the group because the substrate contains a benzyl group on the sidechain^38^. In contrast, for acylation of alanine, aspartic acid, or other aliphatic residues not bearing an aromatic group, DNB or ABT is used to provide the aromaticity and thereby gain Fx recognition^20,38^. Fx has been used extensively to expand the limits of a reprogrammed genetic code^20,39^.

**Fig. 2:**
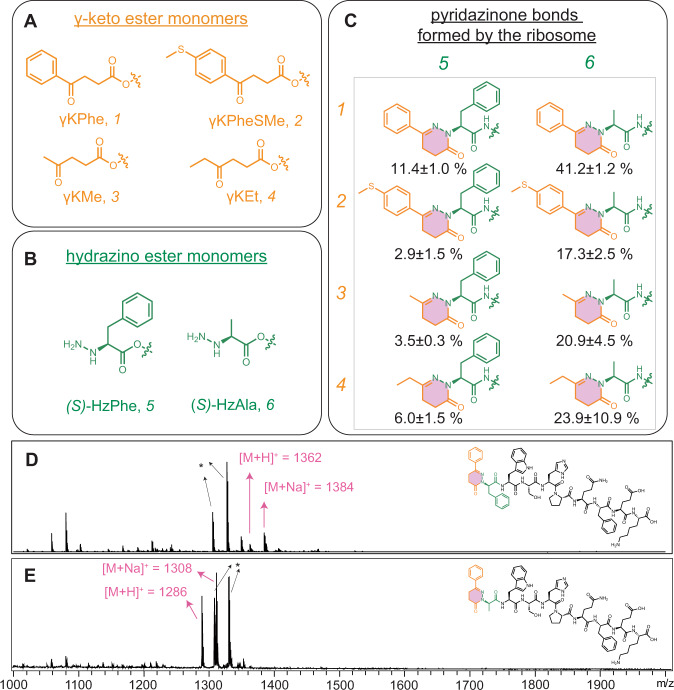
Design of γ-keto and hydrazino esters and ribosome-mediated synthesis of pyridazinone bonds. **A** Four γ-keto (orange) and **B** two hydrazino (green) esters were synthesized with an activated leaving group (CME, DNB, and ABT). DNB or ABT were used for the substrates that do not contain an aromatic moiety and the ABT-activated substances were only synthesized when the DNB substrates were found to be water-insoluble (Supplementary Information; *1*-CME, *2*-CME, *3*-DNB, *3*-ABT, *4*-DNB; *5*-CME, *6*-DNB, *6*-ABT). The substrates were charged to tRNA by the appropriate Fx and introduced to an in vitro translation reaction containing wild-type ribosomes. **C** In vitro translation reactions were carried out with pairs of γ-keto ester substrates (**A**) and hydrazino ester substrates (**B**). Ribosome-catalyzed synthesis of eight different pyridazinone rings was observed. The relative percent yield of the target oligomer of all species was determined by the peak area corresponding to the theoretical mass/the sum of areas of the whole peaks shown in the mass spectrum, as shown in matrix-assisted laser desorption/ionization-time-of-flight (MALDI-TOF) mass spectra (Supplementary Information). Percent yield is based on *n* = 3 reactions. **D**, **E** MALDI-TOF mass spectra of oligomers polymerized by the ribosome in vitro with a pyridazinone bond formed between *1* and *5*, and *1* and *6*, respectively. The calculated masses of the products in **D** are [M + H]^+^ = 1362, [M + Na]^+^ = 1384 and in **E** are [M + H]^+^ = 1286, [M + Na]^+^ = 1308. See SI for MALDI-TOF mass spectra of the other pyridazinone bonds represented in **C**. The non-target products at masses 1058 and 1080 (#) and 1305 and 1327 (*) are a reporter strep-tag alone (TrpSerHisProGlnPheGluLys) and the peptide containing a misincorporated Ser^60,61^ at the Thr (ACC) codon (*1*(Ser)TrpSerHisProGlnPheGluLys, see Supplementary Fig. 2 for details). Spectra in **D** and **E** are representative of *n* = 3 independent experiments.

We first designed a series of γ-keto and hydrazino monomers with different Fx-leaving groups to assess tRNA acylation (Fig. 2). The γ-keto ester substrates were prepared by esterification of γ-keto carboxylate with an activating group (AG)^20^. The hydrazino substrates were synthesized in three steps: (i) N-amination of Phe or Ala with an N-Boc-protected electrophilic amino source^40,41^, (ii) esterification of carboxylate with an AG^42^, and (iii) Boc deprotection from the β-nitrogen^16^. To determine the acylation efficiency, we used a small tRNA mimic microhelix (mihx, 22nt) as an acyl acceptor^36^. Mihx is conventionally used as a tRNA analog for analyzing acylation efficiency. Fx-mediated reactions were carried out in six different conditions [two different pHs (7.5 and 8.8) with three different flexizymes (eFx, dFx, and aFx)] for each synthetic γ-keto and α-hydrazino ester to optimize yields. Yields of Fx-catalyzed acylation were determined by densitometric analysis of RNA bands on an acidic polyacrylamide gel (pH 5.2, 3 mM NaOAc), and ranged from 21–82% (Supplementary Fig. 1).

### Ribosome-mediated polymerization of pyridazinone bonds

Using the conditions optimized from our Fx-mihx experiments that assessed acylation efficiency (Supplementary Fig. 1), we produced acyl-tRNAs bearing the four γ-keto ester and two hydrazine monomers (Fig. 2A, B). After the Fx-mediated tRNA acylation, unreacted monomers were separated from the tRNAs using ethanol precipitation. The resulting tRNA fraction that includes the tRNA-substrates was supplemented as a mixture into an *Escherichia coli*-based in vitro transcription and translation reaction containing a minimal set of components required for translation (PURExpress^TM^)^43^. As a reporter oligomer, we designed a T7 promoter-controlled plasmid (pJL1_StrepII) encoding a streptavidin tag (XY + WSHPQFEK), where X and Y indicate the positions to which an Fx-charged γ-keto ester (*1*) and hydrazino substrate (*5*) are incorporated, respectively. The in vitro transcription and translation reactions were carried out in the presence of all *E. coli* (>46) endogenous tRNAs, but only eight amino acids encoding the polypeptide streptavidin tag. For site-specific incorporation of *1* and *5* into the N-terminal X and Y residue, we first charged substrates *1* and *5* onto tRNA^fMet^(CAU) and tRNA^Pro1E2^(GGU), respectively^44,45^. We selected the AUG and ACC codons on mRNA because the AUG (CAU anticodon) codon is the canonical initiation codon for N-terminal incorporation and Thr (ACC) is not used to express the streptavidin tag. This prevented corresponding endogenous tRNAs in the PURExpress^TM^ reaction from being aminoacylated, and from competing in the translation reaction. For the incorporation of *5*, tRNA^Pro1E2^(GGU)^16,44^ was selected because it has an engineered D-arm and T-stem for interacting with translation elongation factors to promote the incorporation of a charged substrate^16,46,47^.

Following in vitro translation in PURExpress^TM^ reactions for 2 h, the synthesized oligomers were purified using Strep-Tactin-coated magnetic beads, denatured with SDS, and characterized by matrix-assisted laser desorption/ionization-time-of-flight (MALDI-TOF) mass spectrometry. We observed a peak corresponding to the mass of an oligomer bearing a pyridazinone bond between *1* and *5* incorporated consecutively into the oligomer. The percent yield of pyridazinone formation of ~10% was calculated based on the relative peak area of the peptides shown in the mass spectrum from 1000 to 2000 Da (Fig. 2C, D), assuming the calibration factor to be 1.0 (Supplementary Fig. 3). To our knowledge, this is the first example of intramolecular cyclic structure formation catalyzed by the ribosome in vitro.

We next tried to enhance the yield of oligomers containing pyridazinone. First, we incubated the PURExpress^TM^ reaction mixture for a longer time (24 h). Unfortunately, extending the reaction time did not increase the production of pyridazinone-peptide product. Second, we changed the hydrazine monomer. Previous studies have shown that bulky, non-canonical ribosomal substrates can have reduced incorporation efficiencies and product formation^15^. Thus, instead of cyanomethyl amino-l-phenylalaninate (*S*)-HzPhe *5*, we used the less bulky 3,5-dinitrobenzyl amino-L-alaninate (*S*)-HzAla *6*, which contains only a methyl group on the sidechain, to see if pyridazinone oligomer formation could be increased. As above, we carried out Fx-mediated tRNA acylation, isolated tRNA complexes, supplemented them into the PURExpress^TM^ reaction, and characterized the resulting product by MALDI-TOF. In the MALDI spectrum (Fig. 2E), we observed a peak corresponding to the mass of the oligomer containing pyridazinone in a yield (relative peak area) of ~48% of the total product. This ~4-fold increase in yield indicates the natural translation system can incorporate less bulky (*S*)-HzAla *6* at higher efficiencies compared to (*S*)-HzPhe *5*.

To further explore the ribosome-mediated pyridazinone formation reaction, we next tested additional γ-keto acids with both hydrazino esters (*5* and *6*). Specifically, we used cyanomethyl 4-(4-(methylthio)phenyl)−4-oxobutanoate (*2*, γKPheSMe-CME), 3,5-dinitrobenzyl 4-oxopentanoate (*3*, γKMe-DNB), and 3,5-dinitrobenzyl 4-oxohexanoate (*4*, γKEt-DNB). We carried out the Fx-mediated acylation reaction onto a tRNA^fMet^(CUA) and tRNA^Pro1E2^(GGU). Subsequently, we added the two tRNAs charged with a γ-keto and hydrazino ester in all the six possible combinations (i.e., *2*:*5*, *3*:*5*, *4*:*5*, *2*:*6*, *3*:*6*, and *4*:*6*) to PURExpress^TM^ reactions. The MALDI-TOF spectra (Supplementary Figs 4–6) for each of the purified peptides show a peak corresponding to the theoretical mass of the oligomer containing a different pyridazinone. To confirm that the pyridazinone group is only produced when tRNA^fMet^(CAU):*1* and tRNA^Pro1E2^(GGU):*5* are supplemented into the reaction mixture together, we carried out three control experiments under the same condition. As a positive control, we ran the reaction in the presence of the 20 natural amino acids and found peaks ([M + H]^+^ = 1318, [M + Na]^+^ = 1340, and [M-H + 2Na]^+^ = 1362) corresponding to the theoretical mass of fMetThrTrpSerHisProGlnPheGluLys (Supplementary Fig. 4A). When we omitted either tRNA^fMet^(CAU):*1* or tRNA^Pro1E2^(GGU):*5*, and supplemented a different set of nine amino acids ([TWSHPQFEK] or [MWSHPQFEK]) into the reaction, we observed peaks of ([M + H]^+^ = 1319, [M + Na]^+^ = 1341, and [M-H + 2Na]^+^ = 1363) or ([M + H]^+^ = 1379, [M + Na]^+^ = 1401, and [M-H + 2Na]^+^ = 1423) corresponding to the mass of linear products, (*1*)ThrTrpSerHisProGlnPheGluLys or fMet(*5*)TrpSerHisProGlnPheGluLys, respectively (Supplementary Fig. 4B, C), suggesting that the consecutive incorporations of the two substrates are required for the pyridazinone bond formation.

### The ribosome is required for the pyridazinone bond formation in vitro

**Fig. 3:**
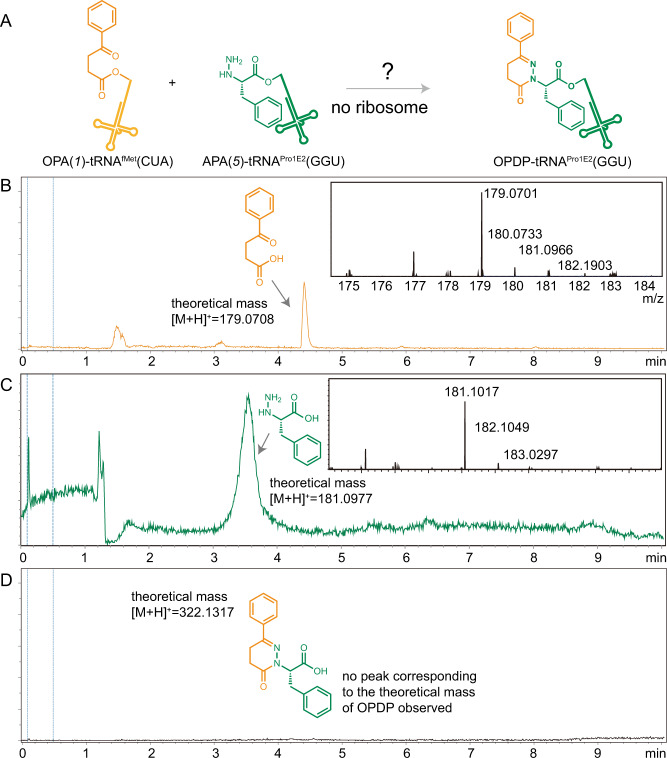
The ribosome is required for pyridazinone formation under in vitro translation conditions. **A** The in vitro polymerization reaction was conducted using the same conditions that produced an N-terminal pyridazinone bond in an oligomer, but without the presence of ribosomes. In the deconvoluted mass spectra, the compounds having a molar mass of 179.0701 and 181.1017 were observed as a single peak at 4.4 and 3.6 min, which corresponds to 4-oxo-4-phenylbutanoic acid (orange in **B**) aminophenylalanine (green in **C**) hydrolyzed from tRNA^fMet^(CUA) and tRNA^Pro1E2^(GGU), respectively. **D** No species corresponding to the theoretical mass of OPDP (322.1317) were observed in the reaction mixture. The extracted ion chromatograms were obtained based on theoretical average masses (Supplementary Information). Spectra are representative of *n* = 3 independent experiments.

Our data showed the ability of ribosome-mediated cyclocondensation to form eight different pyridazinone derivatives (Fig. 2C). However, we wondered about the possibility that pyridazinone bonds could be created in the in vitro reaction between the hydrazino and keto ester monomers without the ribosome. We, therefore, performed a negative control reaction to assess possible pyridazinone formation with a PURExpress^TM^ reaction under the same conditions as above with aminocyl-tRNA monomers *1* and *5* but without ribosomes (Fig. 3A). Following PURExpress^TM^ reactions, we analyzed the crude reaction mixture by liquid chromatography-time-of-flight (LC-TOF) mass spectrometry. We observed a single peak corresponding to the theoretical masses of the monomers 4-oxo-4-phenylbutanoic acid (OPA, Fig. 3B) and aminophenylalanine (APA, Fig. 3C) from the extracted ion chromatogram after deconvoluting it with the theoretical mass. In contrast, no peak corresponding to the theoretical mass of the resulting pyridazinone (2-(6-oxo-3-phenyl-5,6-dihydropyridazin-1(4H)-yl)−3-phenylpropanoic acid, OPDP) was found (Fig. 3D). This result indicates that pyridazinone formation does not occur under our in vitro reaction conditions in the absence of the ribosome.

We next investigated the regioselectivity of the pyridazinone linkage. Two possible regioisomers may be produced in the peptidyl transferase center of the ribosome, a 1,6- and/or 2,6-substituted pyridazinone (Supplementary Fig. 7). To better understand the regioselectivity of pyridazinone formation, we carried out chemical reactions between the γ-keto cyanomethyl ester (cyanomethyl 4-oxo-4-phenylbutanoate (COPB, analog to *1*)) and the hydrazino ester (ethyl hydrazinoacetate hydrochloride (EHAH, analog to *5* and *6*)). We were curious to see if the regioselectivity of the resulting pyridazinone bonds produced in solution, by which we may infer the structure of pyridazinone produced in the ribosome. We carried out the reaction of COPB and EHAH in three different concentrations (40 µM, 4 mM, and 40 mM) in EtOH/H_2_O (3/2:v/v) at 37 °C and analyzed the resulting product by liquid chromatography-mass spectrometry (LC-MS) at two different reaction times (2 and 24 h). The pyridazinone product was not found by LC-MS at the concentrations of 40 μM and 4 mM, while the peaks for the carboxylic acid (hydrolyzed by water), the starting cyanomethyl ester, and the ethyl ester (esterified by ethanol) were observed at 3.4, 4.9, and 5.6 min, respectively (Supplementary Fig. 8A, B). Importantly, 40 μM is the concentration of tRNA^fMet^(CAU):*1* and tRNA^Pro1E2^(GGU):*5* supplemented into the PURExpress^TM^ reactions that catalyzed pyridazinone formation, further confirming that the ribosome is necessary for production of the pyridazinone-peptide hybrids. In the 40 mM reaction, the pyridazinone product was formed and observed at 5.5 min (Supplementary Fig. 8C, yield: 2% (2 h) and 6% (24 h)) by LC-MS. Analysis by ^1^H NMR spectroscopy (Supplementary Fig. 8D) showed that the isolated product was exclusively the 2,6-substituted pyridazinone. Admittedly, the reactivity of the α- and β-nitrogen in the ribosome might be different, leading to the formation of an amide and hydrazone bond linked to either the α- and β-nitrogen, i.e., 1,6-substituted pyridazinone formation is another possibility (Supplementary Fig. 7C, D). Mixtures of 1,6- and 2,6-substitution patterns could also be formed. Unfortunately, due to the low yield of the peptide bearing a pyridazinone (~30–60 ng as determined using an internal peptide standard, see Supplementary Fig. 9 for details), we were unable to analyze the peptide product through NMR. Future efforts to elucidate the regioselectivity of ribosomally produced peptides will be informative. Further investigations involving additional synthetic substrates that selectively form an amide with α- or β- nitrogen atom might also be a possible strategy to elucidate the structure of the ribosome-generated pyridazinone more clearly.

After confirming the ribosome is a necessary catalyst for pyridazinone ring formation in our PURExpress^TM^ reaction conditions, we explored the impact of supplementing additional translation factors. Previously, supplementing in vitro transcription and translation reactions with engineered ribosomes^48,49^ and Elongation Factor P (EF-P) have increased yields of polymers with poorly compatible substrates^14–16,20^. For example, an engineered ribosome, termed 040329, enabled the incorporation of dipeptides by the ribosome, which was later shown to facilitate the incorporation of backbone extended monomers^14^. In addition, EF-P is a bacterial translation factor that accelerates peptide bond formation between consecutive prolines and has been shown to help alleviate ribosome stalling as a result of D- and β- amino acid substrates^16,46^. To test if supplementation benefitted the synthesis of pyridazinone-peptide oligomers, we prepared purified mutant ribosomes as a mixture of wild-type and 040329 ribosomes and EF-P (Supplementary Information), as reported previously^14^. We carried out PURExpress^TM^ reactions with substrates *1* and *5*, and purified and analyzed the products by MALDI-TOF mass spectrometry. In the resulting MALDI spectra, we observed the peak corresponding to the theoretical mass of a target oligomer containing a pyridazinone bond increases ~3% in the presence of engineered ribosomes (Supplementary Fig. 10). Production of a pyridazinone bond was inhibited ~8% when EF-P was supplemented with just wild-type ribosomes or combined wild-type or engineered ribosomes.

### Sequence-defined polymerization of pyridazinone bonds by the ribosome

**Fig. 4:**
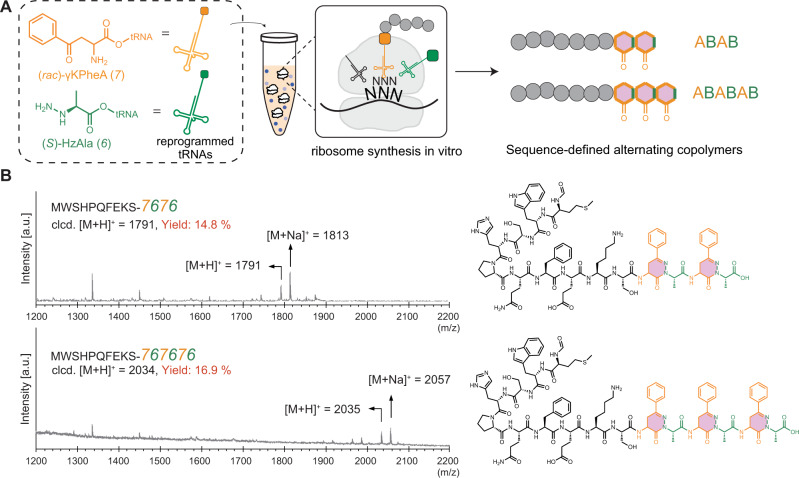
Ribosomal synthesis of alternating copolymers with a pyridazinone backbone. **A** We designed an additional amino acid, γKPheA (*7*), bearing a ketone on its γ-carbon of the sidechain, for sequential polymerization of pyridazinones bonds on a biopolymer chain. Compounds *7* and *6* were charged to tRNA^Pro1E2^(GGU) and tRNA^GluE2^(GAU) by flexizyme, respectively, and added to an in vitro transcription and translation reaction. The genetic template was designed to consecutively incorporate the monomers in an alternating fashion (ABAB- or ABABAB-type). The resulting peptides-pyridazinone hybrids were purified via the streptavidin tag (WSHPQFEK) and characterized by MALDI-TOF mass spectrometry. **B** MALDI mass spectrum of the StrepII-*7676* peptide (relative peak area: 14.8%) and its molecular structure, calculated mass: [M + H]^+^ = 1791; [M + Na]^+^ = 1813 (**C**) MALDI mass spectrum of the StrepII-*767676* (relative peak area: 16.9%) peptide and its molecular structure, calculated mass: [M + H]^+^ = 2034; [M + Na]^+^ = 2056. Spectra are representative of *n* = 3 independent experiments.

To test the limits of the sequence-defined incorporation of pyridazinone linkages in vitro, we sought to program the production of multiple alternating oligopyridazinones. To do so, we leveraged our previous design rules for Fx-mediated site-specific incorporation^20^ and synthesized cyanomethyl 2-amino-4-oxo-4-phenylbutanoate (*7*, Fig. 4). The design rules are a guide to search for non-canonical monomers compatible with Fx-mediated acylation and significantly reduce the need for ad hoc/trial-and-error exercises on designing new substrates. In brief, (i) substrates with structural similarity to Phe, (ii) substrates offering electron deficiency at the carbonyl group (acylation site), and (iii) substrates with low steric hindrance around the carbonyl facilitate the Fx-mediated tRNA-charging reaction^20^. After C-terminal extension of the Strep-tag with *7*, we envisioned that the reactive γ-keto handle could undergo a cyclocondensation reaction with the α-hydrazino acid *6* programed at the subsequent codon to form a pyridazinone linkage. Further extensions would be accomplished by sequential incorporation of *7* followed by *6* (Fig. 4).

For demonstration purposes, we designed additional plasmids (pJL1-StrepII-TI2 and pJL1-StrepII-TI3) that allow the incorporation of γ-keto amino acid *7* and (*S*)-HzAla *6* repeatedly in an alternating fashion at the C-terminus. We envisioned these monomers would produce peptides containing two or three consecutive pyridazinones, when four or six multiple incorporations are created by the ribosome, respectively. We used HzAla *6* instead of HzPhe *5* for the multiple pyridazinone bond formation, because it was more efficiently incorporated (Fig. 2C). Given the target of multiple incorporations, we also supplemented three to four times higher amounts of tRNA^Pro1E2^(GGU):*7* and tRNA^GluE2^(GAU):*6* complexes than the amount used for the single pyridazinone formation reaction. After carrying out in vitro transcription and translation reactions with the PURExpress^TM^ system, we purified the resulting oligomers, and analyzed them by MALDI-TOF mass spectrometry. In our MALDI mass spectra (Fig. 4), we observed peaks demonstrating ribosome-catalyzed synthesis of peptide-hybrid oligomers composed of multiple alternating pyridazinone linkages (see Supplementary Fig. 11 for full spectrum).

## Discussion

In this work, we demonstrated ribosome-catalyzed formation of pyridazinone linkages in vitro for the biosynthesis of pyridazinone-peptide hybrids. Our results revealed several key features relevant to the development of alternative ribosome-catalyzed chain concatenations. First, while the field of genetic code reprogramming has reported hundreds of non-canonical chemical substrates, it was previously unclear if the ribosome could polymerize non-peptide backbone structures based on γ-keto and hydrazino ester monomers. We showed that this is possible using a set of rationally designed monomers to synthesize pyridazinone bonds. Second, we verified our findings by showing that pyridazinone rings are only generated in the presence of the ribosome under the conditions used. Third, we demonstrated that the ribosome could produce oligomers composed of multiple alternating pyridazinone backbones spaced by amide bonds according to a programmed genetic template.

Our work represents a starting point for efforts to further elucidate fundamental principles underpinning molecular translation. For example, we observed different levels of translational activity, which point to future opportunities to engineer the ribosome and associated translation apparatus to work efficiently with the cyclocondensation reaction between γ-keto and α-hydrazino ester monomers. While efficiencies of target product range from ~15–40% for single to multiple pyridazinone bonds, there is room for optimism. Until the advent of Release Factor 1 deficient strains of *E. coli* about a decade ago, for example, crude extract based in vitro transcription and translation systems only installed an α-based ncAA ~20% of the time, with ~80% truncated product^50^. Yet, with technological advances, these cell-free systems are now closer to 100% incorporation efficiency^51,52^. We expect ribosome engineering platforms such as in vitro ribosome synthesis and evolution (RISE)^53,54^, and computational methods for RNA secondary structure prediction (e.g., Eterna, Rosetta stepwise Monte Carlo method)^55–58^ could lead to engineered ribosomes that are capable of forming the pyridazinone bond formation more efficiently.

Looking forward, we expect our work to motivate new directions to expand a broader spectrum of non-canonical linkages in sequence-defined polymers with the engineered translation machinery. In addition, the plasticity of the ribosome’s ancient entropy trap mechanism motivates further study by bringing into sharp relief the question of why the peptide bond was selected in the first place, given that many alternatives would have been available to not just the nascent ribosome, but to its many descendants.

## Methods

All materials were of the best grade commercially available and used without further purification: 3,5-dinitrobenzyl chloride (Sigma-Aldrich, 97%), diisopropylethylamine (DIPEA, Acros, ≥99.5%), chloroacetonitrile (Alfa Aesar, ≥98%), Boc-protected amino acids (Sigma-Aldrich, ≥98%), 2-((*tert*-butoxycarbonyl)amino)−4-oxo-4-phenylbutanoic acid (Enamine, 95%), 3-benzoylpropionic acid (Sigma-Aldrich, 99%), 4-(4-(methylthio)phenyl)−4-oxobutanoic acid (Sigma-Aldrich), 4-oxo-4-phenylbutanoic acid (Sigma-Aldrich), levulinic acid (Sigma-Aldrich, 98%), tert-butyl triphenylphosphoranylidene carbamate (Matrix Scientific, ≥95%), diethyl ketomalonate (Matrix Scientific, ≥95%), Oxone ® (Alfa Aesar), trifluoroacetic acid (Alfa Aesar, ≥99.5%). All materials were stored under the recommended storage conditions as described by the supplier. All reaction solvents were purchased from Fischer Scientific, unless otherwise specified. Anhydrous solvents (CH_2_Cl_2_, DMF, THF, MeOH, and MeCN) were obtained by using the solvent delivery system from Vacuum Atmosphere Company and stored over 3 Å molecular sieves under argon. NMR solvents (CDCl_3_, DMSO-d_6_, and MeOD) were purchased from Cambridge Isotope Laboratories or Sigma-Aldrich. All the oligonucleotides used in this research were purchased from Integrated DNA Technologies (IDT) and used as received.

General synthetic procedure A: Formation of cyanomethyl ester and/or Boc deprotection: To a solution of carboxylic acid (1 equiv.) triethylamine (1.5 equiv.), chloroacetonitrile (1.2 equiv.) and dichloromethane (1.0 M) were added and stirred overnight. After stirring for 16 h at room temperature, the reaction mixture was diluted with EtOAc and washed with HCl (0.5 M aq.), NaHCO_3_ (4% (w/v) in water), brine, and dried over MgSO_4_. The organic phase was concentrated to provide the crude product. Flash column chromatography was performed when necessary. For deprotection of the Boc group, 0.5 mL of TFA was added dropwise at 0 °C and the solution was stirred at room temperature for 1 h.

General synthetic procedure B: Formation of dinitrobenzyl esters and/or Boc deprotection: To a solution of carboxylic acid (1 equiv.), dichloromethane (1.0 M), triethylamine (1.5 equiv.), and 3,5-dinotrobenzyl chloride (1.2 equiv.) were added. After stirring for 16 h at room temperature, the reaction mixture was diluted with EtOAc and washed with HCl (0.5 M aq.), NaHCO_3_ (4% (w/v) in water), brine, and dried over MgSO_4_. The organic phase was concentrated to provide the crude product. The product was purified by flash column chromatography. The resulting fraction containing product was collected in a 100 mL flask and the solvent was removed under reduced pressure. 2 mL of HCl (4 N in anhydrous dioxane) was added and stirred for 1 h at room temperature. The resulting product was transferred to a 20 mL glass vial and dried under a high vacuum overnight to give the final product.

General synthetic procedure C: Formation of 4-((2-aminoethyl)carbamoyl)benzyl thioates & Boc deprotection: To a solution of carboxylic acid (1.4 equiv.), tert-butyl 2-[4-(mercaptomethyl)benzamido]ethyl carbamate (Boc-ABT)^59^ (1.0 equiv), 4-dimethylaminopyridine (DMAP) (2.8 equiv) in DCM was added *N*-(3-dimethylaminopropyl)-*N*′-ethylcarbodiimide hydrochloride (EDC⋅HCl) (2.8 equiv) at 0 °C, and the reaction mixture was then warmed to room temperature and stirred for 3 h. To this was added 1 N HCl(aq) and the layers were separated. The aqueous layer was extracted with DCM (x2), and the combined organic layers were dried (MgSO_4_) and concentrated under reduced pressure. The crude was purified by flash column chromatography (EtOAc/n-Hexane) to furnish the Boc-protected products. The deprotection was achieved upon treatment with a 4 M solution of HCl in 1,4-dioxane, and the resulting products were used without further purification and characterization.

### Preparation of Fx DNA template

Extension: 0.5 μL of 200 μM Fx_F primer and 0.5 μL of 200 μM of Fx_R1 primer (eFx_R1, dFx_R1, and aFx_R1 were used for eFx, dFx, and aFx preparation, respectively) were added to 99 μL of a master mix containing 9.9 μL of 10X PCR buffer (500 mM KCl, 100 mM Tris-HCL (pH 9.0), and 1% of Triton X-100), 0.99 μL of 250 mM MgCl_2_, 4.95 μL of 5 mM dNTPs, 0.66 μL of Taq DNA polymerase (NEB), and 82.5 μL of water in a PCR tube. The thermocycling conditions were: 1 min at 95 °C followed by five cycles of 50 °C for 1 min and 72 °C for 1 min. The sizes of products were checked in 3% (w/v) agarose gel. PCR amplification: 5 μL of of the extension product was used as a PCR template. 200 μL of 5X OneTaq® Standard buffer, 20 μL of 10 mM dNTP, 5 μL of 200 μM Fx_T7F primer, and 5 μL of 200 μM Fx_R2 (eFx_R2, dFx_R2, and aFx_R2 were used for eFx, dFx, and aFx preparation, respectively), 10 μL of OneTaq® polymerase and 755 μL of nuclease-free water was mixed in a 1.5 mL microcentrifuge tube. The mixture was transferred to 10 PCR tubes and the DNA was amplified by the following thermocycling conditions: 1 min at 95 °C followed by 12 cycles of 95 °C for 40 s and 50 °C for 40 s, and 72 °C for 40 s. Products were checked in 3% (w/v) agarose gel.

### Preparation of tRNA DNA template

Extension: 0.5 μL of 200 μM fMetE-F primer and 0.5 μL of 200 μM of fMetE-R1 primer (Pro1E2-F and Pro1E2-R1 were used for Pro1E2 tRNA preparation) were used for tRNA template extension under the same condition described above for the flexizyme extension. PCR amplification: 5 μL of the extension product was directly used for the next round amplification reaction with the addition of 0.5 μL of 200 μM fMetE-F, 0.5 μL of 200 μM fMet-R2, 2 μL of 5X OneTaq, and 2 μL of nuclease-free water. The thermocycling conditions were: 1 min at 95 °C followed by 12 cycles of 95 °C for 30 sec, 50 °C for 30 sec, and 72 °C for 30 sec. 5 μL of the first PCR product was mixed with 5 μL of 200 μM fMetE_F and fMet-R3, 200 μL of 5X HF buffer, 10 μL of Phusion polymerase (NEB), 20 μL of 10 mM dNTPs, and 755 μL of water. The thermocycling conditions were: 1 min at 95 °C followed by 35 cycles of 95 °C for 5 sec, 60 °C for 10 sec, and 72 °C for 10 sec, and final elongation at 72 °C for 1 min. The sizes of products were checked in 3% (w/v) agarose gel.

### DNA template precipitation

PCR products were extracted using phenol/chloroform/isoamyl alcohol and precipitated with EtOH. Samples were dried at room temperature for 5 min and resuspended in 50 μL nuclease-free water. DNA concentrations were determined from the absorbance measured on a Thermo Scientific NanoDrop 2000C spectrophotometer.

### In vitro transcription

Flexizymes and tRNAs were prepared using a HiScribe T7 high-yield RNA synthesis kit (NEB). For in vitro transcription, 5 μg of DNA template was used with 10 μL of each of 10X T7 Reaction Buffer, ATP, CTP, GTP, UTP, T7 RNA polymerase mix, and nuclease-free water upto 100 μL. The mixture was incubated at 37 °C overnight.

### Digestion of DNA templates

The DNA templates were removed by adding 5 μL of DNase I (NEB) and 20 μL of DNase I reaction buffer into the 100 μL of transcription reaction products. The reaction mixture was incubated for 1 h at 37 °C.

### Purification of in vitro transcribed RNA

The digested transcription reactions were mixed with 100 μL 2x RNA loading dye, and loaded onto a 15% TBE-Urea gel (Invitrogen). The gel was run in Tris-Borate-EDTA (89 mM Tris, 89 mM boric acid, 2 mM EDTA, and pH 8.3) buffer at 160 V for 2.5 h at room temperature. The gel was placed on a cling film covering a 20 cm × 20 cm TLC silica gel glass plate (EMD Millipore) coated with a fluorescent indicator and the transcribed RNAs were visualized by irradiating with a UV lamp (260 nm). The RNA products were excised from the gel and added to 2 mL of water. The gels were crushed and then shaken in the cold room for 4 h. The gels were transferred to a centrifugal filter (EMD Millipore) and centrifuged at 4000 × *g* for 2 min. The flow-through was collected and added to the solution of 120 μL of 5 M NaCl and 5 mL of 100% EtOH. The solution was placed at −20 °C for 16 h and centrifuged at 15,000 × *g* for 45 min at 4 °C. The supernatant was removed and the pellet was dried for 5 min at room temperature. The dried RNA pellet was dissolved in nuclease-free water and the concentration was determined spectrophotometrically.

### Precipitation of tRNA

Into a 1.5 mL microcentrifuge tube containing 100 μL of EtOH and 40 μL of 0.3 M NaOAc (pH 5.2), the mixture from the coupling reaction was added and mixed to quench the reaction. The mixture was centrifuged at 21,000 × *g* for 15 min at room temperature and the supernatant was removed. The RNA pellet was washed with 50 μL of 70% (v/v) ethanol containing 0.1 M NaOAc (pH 5.2) was resuspended into the solution by vortexing and subsequently centrifuged at 21,000 × *g* for 5 min at room temperature. The washing step was repeated twice. After the supernatant was discarded, the pellet was resuspended in 50 μL of 70% (v/v) ethanol resuspended and centrifuged at 21,000 × *g* for 3 min at room temperature. The supernatant was removed and the pellet was dissolved by 1 μL of 1 mM NaOAc (pH 5.2).

### Fx-mediated acylation reaction

For mihx acylation, 1 μL of 0.5 M HEPES (pH 7.5) or bicine (pH 8.8), 1 μL of 10 μM microhelix, and 3 μL of nuclease-free water were mixed in a PCR tube with 1 μL of 10 μM eFx, dFx, and aFx, respectively. The mixture was heated for 2 min at 95 °C and cooled down to room temperature over 5 min. 2 μL of 300 mM MgCl_2_ was added to the cooled mixture and incubated for 5 min at room temperature. Followed by the incubation of the reaction mixture on ice for 2 min, 2 μL of 25 mM activated ester substrate in DMSO was then added to the reaction mixture. The reaction mixture was further incubated for 6–48 h on ice in a cold room. The microhelix RNA was obtained from Integrated DNA Technologoes (IDT) and used as received (mihx: rGrGrCrUrCrUrGrUrUrCrGrCrArGrArGrCrCrGrCrCrA). For tRNA acylation, 2 μL of buffer (0.5 M HEPES (pH 7.5) or 0.5 M bicine (pH 8.8), 2 μL of 250 μM tRNA, 2 μL of 250 μM of an Fx selected on the microhelix experiment, and 6 μL of nuclease-free water were mixed in a PCR tube. The mixture was heated for 2 min at 95 °C and cooled down to room temperature over 5 min. Four microliters of 300 mM MgCl_2_ was added to the cooled mixture and incubated for 5 min at room temperature. Followed by the incubation of the reaction mixture on ice for 2 min, 4 μL of 25 mM activated ester substrate in DMSO was then added to the reaction mixture. The reaction mixture was further incubated for the optimal time determined in the microhelix experiment on ice in a cold room.

### Reporting summary

Further information on research design is available in the Nature Research Reporting Summary linked to this article.

## Supplementary information

Supplementary Information

Reporting Summary

## Data Availability

All data generated or analysed during this study are included in this published article (and its supplementary information files). Direct correspondence to Joongoo Lee, Eric V. Anslyn, or Michael C. Jewett. Source data are provided with this paper.

## Source data

Source Data
