# Supplementary material for: Ribosome-mediated biosynthesis of pyridazinone oligomers in vitro
Source: Nat Commun. 2022 Oct 24;13:6322. doi: 10.1038/s41467-022-33701-2 (PMC9592601; doi:10.1038/s41467-022-33701-2)
Supplement: Supplementary file 1 — Supplementary Information [file 41467_2022_33701_MOESM1_ESM.pdf]

Supplementary Information

Ribosome-mediated biosynthesis of pyridazinone oligomers *in vitro*

Joongoo Lee[1,2,†,*], Jaime N. Coronado[3†], Namjin Cho[2], Jongdoo Lim[3], Brandon M. Hosford[3], Sangwon Seo[4,5], Do Soon Kim[1], Camila Kofman[1], Jeffrey S. Moore[6,7], Andrew D. Ellington[8], Eric V. Anslyn[*,3], Michael C. Jewett[*,1,9,10,11,12,13]

[1]Department of Chemical and Biological Engineering, Northwestern University, Evanston, IL, USA
[2]Department of Chemical Engineering, Pohang University of Science and Technology (POSTECH), Pohang, 37673, Republic of Korea
[3]Department of Chemistry, University of Texas at Austin, Austin, TX, USA
[4]Department of Chemistry, Korea Advanced Institute of Science and Technology (KAIST), Daejeon 34141, Republic of Korea.
[5]Center for Catalytic Hydrocarbon Functionalizations, Institute for Basic Science (IBS), Daejeon 34141, Republic of Korea
[6]Department of Chemistry, University of Illinois at Urbana-Champaign, Urbana, IL, USA
[7]Beckman Institute for Advanced Science and Technology, University of Illinois at Urbana-Champaign, Urbana, Illinois 61801, USA
[8]Department of Chemistry and Biochemistry, Institute for Cellular and Molecular Biology, University of Texas at Austin, Austin, TX, USA
[9]Interdisplinary Biological Sciences Graduate Program
[10]Chemistry of Life Processes Institute
[11]Robert H. Lurie Comprehensive Cancer Center
[12]Simpson Querrey Institute
[13]Center for Synthetic Biology, Northwestern University and Biological Engineering, 2145 Sheridan Road, Evanston, IL 60208, USA.

[†]These authors contributed equally to this work: Joongoo Lee and Jaime N. Coronado

*Correspondence and request for materials should be addressed to: jgoolee@postech.ac.kr, anslyn@austin.utexas.edu, and m-jewett@northwestern.edu.

Table of Contents

Materials and Methods 4

Synthetic Procedures 5

Synthesis of γ-keto substrates (1-4) 5

Synthesis of oxaziridine 6

Synthesis of HzPhe-CME HCl (5) 7

Synthesis of HzAla-ABT (6) 9

Synthesis of AOP (7) 10

In vitro synthesis of pyridazinone 12

LC-MS analysis of pyridazinone 12

Preparation of standard peptides 12

Supplementary Figures 14

Supplementary Figure 1. Acylation of microhelix with substrates 1-7**.** 14
Supplementary Figure 2. Characterization of the pyridazinone products. 15
Supplementary Figure 3. The relative percent yields were determined using the peak areas. 16
Supplementary Figure 4. Control experiments for pyridazinone bond reactions. 17
Supplementary Figure 5. Ribosome-mediated synthesis of pyridazinone derivatives with HzPhe (**5**). 18
Supplementary Figure 6. Ribosome-mediated synthesis of pyridazinone derivatives with HzAla (6). 19
Supplementary Figure 7. Four possible mechanisms of pyridazinone bond formation in the ribosome. 20
Supplementary Figure 8. Monitoring the pyridazinone formation reaction. 21
Supplementary Figure 9. Estimated yields of pyridazinone-based peptides. 22
Supplementary Figure 10. Effect of engineered translational machinery on the pyridazinone bond formation with 1 and 5. 23
Supplementary Figure 11. Characterization of the alternating oligomers with a pyridazinone backbone. 24

^{1}H and ^{13}C NMR Spectra 25

Supplementary Figure 12. ^{1}H NMR (400 MHz, $CDCl_3$) of **1**. 25
Supplementary Figure 13. ^{13}C NMR (126 MHz, $CDCl_3$) of **1**. 25
Supplementary Figure 14. ^{1}H NMR (500 MHz, CD_3OD) of **2**. 26
Supplementary Figure 15. ^{13}C NMR (125 MHz, CD_3OD) of **2**. 26
Supplementary Figure 16. ^{1}H NMR (500 MHz, CD_3OD) of **3a**. 27
Supplementary Figure 17. ^{13}C NMR (125 MHz, CD_3OD) of **3a**. 27
Supplementary Figure 18. ^{1}H NMR (400 MHz, $CDCl_3$) of **3b**. 28
Supplementary Figure 19. ^{13}C NMR (125 MHz, DMSO-d6) of **3b**. 28
Supplementary Figure 20. ^{1}H NMR (500 MHz, CD_3OD) of **4**. 29
Supplementary Figure 21. ^{13}C NMR (125 MHz, CD_3OD) of **4.** 29
Supplementary Figure 22. ^{1}H NMR (500 MHz, $CDCl_3$) of Boc-**5**. 30
Supplementary Figure 23. ^{13}C NMR (126 MHz, $CDCl_3$) of Boc-**5.** 30
Supplementary Figure 24. ^{1}H NMR (500 MHz, $CDCl_3$) of Boc-Ala-DNB. 31
Supplementary Figure 25. ^{13}C NMR (126 MHz, $CDCl_3$) of Boc-Ala-DNB. 31
Supplementary Figure 26. ^{1}H NMR (500 MHz, DMSO-d_6) of Ala-DNB. 32
Supplementary Figure 27. ^{13}C NMR (126 MHz, DMSO-d_6) of Ala-DNB. 32
Supplementary Figure 28. ^{1}H NMR (500 MHz, $CDCl_3$) of Boc-**6a**. 33
Supplementary Figure 29. ^{13}C NMR (126 MHz, $CDCl_3$) of Boc-**6a**. 33
Supplementary Figure 30. ^{1}H NMR (400 MHz, CD_3OD) of **6b**. 34
Supplementary Figure 31. ^{13}C NMR (125 MHz, DMSO-d6) of **6b**. 34
Supplementary Figure 32. ^{1}H NMR (500 MHz, $CDCl_3$) of Boc-**7**. 35
Supplementary Figure 33. ^{13}C NMR (126 MHz, $CDCl_3$) of Boc-**7**. 35

Supplementary Figure 34. ^1H NMR (400 MHz, DMSO-d6) of **7**. 36
Supplementary Figure 35. ^{13}C NMR (125 MHz, DMSO-d6) of **7**. 36

High Resolution Mass Spectrometry **37**
Supplementary Figure 36. HRMS of Cyanomethyl 4-oxo-4-phenylbutanoate (**1**). 37
Supplementary Figure 37. HRMS of γKPheSMe-CME (**2**). 37
Supplementary Figure 38. HRMS of γKMe-DNBE (**3a**). 37
Supplementary Figure 39. HRMS of γKMe-ABT-Boc (**3b**). 38
Supplementary Figure 40. HRMS of γKEt-ABT-Boc (4). 38
Supplementary Figure 41. HRMS of Boc-HzPhe-CME (Boc-**5**). 38
Supplementary Figure 42. HRMS of HzPhe-CME (**5**). 39
Supplementary Figure 43. HRMS of Boc-Ala-DNB. 39
Supplementary Figure 44. HRMS of Ala-DNB. 39
Supplementary Figure 45. HRMS of Boc-HzAla-DNB (Boc-**6a**). 40
Supplementary Figure 46. HRMS of Ala-DNB (**6a**). 40
Supplementary Figure 47. HRMS of Boc-HzAla-ABT-Boc (**6b**). 40
Supplementary Figure 48. HRMS of Boc-AOP-CME (Boc-**7**). 41
Supplementary Figure 49. HRMS of AOP-CME (**7**). 41

Plasmid map **42**

List of primers **44**

References **45**

Materials and Methods

Mass spectra were recorded on a Bruker Rapiflex, Bruker Autoflex, AmaZon SL, or Waters Q-TOF Ultima for electron-spray ionization (ESI) and Impact-II or Waters 70-VSE for electron impact (EI). High resolution mass spectrometry (HRMS) analysis was performed by the University of Texas, Pohang University of Science and Technology (POSTECH), or Korea Advanced Institute of Science and Technology (KAIST) Mass Spectrometry Facility using the 6530 Accurate Mass Q-TOF LC/MS system from Agilent Technologies. MALDI mass spectra were processed using the FlexControl v2.0 (Bruker) software and with smoothening and baseline substraction. ^{1}H and ^{13}C NMR spectra were collected either from Northwestern University, the University of Texas at Austin, POSTECH, or KAIST NMR facility using the Bruker AVANCE III HD 500 MHz cryoprobe NMR spectrometer (NIH grant number: 1 S10 OD021508-01) and processed by TopSpin (4.0.9) or MestReNova (v14.2.2). Chemical shifts, denoted in ppm, are assigned relative to the residual NMR solvent peaks. Silica gel flash chromatography was performed using 0.035-0.070 mm, 60 Å silica purchased from Acros. Thin layer chromatography was performed using glass silica plates coated with fluorescent indicator (F254) purchased from Merck. Sand, sodium chloride, sodium bicarbonate, potassium carbonate, concentrated hydrochloric acid, and sodium hydroxide pellets were purchased from Fischer Scientific. The 3Å molecular sieves (4 to 8 mesh, Acros) were activated at 170ºC for at least 24 hours in a vacuum oven and stored in a desiccator.

Synthetic Procedures

Synthesis of γ-keto substrates (1-4)

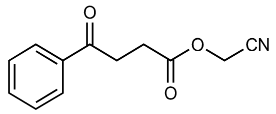

Cyanomethyl 4-oxo-4-phenylbutanoate (**1**). Prepared according to the general procedure A using 3-benzoylpropionic acid (200 mg, 1.12 mmol, 1.00 equiv.). A solution of 4-oxo-4-phenylbutanoic acid was dissolved in CH_2Cl_2 (1.12 mL, 1.00 M) and treated with triethylamine (782 µL, 5.61 mmol, 5.00 equiv.) dropwise at 0ºC under an inert atmosphere followed by chloroacetonitrile (214 µL, 3.37 mmol, 3.0 equiv.). Upon complete addition, the reaction was allowed to warm to room temperature and stirred for 18 hours. Upon reaction completion as determined by TLC, the reaction was concentrated *in vacuo* and the crude material was purified by silica gel flash chromatography (25% EtOAc/Hexanes) to yield the pure product as a clear oil (190 mg, 875 µmol, 77.9% yield). R_f = 0.27 (25% EtOAc/Hexanes). ^{1}H NMR (400 MHz, $CDCl_3$) δ 7.99 – 7.94 (m, 2H), 7.61 – 7.55 (m, 1H), 7.50 – 7.43 (m, 2H), 4.75 (s, 2H), 3.35 (t, *J* = 6.4 Hz, 2H), 2.84 (t, *J* = 6.6 Hz, 2H). ^{13}C NMR (126 MHz, $CDCl_3$) δ 197.51, 171.55, 136.24, 133.60, 128.81, 128.12, 114.51, 48.59, 33.17, 27.67. HRMS (ESI/Q-TOF) calc. for $C_{12}H_{11}NO_3$ $[M + Na]^+$ = 240.0631; Found 240.0631.

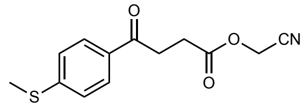

Cyanomethyl 4-(4-(methylthio)phenyl)-4-oxobutanoate (**2**). Prepared according to general procedure A using 4-(4-(methylthio)phenyl)-4-oxobutanoic acid (224 mg, 1 mmol), triethylamine (167 µL, 1.2 mmol), chloroacetonitrile (95 µL, 1.5 mmol) and dichloromethane (0.5 mL). The product was obtained as a yellow oil (205 mg, 78% yield). ^{1}H NMR (500 MHz, CD_3OD) δ 7.94 (d, *J* = 8.5 Hz, 2H), 7.35 (d, *J* = 8.5 Hz, 2H), 3.37 (t, *J* = 6.7 Hz, 2H), 2.82 (t, *J* = 6.7 Hz, 2H).^{13}C NMR (125 MHz, CD_3OD) 197.5, 171.8, 132.2, 130.0, 128.1 (2C), 124.6 (2C), 114.8, 32.5, 27.0, 14.4, 13.1. HRMS (ESI/Q-TOF) calc. for $C_{13}H_{13}NO_3S$ $[M + Na]^+$ = 286.0513; Found 286.0511.

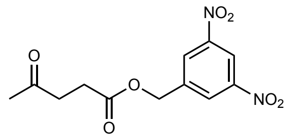

3,5-dinitrobenzyl 4-oxopentanoate (**3a**). Prepared according to general procedure B using 4-oxopentanoic acid (116 mg, 1 mmol), triethylamine (167 µL, 1.2 mmol), 3,5-dinitorbenzyl chloride (324.8 mg, 1.5 mmol) and dichloromethane (0.5 mL). The product was obtained as a white powder (201 mg, 65% yield). ^{1}H NMR (500 MHz, CD_3OD) δ 8.96 (s, 1H), 8.65 (d, *J* = 1.7 Hz, 2H), 5.36 (s, 2H), 2.87 (t, 2H), 2.67 (t, 2H), 2.18 (s, 3H). ^{13}C NMR (CD_3OD, 125 MHz) δ 208.1, 172.6, 148.5, 140.9, 127.5 (2C), 117.6 (2C), 63.7, 37.2, 28.1, 27.3. HRMS (ESI/Q-TOF) calc. for $C_{12}H_{12}N_2O_7$ $[M + Na]^+$ = 319.0542; Found 319.0540.

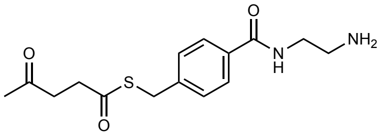

3,5-dinitrobenzyl 4-oxohexanoate (**3b**). Prepared according to general procedure C using levulinic acid (98 mg, 0.84 mmol), Boc-ABT (186 mg, 0.6 mmol), DMAP (205 mg, 1.7 mmol), EDC·HCl (322 mg, 1.7 mmol) and DCM (4.0 mL). Purification by flash column chromatography (80% EtOAc in n-Hex) afforded the corresponding Boc-protected product as a white solid (146 mg, 60%). The deprotection was achieved upon treatment with 4M solution of HCl in 1,4-dioxane, and the resulting product was used without further purification and characterization. Boc-3b: ^{1}H NMR (400 MHz, $CDCl_3$) δ 7.73 (d, J = 8.0 Hz, 2H), 7.30 (d, J = 8.0 Hz, 2H), 7.25 (br s, 1H), 5.10 (br s, 1H), 4.12 (s, 2H), 3.54 – 3.50 (m, 2H), 3.39 – 3.35 (m, 2H), 2.87 – 2.77 (m, 4H), 2.18 (s, 3H), 1.41 (s, 9H). ^{13}C NMR (125 MHz, DMSO-d6) 206.7, 197.6, 166.8, 141.8, 133.2 (2C), 128.9(2C), 128.0 (2C), 39.0 37.9 (2C), 37.5 (2C), 32.2 (2C), 29.9 (3C) ppm. HRMS (ESI/Q-TOF) calc. for $C_{20}H_{28}N_2O_5S$ $[M + K]^+$ = 447.1356; Found 447.1365.

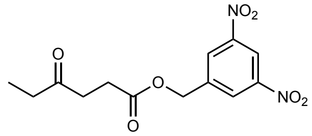

S-(4-((2-aminoethyl)carbamoyl)benzyl) 4-oxopentanethioate (**4**). Prepared according to general procedure B using 4-oxo-4-phenylbutanoic acid (178 mg, 1 mmol), triethylamine (167 μL, 1.2 mmol), chloroacetonitrile (95 μL, 1.5 mmol) and dichloromethane (0.5 mL). The product was obtained as a white powder (158.1 mg, 51 %). ^{1}H NMR (500 MHz, MeOD) δ 8.96 (s, 2H), 8.65 (s, 2H), 5.36 (s, 2H), 2.83 (t, J = 6.3 Hz, 2H), 2.68 (t, J = 6.0 Hz, 2H), 2.83 (t, J = Hz, 2H), 2.52 (q, J = 7.3 Hz, 2H), 1.03 (t, J = 7.3 Hz, 3H). ^{13}C NMR (125 MHz, MeOD) 210.5, 172.6, 148.6 (2C), 140.9, 127.5 (2C), 117.6, 63.7, 36.0, 34.9, 27.3, 6.5 (2C) ppm. HRMS (ESI/Q-TOF) calc. for $C_{13}H_{14}N_2O_7$ $[M + Na]^+$ = 333.0699; Found 333.0684.

Synthesis of oxaziridine

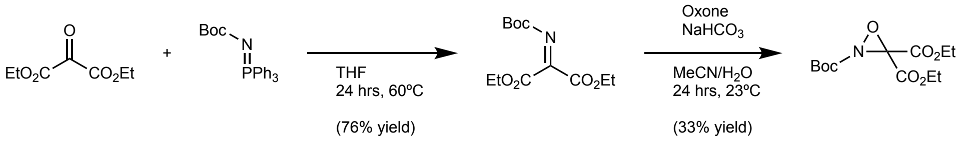

2-(*tert*-butyl) 3,3-diethyl 1,2-oxaziridine-2,3,3-tricarboxylate (Boc-Ozd)[2, 3] Oxaziridine was synthesized using previously reported methods.[2] A pressure flask containing *N*-Boc-iminophosphorane (9.76 g, 25.9 mmol, 1.00 equiv.) in 26 mL of anhydrous THF was treated with diethyl ketomalonate (3.94 mL, 25.9 mmol, 1.00 equiv.). The reaction mixture was sealed and stirred at 60 ºC. After 24 hours, the mixture was cooled and concentrated *in vacuo*. The light-yellow oil was redissolved in warm toluene and Ph_3PO was precipitated with pentane. The supernatant was filtered, and the filtrate was concentrated *in vacuo*. This process was repeated 3-4 times or until no more Ph_3PO precipitate was observed. Concentration *in vacuo* gave the *N*-Boc-iminodiethylmalonate as a light-yellow oil (5.35 g, 19.6 mmol, 76% yield. Without further

purification, the *N*-Boc-iminodiethylmalonate was dissolved in 74 mL of MeCN and 48 mL of H_2O before addition of a solid mixture of Oxone (28.9 g, 47.0 mmol, 2.40 equiv.) and $NaHCO_3$ (6.09 g, 72.5 mmol, 3.70 equiv.). The reaction mixture was stirred for 5 hours before addition of another portion of Oxone (28.9 g, 47.0 mmol, 2.40 equiv.). The reaction was stirred for an additional 19 hours under ambient conditions. The heterogeneous mixture was diluted with 300 mL of H_2O and extracted 3 times with CH_2Cl_2. The combined organic layers were dried over $MgSO_4$, filtered, and concentrated *in vacuo*. Purification by silica gel flash chromatography (6:2.5:0.5 Hexane/CH_2Cl_2/Et_2O) to yield a light-yellow oil (1.84 g, 6.37 mmol, 32.5% yield). Spectra matched literature.

Synthesis of HzPhe-CME HCl (**5**)

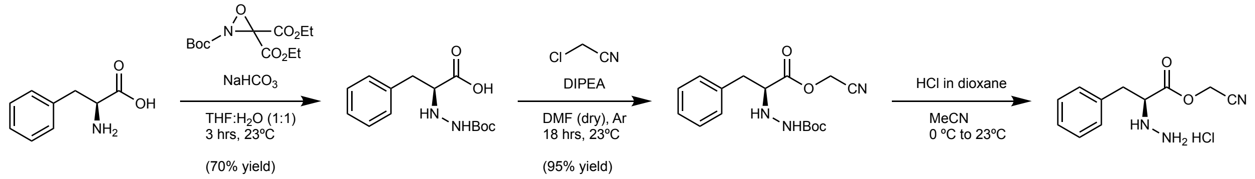

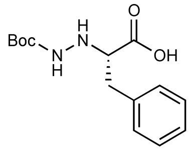

((*tert*-butoxycarbonyl)amino)-*L*-phenylalanine.[1] To a biphasic mixture of L-phenylalanine (357 mg, 2.16 mmol, 1.00 equiv.) in THF (20 mL) and satd. $NaHCO_3$ (aq) (20 mL) was added Boc-Ozd (625 mg, 2.16 mmol, 1.00 equiv.) dropwise. The reaction was allowed to stir for 4 hours under ambient conditions before treatment with ethylenediamine (550 µL, 8.21 mmol, 3.8 equiv.). After 5 minutes, the reaction mixture was acidified to pH ~1 using 1M HCl (aq), extracted with EtOAc, and concentrated *in vacuo* to yield a white solid that quickly turned light brown. Trituration with EtOAc and hexanes gave the desired product as a white solid (424 mg, 1.51 mmol, 70.0% yield). Spectra matched literature. ^1H NMR (400 MHz, DMSO-d_6) δ 8.28 (s, 1H), 7.30 – 7.15 (m, 5H), 3.68 (t, J = 6.3 Hz, 1H), 2.84 (d, J = 6.3 Hz, 2H), 1.37 (s, 9H).

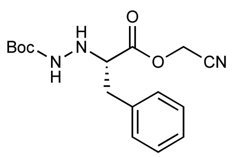

tert-butyl (*S*)-2-(1-(cyanomethoxy)-1-oxo-3-phenylpropan-2-yl)hydrazine-1-carboxylate. A solution of Boc-HzPhe-OH (250 mg, 892 µmol, 1.00 equiv.) in 1.5 mL of anhydrous DMF was treated with diisopropylethylamine (171 µL, 981 µmol, 1.10 equiv.) then chloroacetonitrile (62.2 µL, 981 µmol, 1.10 equiv.). The reaction mixture was stirred under an inert atmosphere at room temperature for 18 hours then concentrated *in vacuo*. The crude material was redissolved in EtOAc and purified by silica gel flash chromatography (30% EtOAc/Hexanes) to yield a clear oil (270 mg, 845 µmol, 94.8%). R_f = 0.30 (30% EtOAc/Hexanes). ^1H NMR (500 MHz, $CDCl_3$) δ 7.37 – 7.21 (m, 5H), 6.31 (s, 1H), 4.71 (d, J = 2.8 Hz, 2H), 4.08 (s, 1H), 4.05

(t, J = 7.0 Hz, 1H), 3.06 (qd, J = 14.0, 7.0 Hz, 2H), 1.45 (s, 9H). ^{13}C NMR (126 MHz, $CDCl_3$) δ 171.20, 156.54, 135.77, 129.21, 128.87, 127.33, 114.10, 81.25, 64.29, 48.75, 36.86, 28.33. HRMS (ESI/Q-TOF) calc. for $C_{16}H_{21}N_3O_4$ $[M + Na]^+$ = 342.1424; Found 342.1435.

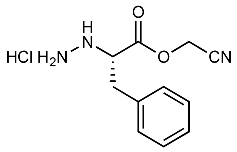

cyanomethyl amino-*L*-phenylalaninate HCl. (**5**) Prepared according to general procedure A using Boc-HzPhe-CME. To a vial containing Boc-hydPhe-CME (100 mg, 313 μmol, 1.00 equiv.) in 2mL of MeCN was added dropwise 4 M HCl in dioxane (235 μL, 939 μmol, 3.00 equiv.) at 0 ºC under argon. The solution was stirred at 0 ºC for 2 hours and concentrated *in vacuo*. The residue was dissolved in a minimal amount of MeCN and the product was precipitated by addition of Et_2O. The solids were washed 3 times with 15% MeOH in Et_2O and dried *in vacuo* to yield the hydrochloride salt as a white solid which was used without further purification. HRMS (ESI/Q-TOF) calc. for $C_{11}H_{13}N_3O_2$ $[M + H]^+$ = 220.1081; Found 220.1081.

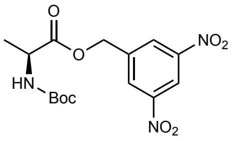

3,5-dinitrobenzyl (*tert*-butoxycarbonyl)-*L*-alaninate (Boc-Ala-DNB). Prepared using General Method B. Purification by silica gel flash chromatography (20% EtOAc/Hexanes) gave the title compound as a yellow tinted white solid (0.908 g, 82% yield). R_f = 0.21 (20% EtOAc/Hexanes). ^1H NMR (500 MHz, $CDCl_3$) δ 9.01 (t, J = 2.1 Hz, 1H), 8.56 (d, J = 2.0 Hz, 2H), 5.41 – 5.31 (m, 2H), 4.96 (br s, 1H), 4.44 – 4.34 (m, 1H), 1.44 (d, J = 7.3 Hz, 3H), 1.43 (s, 9H). ^{13}C NMR (126 MHz, $CDCl_3$) δ 173.16, 155.29, 148.85, 140.24, 127.91, 118.75, 80.49, 64.52, 49.44, 28.39, 18.26. HRMS (ESI/Q-TOF) calc. for $C_{15}H_{19}N_3O_8$ $[M + Na]^+$ = 392.1064; Found 392.1065.

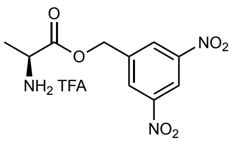

3,5-dinitrobenzyl *L*-alaninate TFA (Ala-DNB). To a vial containing Boc-Ala-DBE (500 mg, 1.35 mmol, 1.0 equiv.) dissolved in CH_2Cl_2 (4.0 mL) was added TFA (1.0 mL, 13.5 mmol, 10.0 equiv.) dropwise at 0ºC. After complete addition, the reaction was warmed to room temperature and stirred for 30 min upon which TLC analysis confirmed reaction completion. The volatiles were removed *in vacuo* and the residue was triturated with Et_2O to yield the pure product as a white solid. (483 mg, 93% yield).

^1H NMR (500 MHz, DMSO) δ 8.82 (t, J = 2.1 Hz, 1H), 8.73 (d, J = 2.1 Hz, 2H), 8.58 (s, 3H), 5.50 (d, J = 1.9 Hz, 2H), 4.26 (q, J = 7.2 Hz, 1H), 1.46 (d, J = 7.2 Hz, 3H). ^{13}C NMR (126 MHz, DMSO-d6) δ 169.70, 148.14, 139.74, 128.45, 118.41, 64.97, 47.97, 15.71. HRMS (ESI/Q-TOF) calc. for $C_{10}H_{11}N_3O_6$ $[M +H]^+$ = 270.0721; Found 270.0727.

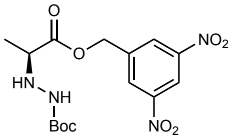

tert-butyl (*S*)-2-(1-((3,5-dinitrobenzyl)oxy)-1-oxopropan-2-yl)hydrazine-1-carboxylate (Boc-6a). To a biphasic mixture of L-alanine-DBE TFA (132 mg, 346 µmol, 1.00 equiv.) in THF (2.5 mL) and satd. $NaHCO_3$ (aq) (2.5 mL) was added oxaziridine (100 mg, 346 µmol, 1.00 equiv.) dropwise. The reaction was allowed to stir for 120 min under ambient conditions before the reaction mixture was extracted three times with 20 mL of EtOAc. The combined organic layers were dried over anhydrous Na_2SO_4 and concentrated *in vacuo* to yield an oil. The product was purified by silica gel flash chromatography (35% EtOAc/Hexanes) to yield the product as a yellow-tinted oil (114 mg, 297 µmol, 85.8% yield).

R_f = 0.24 (35% EtOAc/Hexanes). ^{1}H NMR (500 MHz, $CDCl_3$) δ 8.96 – 8.93 (m, 1H), 8.55 – 8.51 (m, 2H), 6.40 (s, 1H), 5.34 (s, 2H), 4.90 (s, 1H), 4.41 – 4.15 (m, 1H), 1.51 – 1.28 (m, 12H). ^{13}C NMR (126 MHz, $CDCl_3$) δ 173.21, 156.67, 148.65, 140.25, 127.92, 118.58, 81.03, 64.27, 58.44, 28.23, 15.91. HRMS (ESI/Q-TOF) calc. for $C_{15}H_{20}N_4O_8$ $[M + Na]^+$ = 407.1173; Found 407.1180.

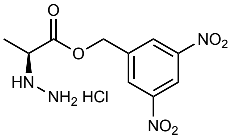

3,5-dinitrobenzyl amino-*L*-alaninate HCl **(6a).** A dram vial equipped with a stirring rod was charged with Boc-hydAla-DBE (20.0 mg, 52.0 µmol, 1.00 equiv.) dissolved in 500 µL of anhydrous CH_2Cl_2. The vial was placed in an ice bath and cooled to 0 ºC before it was treated with 50 µL of TFA (649 µmol, 12.5 equiv.). After removing the reaction from the ice bath and allowing it to slowly warm to 23 ºC, the solution was stirred for 120 minutes then concentrated *in vacuo*. The residue was redissolved in ~200 µL of Et_2O and treated with 200 µL of 2N HCl in Et_2O to produce a cloudy white heterogeneous mixture. The resulting solids were allowed to settle, and the supernatant was carefully removed via pipette. The precipitate was washed 3 more times with ~1.0 mL of Et_2O before being dried *in vacuo* to yield the HCl product as a light-yellow solid. The compound was used without further purification and characterization. HRMS (ESI/Q-TOF) calc. for $C_{10}H_{12}N_4O_6$ $[M + H]^+$ = 285.0830; Found 285.0833.

Synthesis of HzAla-ABT (**6**)

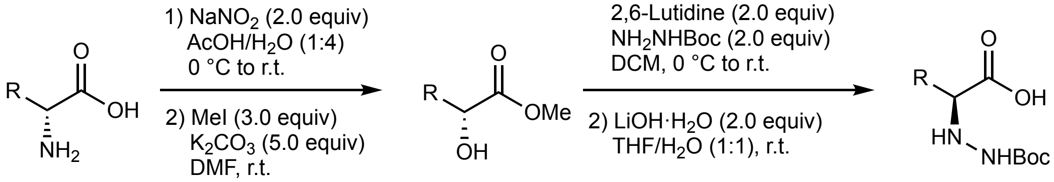

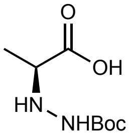

[(*tert*-Butoxycarbonyl)amino]-*L*-alanine Synthesized according to a previously reported procedure[2]; (+)-Methyl D-lactate was obtained from a commercial supplier (Sigma-Aldrich) and used as received: To a solution of (+)-methyl *D*-lactate (1.43 mL, 15.0 mmol, 1.0 equiv)

in DCM (45 mL) was added trifluoromethanesulfonic anhydride (3.28 mL, 19.5 mmol, 1.3 equiv) and 2,6-lutidine (3.47 mL, 30.0 mmol, 2.0 equiv) at 0 °C, and the reaction was stirred at the same temperature until full consumption of the starting material (confirmed by TLC). To this was then added *tert*-butyl carbazate (3.96 g, 30.0 mmol, 2.0 equiv), and the resulting mixture was further stirred at 0 °C for 4 h, then at room temperature for 16 h. The reaction mixture was diluted with DCM and washed with H_2O, brine and 1 M $HCl_{(aq)}$. The organic layer was then dried over anhydrous $MgSO_4$, concentrated under reduced pressure, and purified by flash column chromatography (30% EtOAc/*n*-Hex) to furnish [(*tert*-butoxycarbonyl)amino]-*L*-alanine methyl ester as a pale yellow oil (2.64 g, 81%).

The methyl ester (2.44 g, 11.2 mmol, 1.0 equiv) obtained above was then dissolved in 1:1 mixture of THF/H_2O (24 mL) and treated with LiOH·H_2O (940 mg, 22.4 mmol, 2.0 equiv). After stirring at room temperature for 3 h, the mixture was concentrated under reduced pressure and the remaining aqueous layer was washed with Et_2O. The aqueous layer was then acidified to pH ~1 using 1M $HCl_{(aq)}$, extracted with EtOAc, dried over anhydrous $MgSO_4$, and concentrated under reduced pressure to give [(*tert*-butoxycarbonyl)amino]-*L*-alanine as a thick colorless oil (2.08 g, 91%). Data consistent with those previously reported.

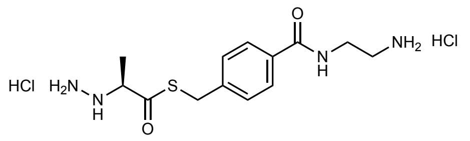

S-(4-((2-aminoethyl)carbamoyl)benzyl) (R)-2-hydrazineylpropanethioate (**6b**). Prepared according to General Procedure C using [(*tert*-butoxycarbonyl)amino]-L-alanine[2] (428 mg, 2.1 mmol), Boc-ABT (465 mg, 1.5 mmol), DMAP (512 mg, 4.2 mmol), EDC·HCl (803 mg, 4.2 mmol) and DCM (10 mL). Purification by flash column chromatography (60% EtOAc in *n*-Hex) afforded the corresponding Boc-protected product as a colorless oil (338 mg, 45%). The deprotection was achieved upon treatment with 4M solution of HCl in 1,4-dioxane, and the resulting product was used without further purification and characterization. Boc-**6b**: ^{1}H NMR (400 MHz, $CDCl_3$) δ 7.74 (d, *J* = 8.0 Hz, 2H), 7.34 (d, *J* = 8.0 Hz, 2H), 7.20 (br s, 1H), 6.26 (br s, 1H), 5.02 (br s, 1H), 4.09 (s, 2H), 3.79 (q, *J* = 7.0 Hz, 1H), 3.56 – 3.52 (m, 2H), 3.41 – 3.37 (m, 2H), 2.28 (br s, 1H), 1.44 (s, 9H), 1.42 (s, 9H), 1.31 (d, *J* = 7.0 Hz, 3H). ^{13}C NMR (101 MHz, $CDCl_3$) δ 203.1, 167.5, 157.7, 156.8, 141.4, 133.2, 129.1, 127.5, 81.3, 80.2, 66.2, 42.2, 40.1, 32.5, 28.5, 28.4, 17.3. HRMS [ESI/Q-TOF] Calculated for $C_{23}H_{36}N_4O_6S$ $[M+H]^+$: 497.2428, Found: 497.2434.

Synthesis of AOP (**7**)

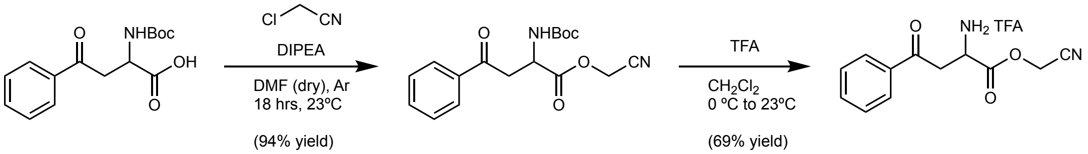

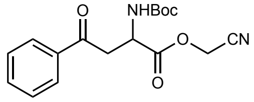

Cyanomethyl 2-((*tert*-butoxycarbonyl)amino)-4-oxo-4-phenylbutanoate (*Racemic*). Prepared according to General Procedure A. A solution of 2-((tert-butoxycarbonyl)amino)-4-oxo-4-phenylbutanoic acid (50 mg, 170 μmol, 1.00 equiv.) dissolved in 500 μL of anhydrous DMF was treated with DIPEA (148 μL, 852 μmol, 5.00 equiv.) then chloroacetonitrile (33 μL, 511 μmol, 3.00 equiv.) at 0 ºC. The reaction mixture was allowed to warm to room temperature and stirred for 18 hours. The mixture was concentrated *in vacuo* and the crude material was purified by silica gel flash chromatography (30% EtOAc/Hexanes) to afford the corresponding product as a colorless oil (53.3 g, 160 μmol, 94.1% yield) $R_f = 0.25$ (30% EtOAc/Hexanes). ^{1}H NMR (500 MHz, $CDCl_3$) δ 7.93 (d, J = 7.2 Hz, 2H), 7.60 (t, J = 7.4 Hz, 1H), 7.48 (t, J = 7.7 Hz, 2H), 5.59 (d, J = 9.0 Hz, 1H), 4.85 – 4.70 (m, 3H), 3.74 (dd, J = 18.3, 4.4 Hz, 1H), 3.58 (dd, J = 18.3, 4.0 Hz, 1H), 1.43 (s, 9H). ^{13}C NMR (126 MHz, $CDCl_3$) δ 197.57, 170.58, 155.50, 135.75, 134.13, 128.92, 128.31, 114.03, 80.57, 49.40, 49.35, 41.15, 28.35. HRMS (ESI/Q-TOF) calc. for $C_{17}H_{20}N_2O_5$ $[M + Na]^+$ = 355.1264; Found 355.1275.

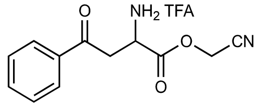

Cyanomethyl 2-amino-4-oxo-4-phenylbutanoate TFA salt (*Racemic*). **(7)** The Boc-protected amino ester (25.0 mg, 72.0 μmol) was dissolved in 5 mL of CH_2Cl_2 and treated with 0.5 mL of TFA dropwise at 0 ˚C. The solution was stirred at room temperature for 1 hour after which the volatiles were removed *in vacuo*. The off-white waxy residue was triturated with Et_2O to afford a white solid powder after filtration. (12.2 mg, 49.3 μmol, 68.5%).

^{1}H NMR (400 MHz, DMSO-d_6) δ 8.50 (s, 3H), 8.02 – 7.97 (m, 2H), 7.77 – 7.68 (m, 1H), 7.64 – 7.55 (m, 2H), 5.14 (s, 2H), 4.67 (t, J = 4.7 Hz, 1H), 3.80 (d, J = 5.0 Hz, 2H). ^{13}C NMR (126 MHz, DMSO-d_6) δ 195.75, 168.45, 135.09, 134.26, 128.98, 128.21, 115.25, 50.30, 47.64, 38.58. HRMS (ESI/Q-TOF) calc. for $C_{12}H_{12}N_2O_3$ $[M + Na]^+$ = 255.0740; Found 255.0742.

In vitro synthesis of pyridazinone

1) N-terminal incorporation

As a reporter peptide, a T7 promoter-controlled DNA template (pJL1_MT_StrepII) was designed to encode a streptavidin (Strep) tag and additional Met (AUG-X) and Thr (ACC-Y) codons (XYWHSPQFEK). The initiation codon AUG and ACC were used for N-terminal incorporation of the γ-keto and hydrazineyl ester substrates, respectively). The PURExpress™ Δ (aa, tRNA) kit (NEB, E6840S) was used for pyridazinone formation reaction and the reaction was performed with only the 8 amino acids that decode the purification tag. The reaction mixtures were incubated at 37 °C for 2 h. The synthesized peptides were then purified using Strep-Tactin®-coated magnetic beads (IBA) and characterized by MALDI-TOF mass spectrometry.

2) C-terminal incorporation (alternating consecutive incorporation)

For alternating incorporations at the C-terminal region of a peptide, the pJL1-StrepII_TI2 and pJL1-StrepII_TI3 encoding the same amino acids (MWHSPQFEKSXYXY or MWHSPQFEKSXYXYXY), where X (Thr:ACC) and Y (Ile:AUC) indicate the position of the γ-keto amino acid (**7**) and (S)-HzAla (**6**) substrates, respectively. The reaction condition, purification and characterization methods are the same with the methods described in the paragraph above.

3) Effect of other translational machinery for pyridazinone bond formation

For this study, a custom-made PURExpress® Δ (aa, tRNA, ribosome) kit (NEB, E3315Z) and the wildtype ribosome provided in the kit was not used. To investigate the engineered ribosome's effect, 15 μM (final concentration) of the engineered ribosome (Hecht's 040329)[5] was added to the reaction mixture that contains the 8 amino acids decoding the strep-tag. To investigate the EF-P's effect, additional 10 μM of EF-P[6] was added into the reaction mixture. The reaction condition, purification, and characterization methods are the same with the methods described in the paragraph above.

LC-MS analysis of pyridazinone

After 2 h at 37 °C, NaOH (5 mM in final) was added to cleave the tRNA ester linkage of 1 and 5, or the resulting pyridazinone (2-(6-oxo-3-phenyl-5,6-dihydropyridazin-1(4H)-yl)-3-phenylpropanoic acid, OPDP) from the tRNA.

Preparation of standard peptides

The standard peptides were prepared using PURExpress™ (NEB, E6800S) in the presence of the 20 natural amino acids and the pJL1_MT_StrepII plasmid that decodes the fMetThrTrpHisSerProGlnPheGluLys

peptide. The reaction mixtures were incubated at 37 °C for 2 h. The synthesized peptides were then purified using Strep-Tactin®-coated magnetic beads (IBA) and characterized by MALDI-TOF mass spectrometry.

Supplementary Figures

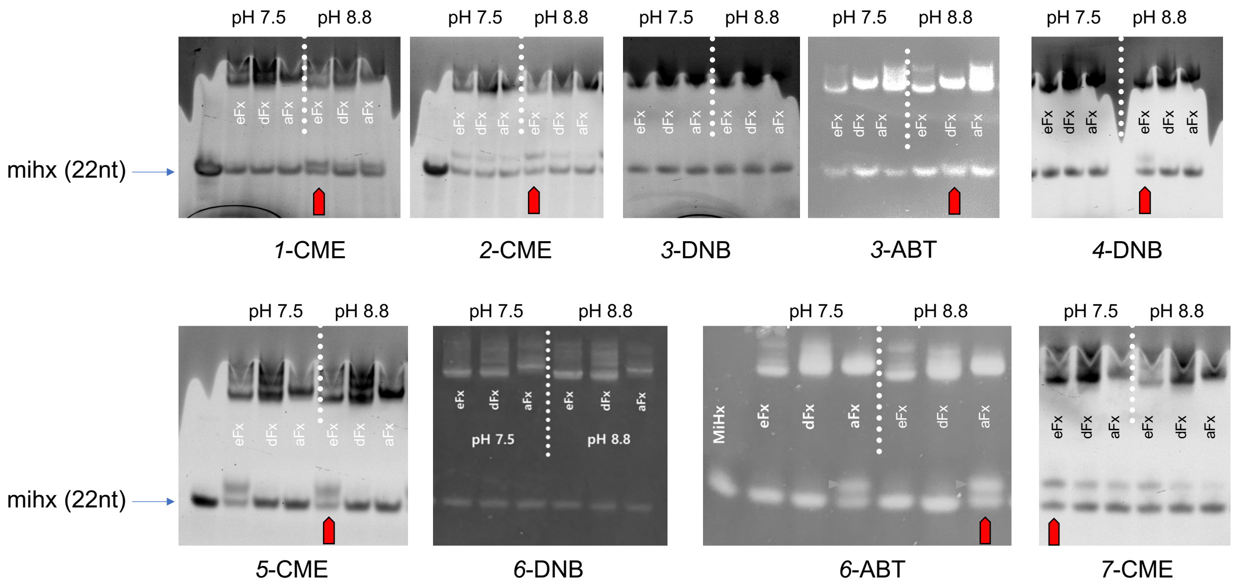

Supplementary Figure 1. Acylation of microhelix with substrates *1*-*7*. The Fx-catalyzed acylation reaction using the 7 substrates were monitored at two different pH (7.5 and 8.8) over 48 h with three different flexizymes (eFx, dFx, and aFx). The yield of each reaction was determined by quantifying the relative band intensity of unacylated and acylated microhelix (mihx) on the gel using ImageJ software. Fx: Flexizyme, mihx: microhelix. The red arrows indicate the selected acylation reaction conditions for tRNA acylation with the substrate. *3*-DNB and *6*-DNB were not charged to mihx, presumably because of poor water-solubility. Substrate structures for *1*-*7* are shown in the characterization data above. Gel data representative of three independent experiments. The samples derive from the same experiment and the densitometric analysis was performed in parallel on different gels.

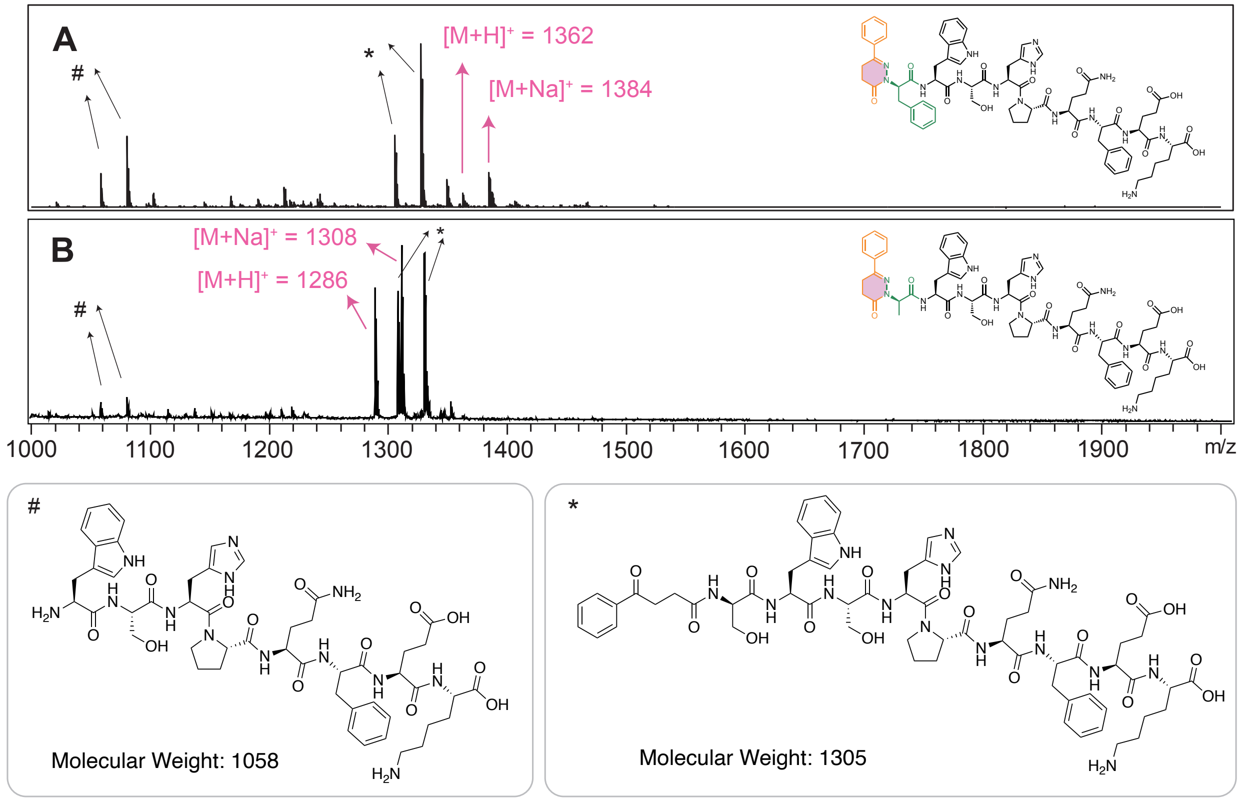

Supplementary Figure 2. Characterization of the pyridazinone products. The peaks marked as a hash (#) correspond to the theoretical mass of a truncated peptide (strep-tag reporter peptide) which does not contain both γ-keto esters and hydrazine at the N-terminus. The peaks marked as an asterisk (*) correspond to the theoretical mass of peptide that includes a Ser misincorporated at the Thr codon, resulting in a linear product rather than the formation of pyridazinone. Data representative of three independent experiments.

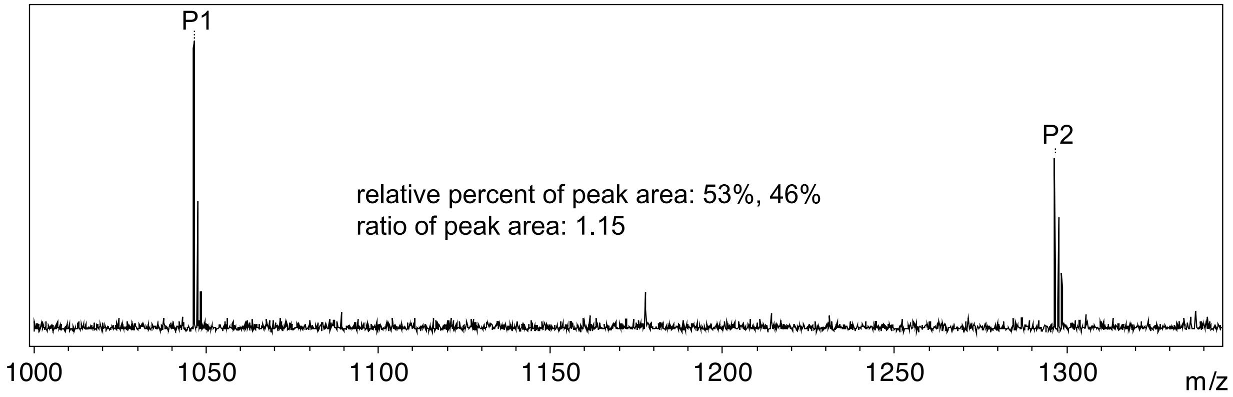

Supplementary Figure 3. The relative percent yields were determined using the peak areas. The relative percent yields of target molecules discussed in the main text were directly determined by the relative peak area corresponding to the theoretical mass (i.e., relative yield (%) = $\frac{\text{the corresponding peak area}}{\text{the sum of areas of the whole peaks shown in the mass spectrum}} \times 100$), assuming that the peptides with similar amino acid sequences would be ionized similarly and detected as much as they are present in the mixture. We chose two commercially available peptides, human angiotensin II (P1; H-DRVYIHPF-OH, MW 1046, Sigma) and angiotensin I (P2; H-DRVYIHPFHL-OH, MW 1296, Sigma), mixed them with the same molar ratio (1:1), and analyzed the peptide mixture by MALDI. The MALDI spectrum shows that the two peptides have a similar peak area ratio (P1:P2 = 1.15:1), indicating that the calibration factor is close to 1.0.

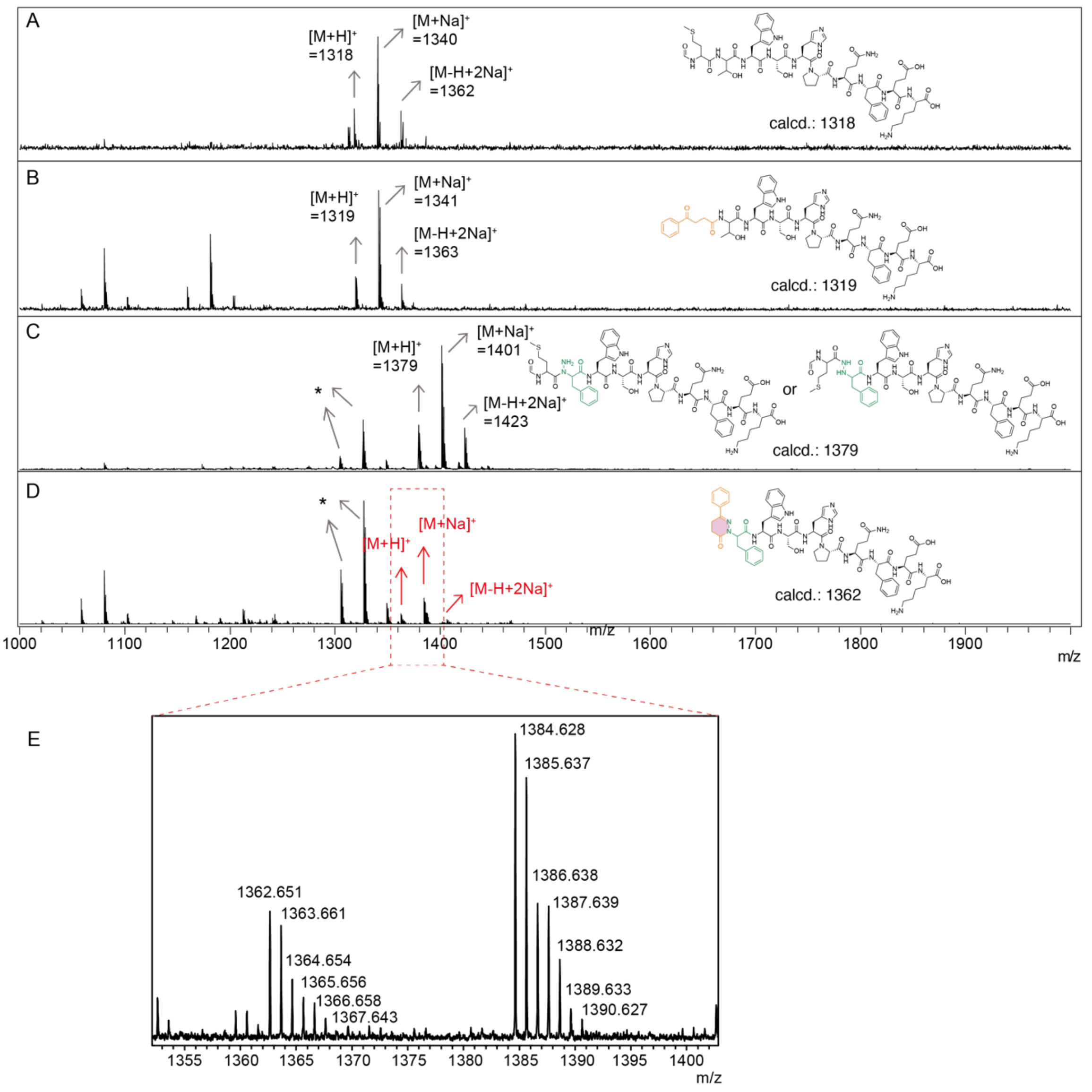

Supplementary Figure 4. Control experiments for pyridazinone bond reactions. To confirm that the pyridazinone is only formed when the tRNA^{fMet}(CAU):**1** and tRNA^{Pro1E2}(GGU):**5** complexes are supplemented into the reaction mixture, we carried out three control experiments under the same condition. (A) Mass spectrum of the peptide in the presence of all 20 natural amino acids and absence of Fx-charged tRNA. (B) Mass spectrum of the peptide produced in the presence of 9 amino acids (T + WHSPQFEK) and tRNA^{fMet}(CAU):**1** complex. (C) Mass spectrum of the peptide produced in the presence of 9 amino acids (M + WHSPQFEK) and tRNA^{Pro1E2}(GGU):**5** complex. (D) Mass spectrum of the peptide in the presence of 8 amino acids (WHSPQFEK) and tRNA^{fMet}(CAU):**1** and tRNA^{Pro1E2}(GGU):**5** complexes. (E) The enlarged mass spectrum (red box in D) shows a set of isotopic masses that increase sequentially by 1 Da from the theoretical mass. Data representative of three independent experiments.

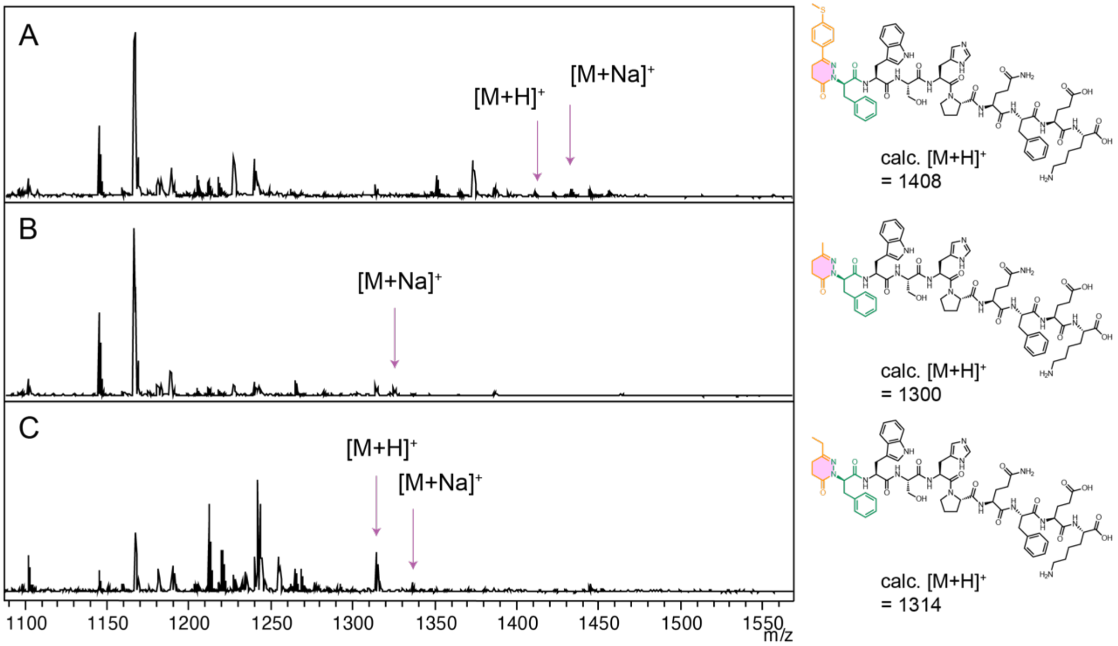

Supplementary Figure 5. Ribosome-mediated synthesis of pyridazinone derivatives with HzPhe (**5**). The pyridazinone bonds were derivatized by the use of the other γ-keto esters with HzPhe (**5**). For this, **2** and **5**, **3** and **5**, and **4** and **5** were consecutively incorporated into the AUG and ACC codon on an mRNA using $tRNA^{fMet}$(CAU) and $tRNA^{Pro1E2}$(GGU), respectively. The theoretical molecular weight and structure are shown in the right panel of of each mass spectrum. The observed masses are $[M+H]^+$ = 1409, $[M+Na]^+$ = 1431(A); $[M+Na]^+$ = 1323 (B); $[M+H]^+$ = 1314, $[M+Na]^+$ = 1336 (C). Data representative of three independent experiments.

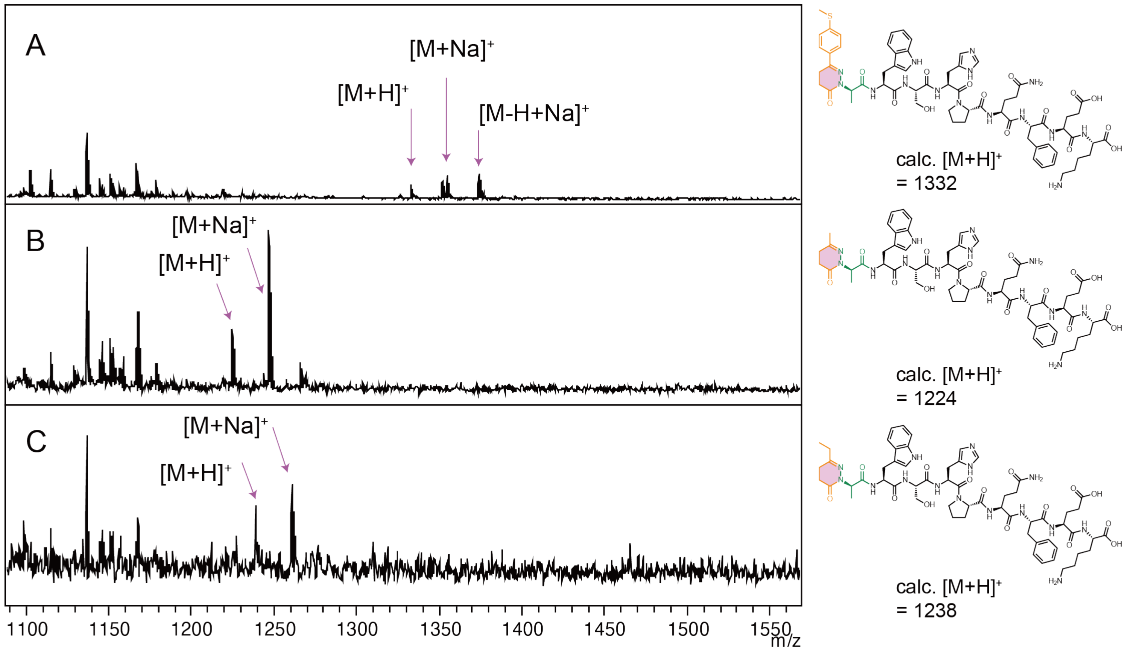

Supplementary Figure 6. Ribosome-mediated synthesis of pyridazinone derivatives with HzAla (*6*). The pyridazinone bonds were derivatized by the use of the other γ-keto esters with HzAla (*6*). For this, *2* and *6*, *3* and *6*, and *4* and *6* were consecutively incorporated into the AUG and ACC codon on an mRNA using $tRNA^{fMet}$(CAU) and $tRNA^{Pro1E2}$(GGU), respectively. The theoretical molecular weight and structure are shown in the right panel of of each mass spectrum. The observed masses are $[M+H]^+$ = 1332, $[M+Na]^+$ = 1354, $[M-H+Na]^+$ = 1376 (A); $[M+H]^+$ = 1224, $[M+Na]^+$ = 1246 (B); $[M+H]^+$ = 1238, $[M+Na]^+$ = 1260 (C). Data representative of three independent experiments.

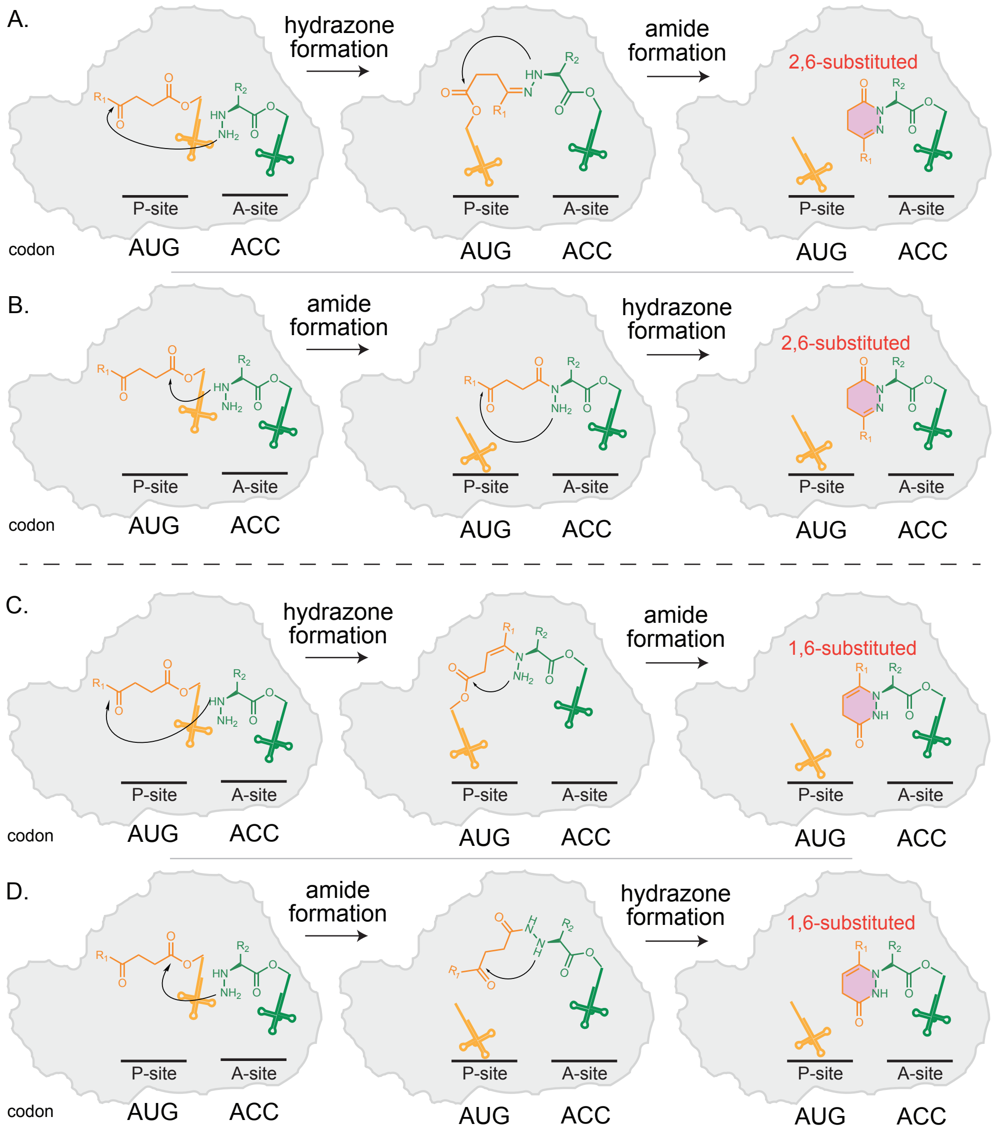

Supplementary Figure 7. Four possible mechanisms of pyridazinone bond formation in the ribosome. (A) The β-nitrogen atom of hydrazineyl ester (green) coming into the A-site of the ribosome attacks the carbonyl of the ketone of γ-keto ester (orange) to form an imine followed by removal of water. Next, the α-nitrogen atom attacks the ester bond to tRNA, thereby resulting in the formation of 2,6-substituted pyridazinone bond. (B) The α-nitrogen atom forms a hydrazone with ketone and then β-nitrogen atom forms a hydrazone, resulting in production of a 2,6-substituted pyridazinone bond. (C) With the similar mechanism, when the α-nitrogen atom of hydrazineyl ester forms hydrazone first and the β-nitrogen atom forms a peptide bond, 1,6-substituted pyridazinone is produced. (D) The β-nitrogen atom forms an amide and then the α-nitrogen atom forms a hydrazone with ketone, resulting in production of a 1,6-substituted pyridazinone.

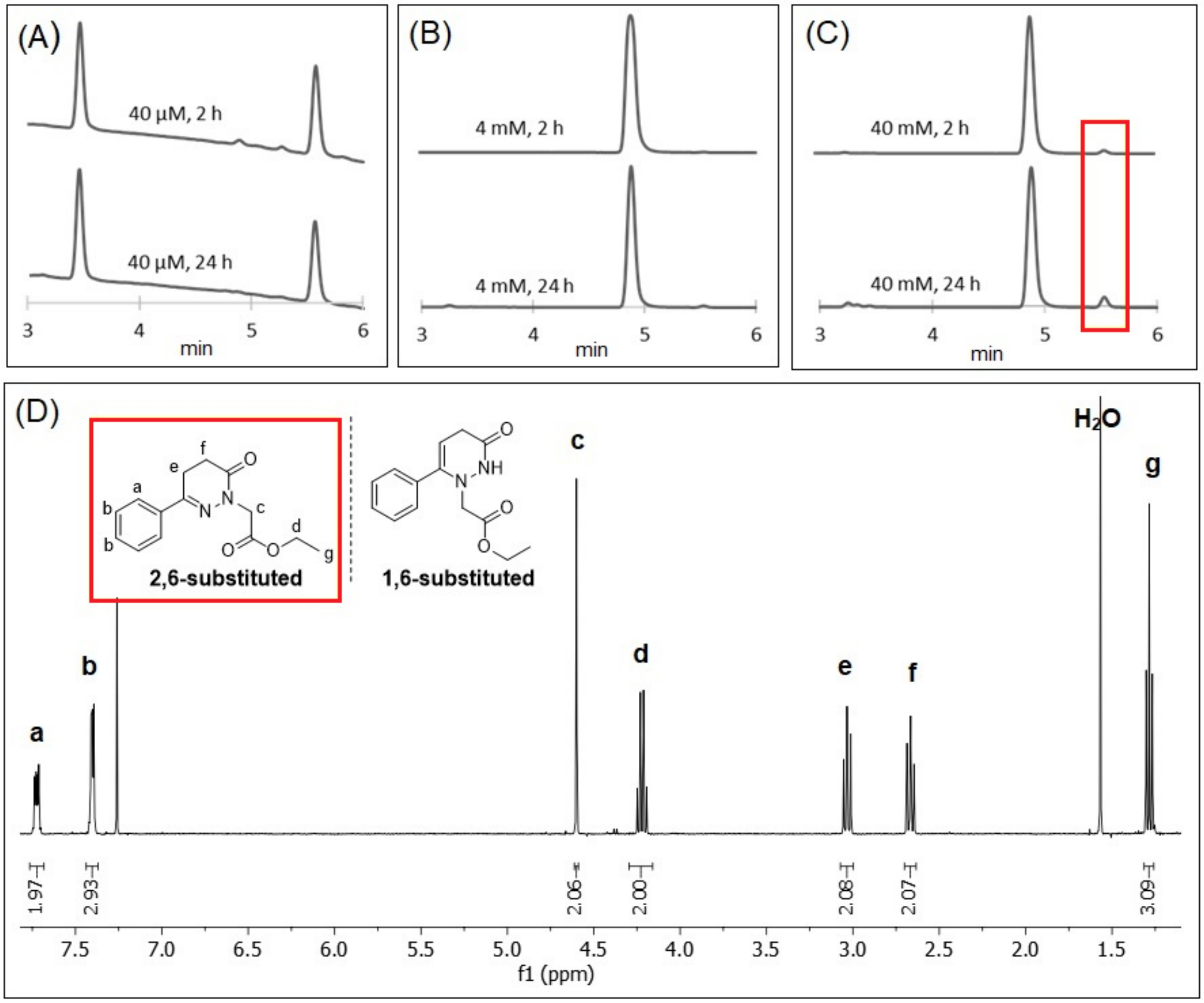

Supplementary Figure 8. Monitoring the pyridazinone formation reaction. The pyridazinone was produced in the reaction of cyanomethyl 4-oxo-4-phenylbutanoate with ethyl hydrazinoacetate hydrochloride in EtOH/H_2O (3/2: v/v) at 37 °C. The reactions were monitored by LC-MS with different substrate concentrations of 40 µM (A), 4 mM (B), and 40 mM (C). The pyridazinone product was only observed when the concentration of reactants was 1,000 times higher than that used in the ribosome-mediated reaction. The fraction collected at the 5.5 min peak (red box in panel C) was confirmed to be a 2,6-substituted pyridazinone (D) through ^{1}H NMR spectroscopy (400 MHz, $CDCl_3$). Data representative of three independent experiments.

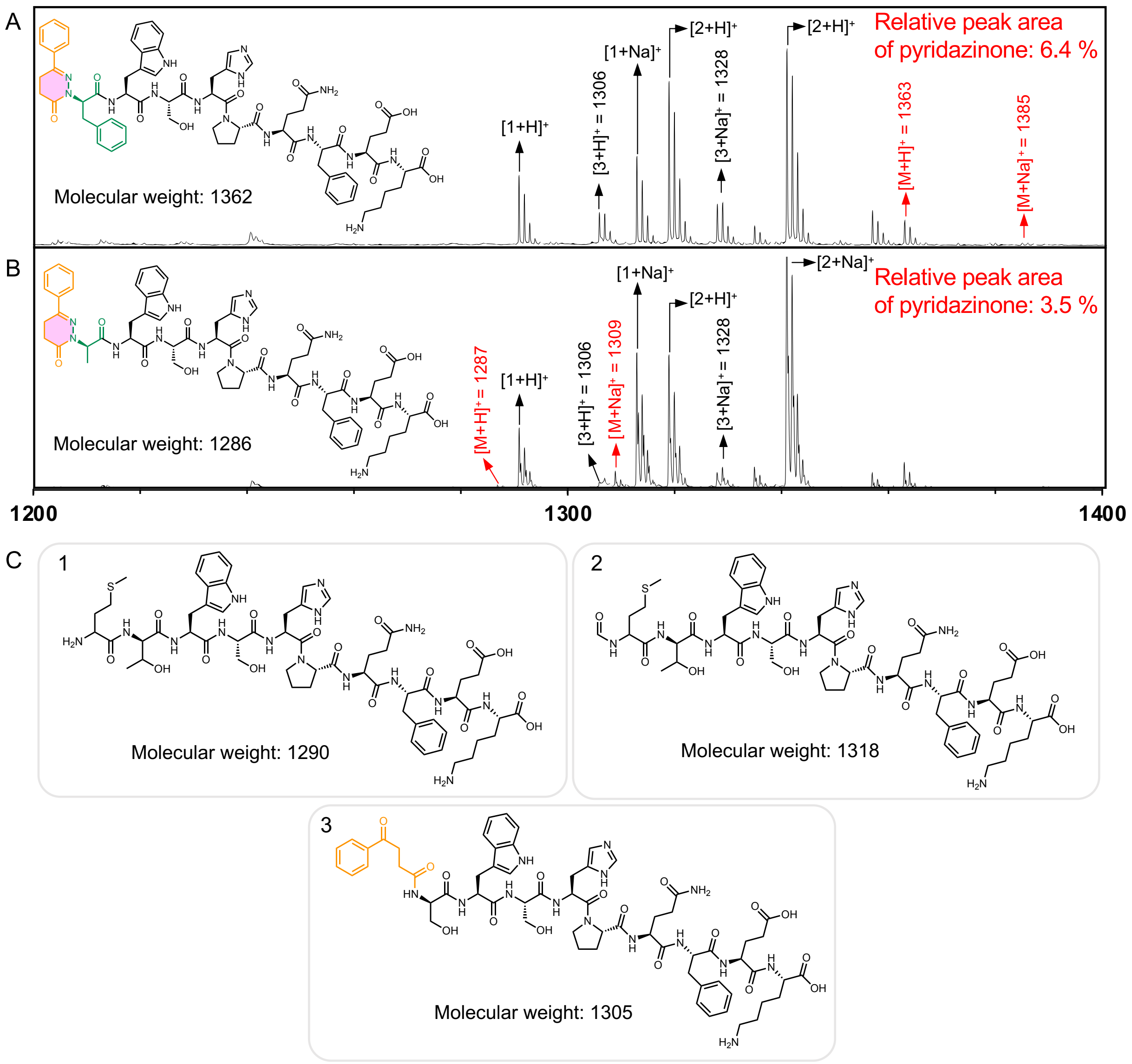

Supplementary Figure 9. Estimated yields of pyridazinone-based peptides. We separately prepared an internal standard peptide (fMetThrTrpSerHisProGlnPheGluLys) using the same plasmid and the natural 20 amino acids in the PURExpress™. We produced ~1 µg of the standard peptide (PURExpress™ system produces ~100ng per µL of reaction volume)[7], mixed 100 ng of the peptide standard with each pyridazinone peptide (panel A or B) and analyzed the mixture by MALDI-TOF. After characterization of each peak using the information on panel C, we calculated the relative peak area of the pyridazinone peptides formed between substrates **1** and **5** or **1** and **6**. The relative peak area of each peptide containing a pyridazinone is 6.4 and 3.5 %, which indicates ~60 and 30 ng of the target peptides is obtained in a 10 µL reaction. Data representative of three independent experiments.

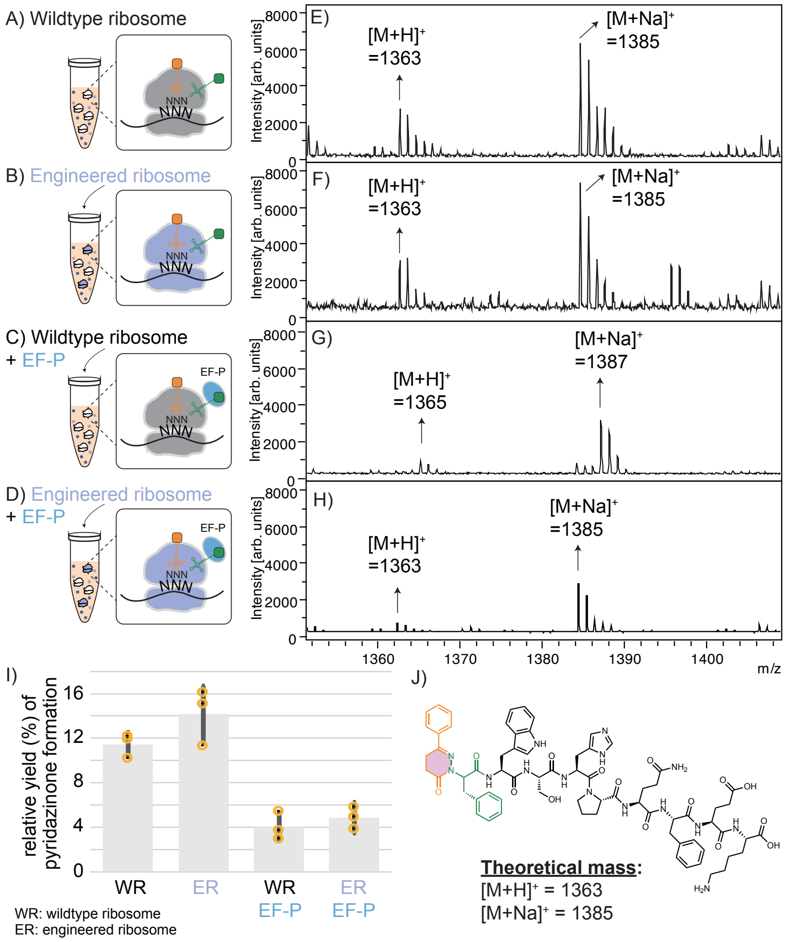

Supplementary Figure 10. Effect of engineered translational machinery on the pyridazinone bond formation with *1* and *5*. γKPhe (*1*) and (S)-HzPhe (*5*) were charged to tRNA^{fMet}(CAU) and tRNA^{Pro1E2}(GGU), respectively by Fx and subsequently added to the PURExpress^{TM} system. The *1* and *5* delivered to the ribosome on tRNA^{fMet}(CAU) and tRNA^{Pro1E2}(GGU), respectively, were consecutively incorporated into a peptide polymer and permitted to undergo water condensation reactions, yielding a pyridazinone bond. (A-B) The pyridazinone bond was produced either in the presence of the wild-type and an engineered ribosome. (C-D) Peptides containing a pyridazinone at the N-terminus were observed in a low yield when an additional translational machinery, EF-P, was supplemented. Of note, a custom-made Δ (aa, tRNA, ribosome) PURExpress system (NEB, E3315Z) supplying the wild-type ribosome in a separate tube was used for the condensation reactions with engineered ribosomes. The wild-type ribosome supplied in the kit was not used, however, based on previous literature, we expected the engineered 040329 ribosomes to constitute ~25% of the purified ribosome population. (E-H) The observed mass of each peptide corresponds to the theoretical mass, which is $[M+H]^+ = 1363$; $[M+Na]^+ = 1385$ (see panel J for the structure of theoretical peptide). Data in E, F, G, and H representative of three independent experiments. (I) The percent yield of ribosome-mediated condensation of pyridazinone bond. The yield was obtained based on the relative peak area of the peptide containing a pyridazinone bond shown in the MALDI spectra. The measure of center is a mean value and the error bars represent the standard deviation of n = 3 independent experiments. Each data point is shown in orange. (J) Structure of the peptide containing a pyridazinone bond of the N-terminus. (percent yield = the peak area corresponding to the theoretical mass / the sum of areas of the whole peaks shown in the mass spectrum). arb. units: arbitrary units

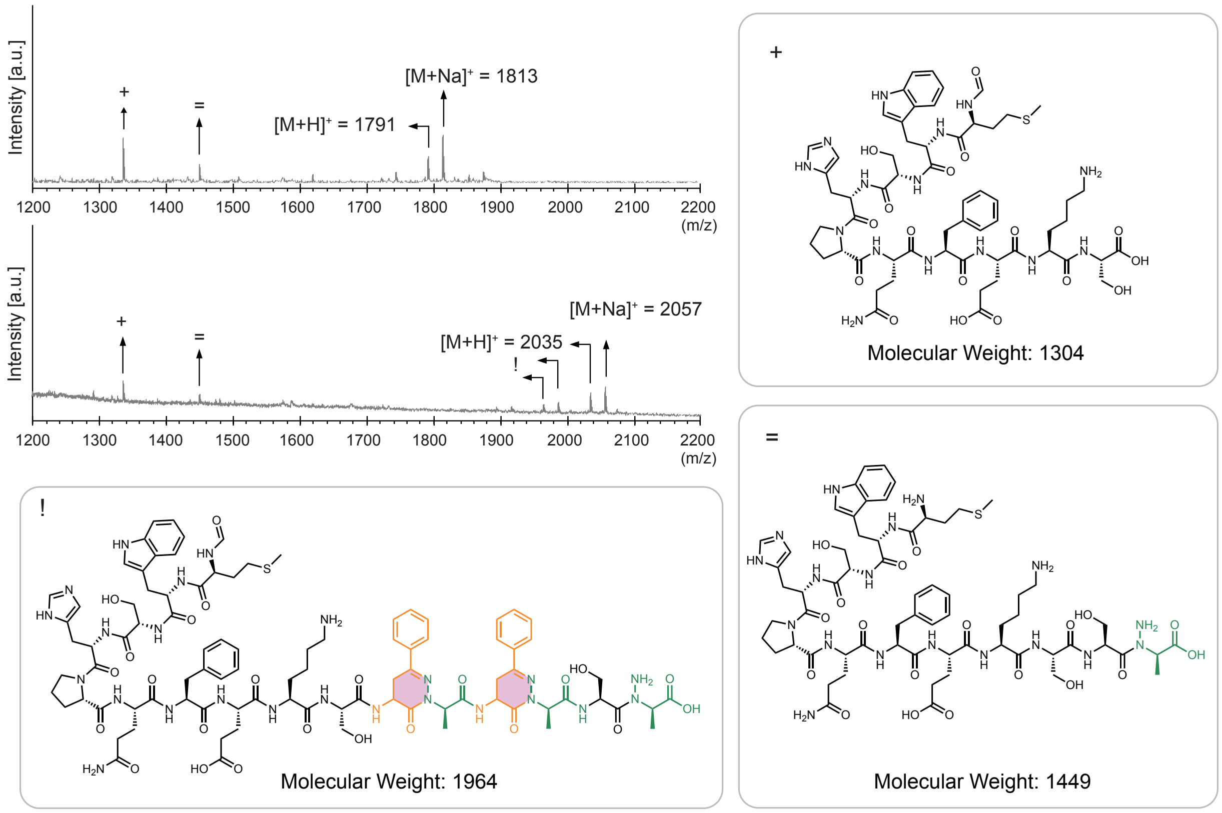

Supplementary Figure 11. Characterization of the alternating oligomers with a pyridazinone backbone. The peaks marked as a plus (+), equal (=), and exclamation (!) symbol in the mass spectra used in Figure 4B correspond to the theoretical mass of peptides that are truncated or not fully cyclized due to the misincorporation of Ser at the Thr codon. (+): $[M+H]^+ = 1304$, (=): $[M+H]^+ = 1449$, (!): ($[M+H]^+ = 1964$, $[M+Na]^+ = 1986$). Data representative of three independent experiments.

^{1}H and ^{13}C NMR Spectra

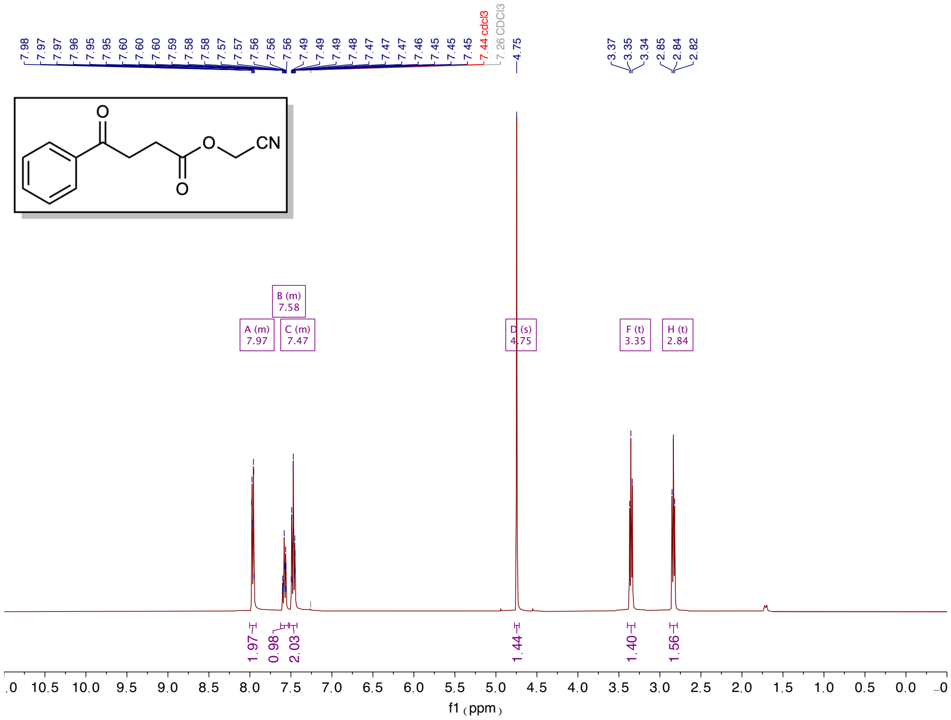

Supplementary Figure 12. ^{1}H NMR (400 MHz, $CDCl_3$) of **1**.

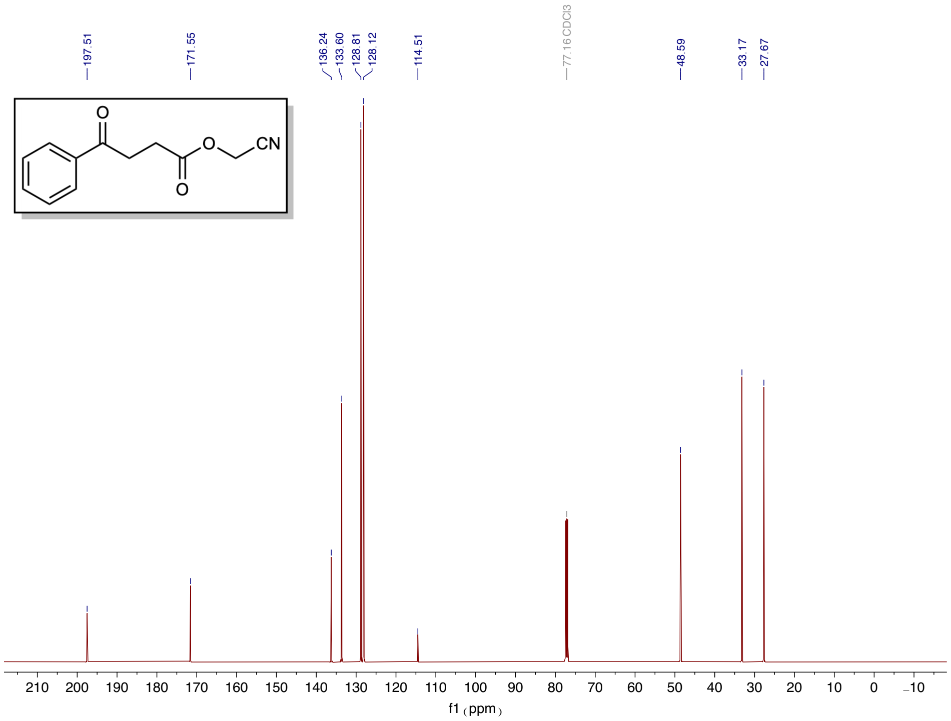

Supplementary Figure 13. ^{13}C NMR (126 MHz, $CDCl_3$) of **1**.

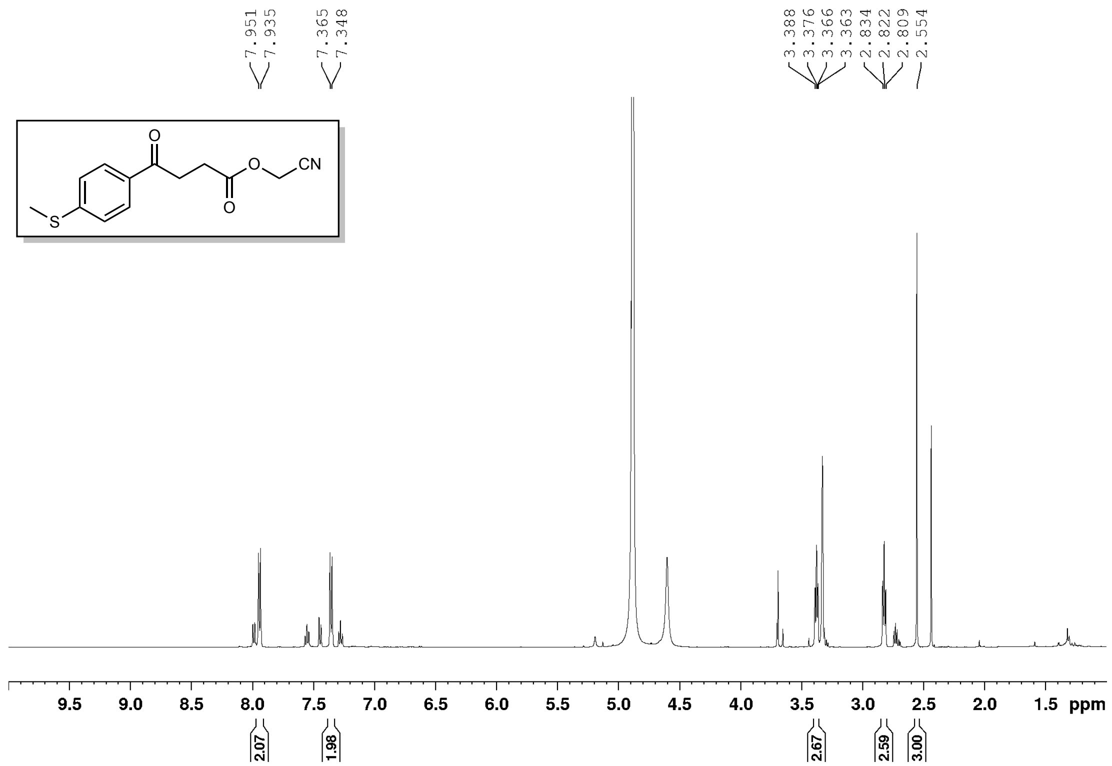

Supplementary Figure 14. ^{1}H NMR (500 MHz, CD_3OD) of **2**.

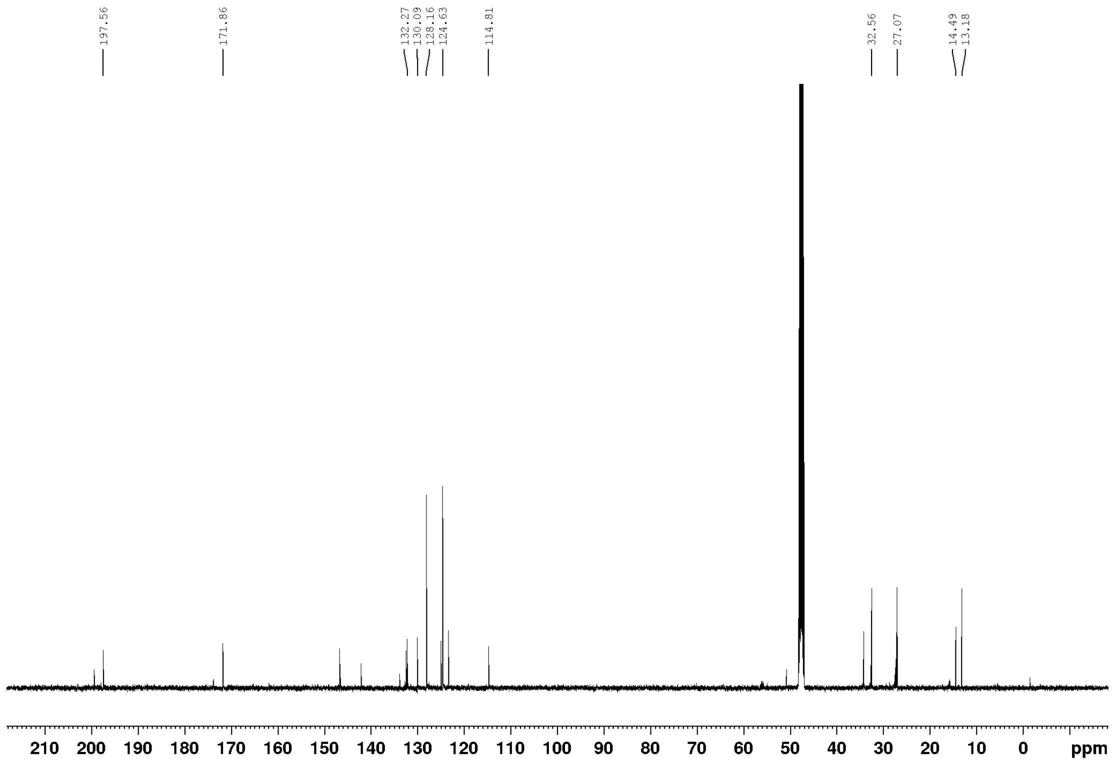

Supplementary Figure 15. ^{13}C NMR (125 MHz, CD_3OD) of **2**.

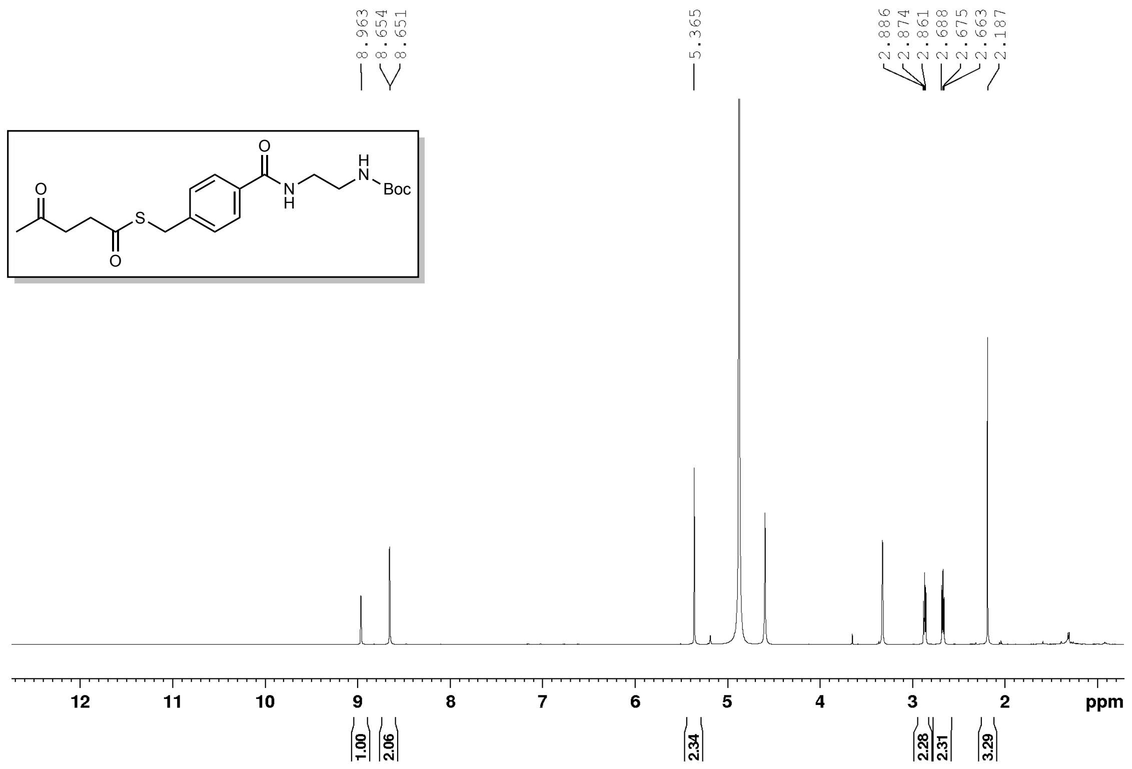

Supplementary Figure 16. ^{1}H NMR (500 MHz, CD_3OD) of **3a**.

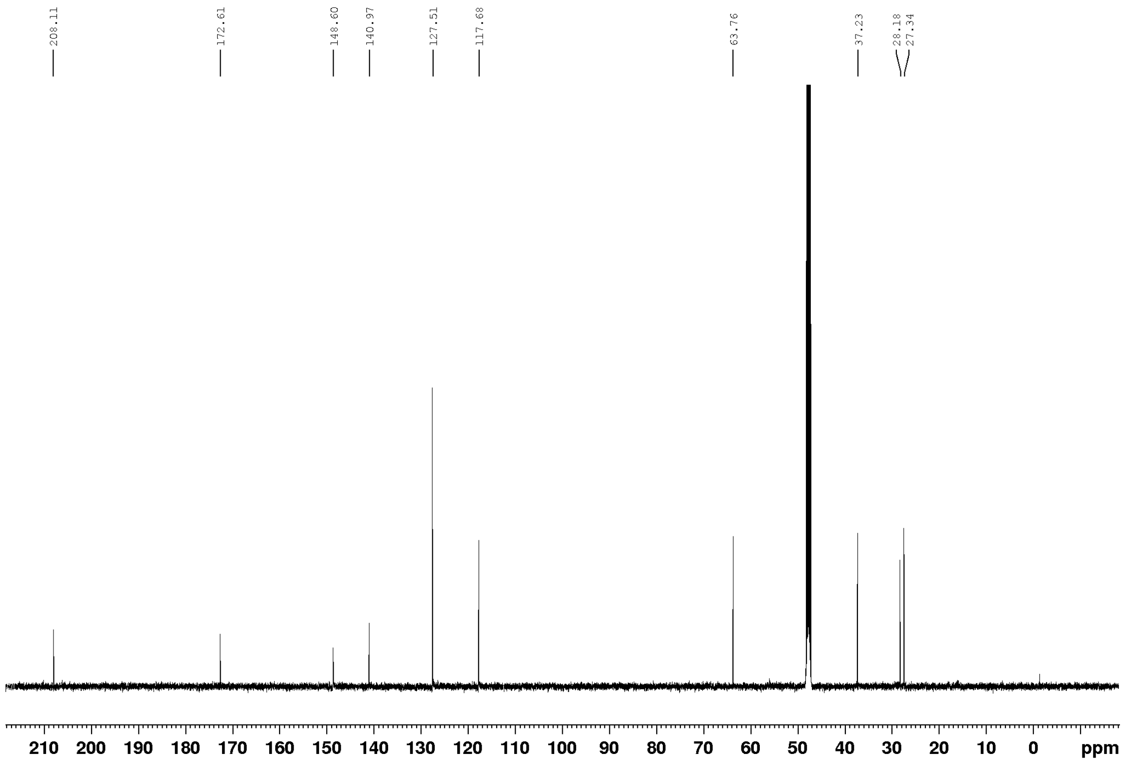

Supplementary Figure 17. ^{13}C NMR (125 MHz, CD_3OD) of **3a**.

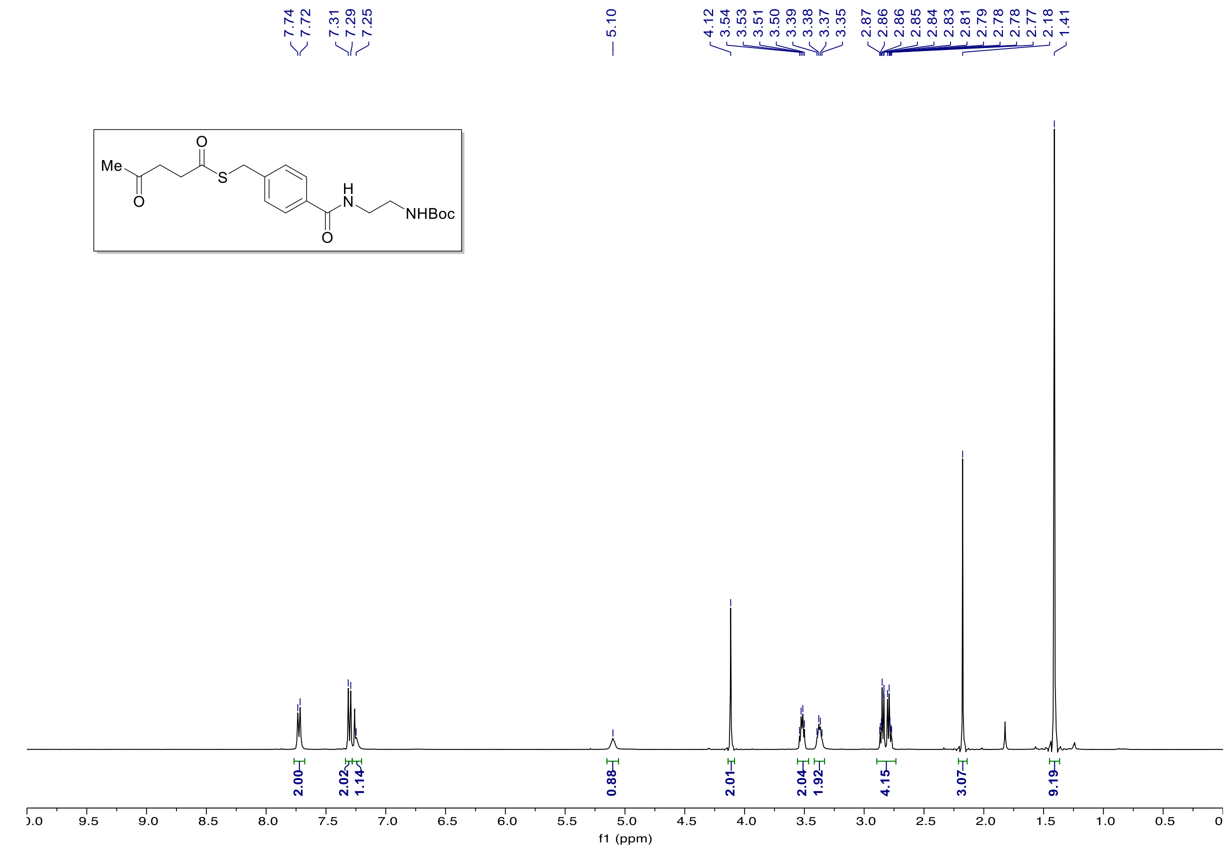

Supplementary Figure 18. ^{1}H NMR (400 MHz, $CDCl_3$) of **3b**.

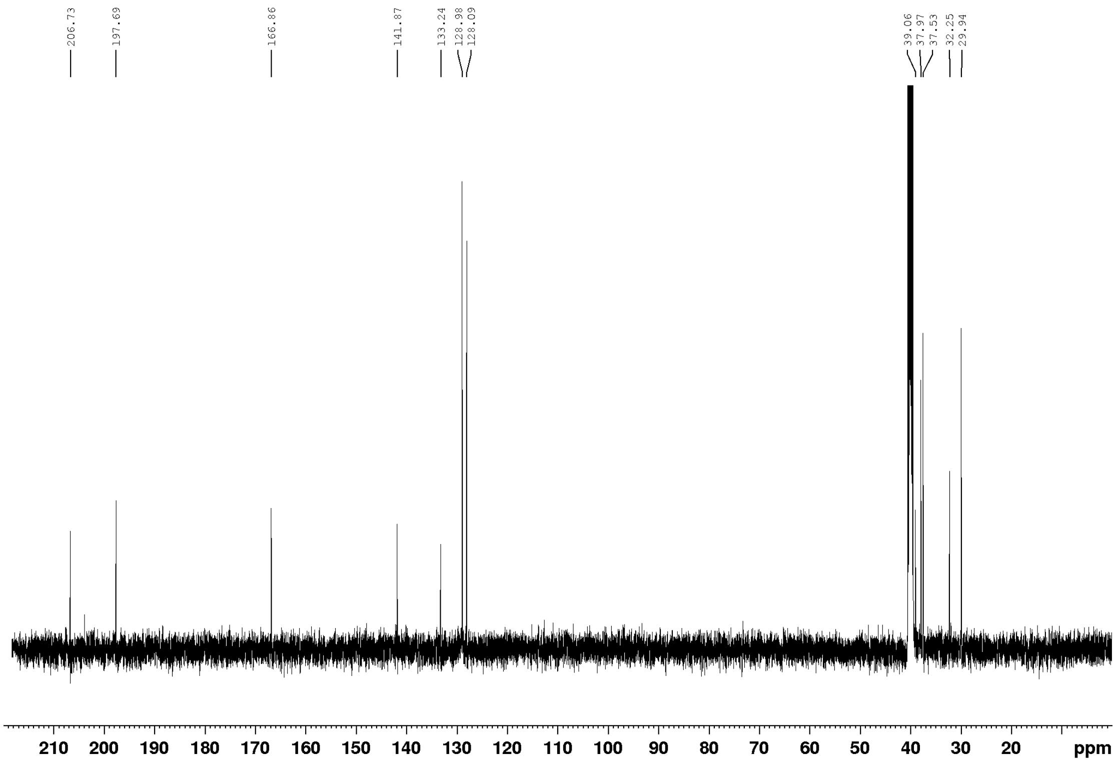

Supplementary Figure 19. ^{13}C NMR (125 MHz, DMSO-d6) of **3b**.

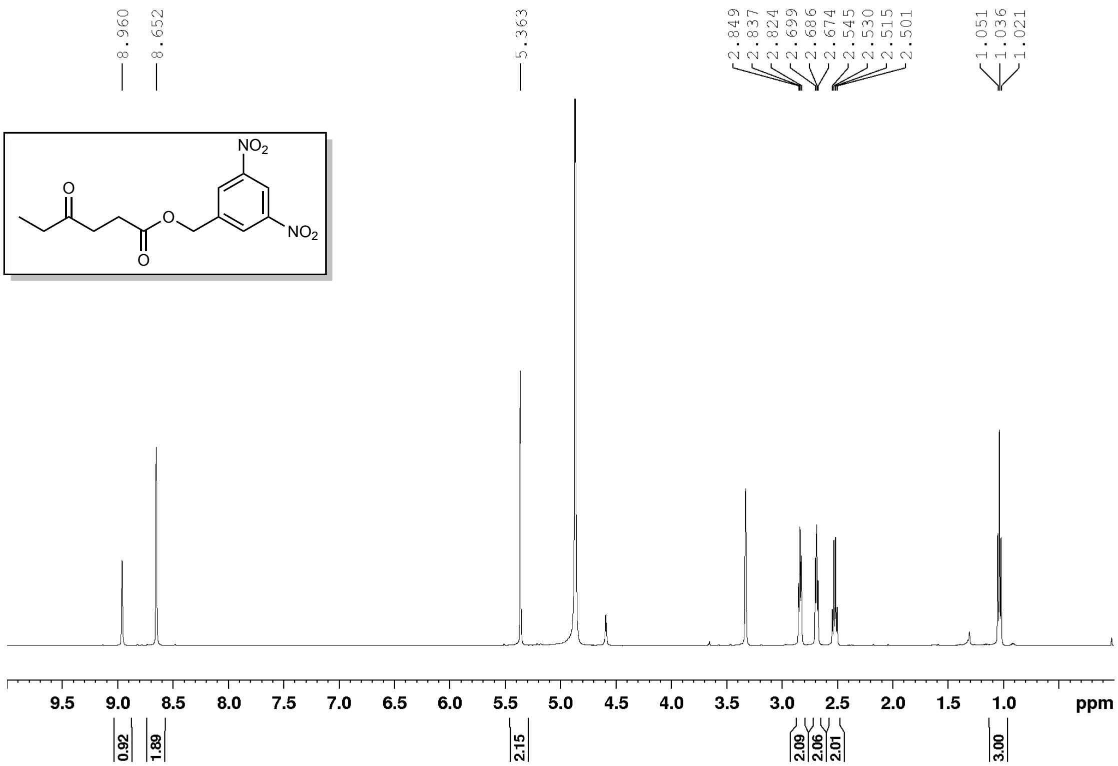

Supplementary Figure 20. ^{1}H NMR (500 MHz, CD_3OD) of **4**.

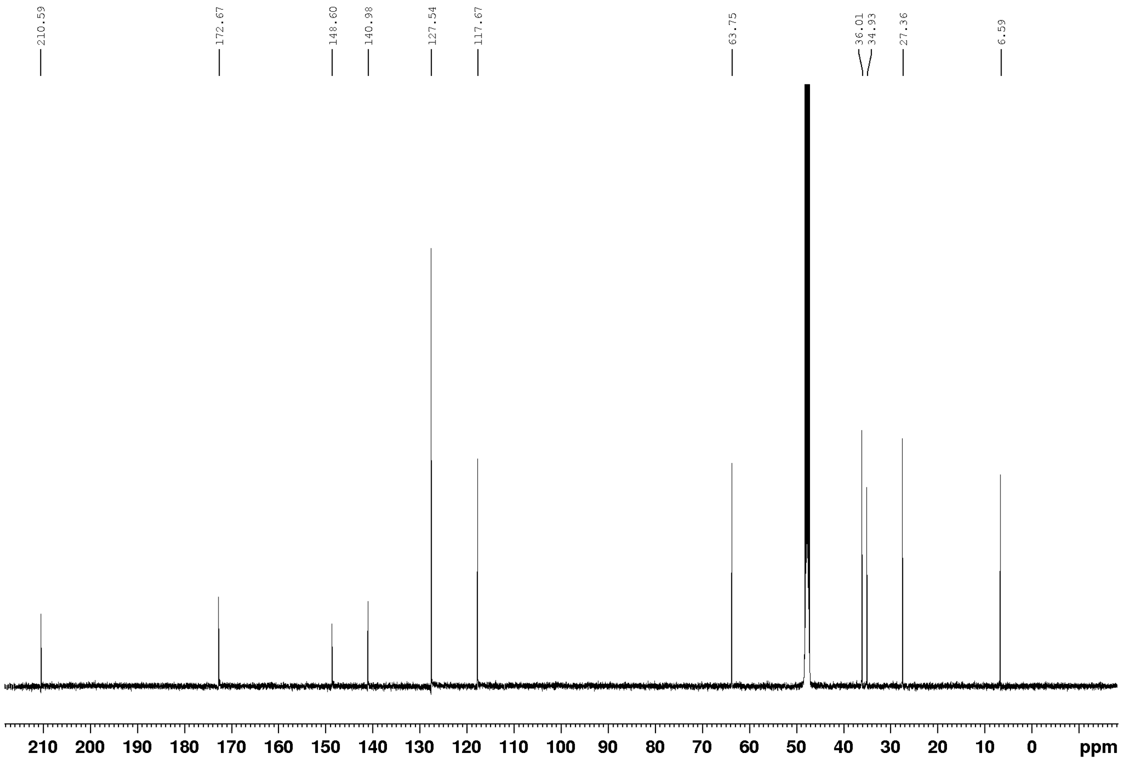

Supplementary Figure 21. ^{13}C NMR (125 MHz, CD_3OD) of **4.**

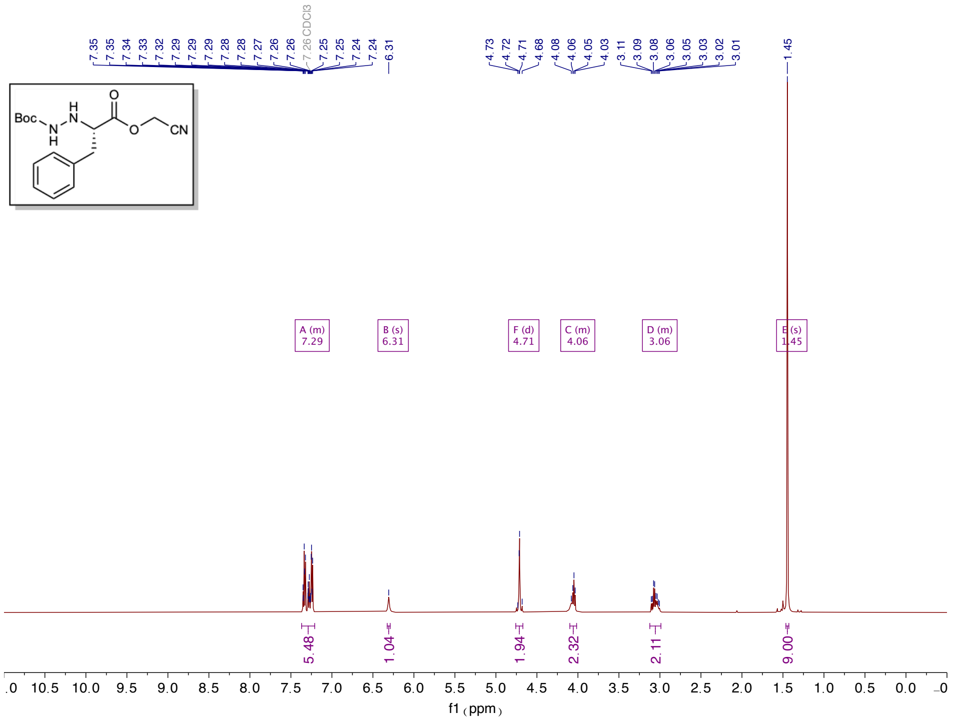

Supplementary Figure 22. ^{1}H NMR (500 MHz, $CDCl_3$) of Boc-**5**.

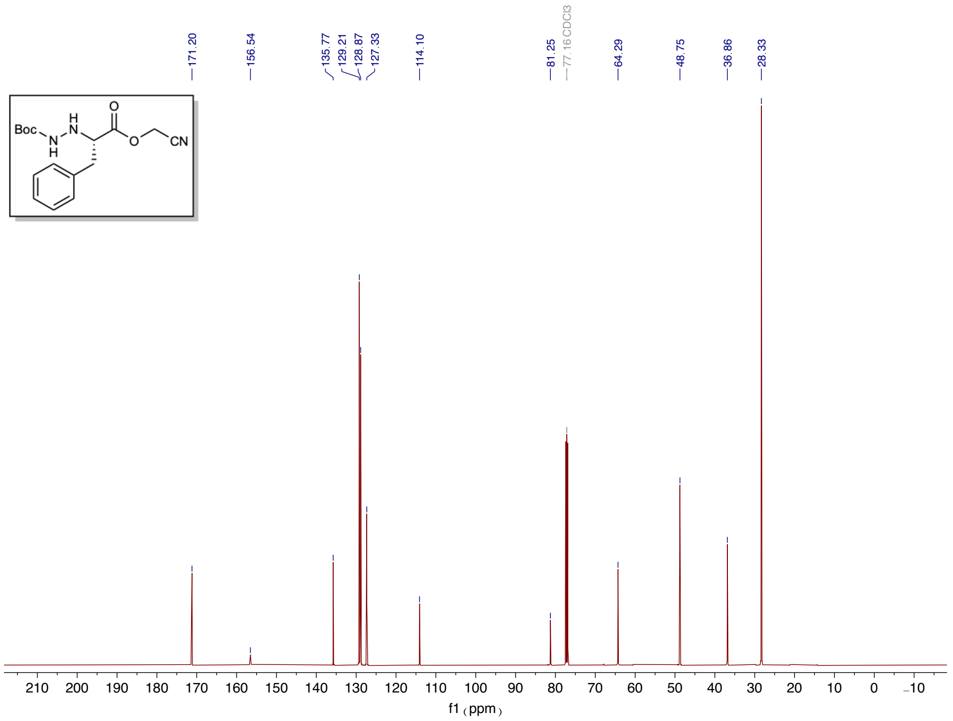

Supplementary Figure 23. ^{13}C NMR (126 MHz, $CDCl_3$) of Boc-**5.**

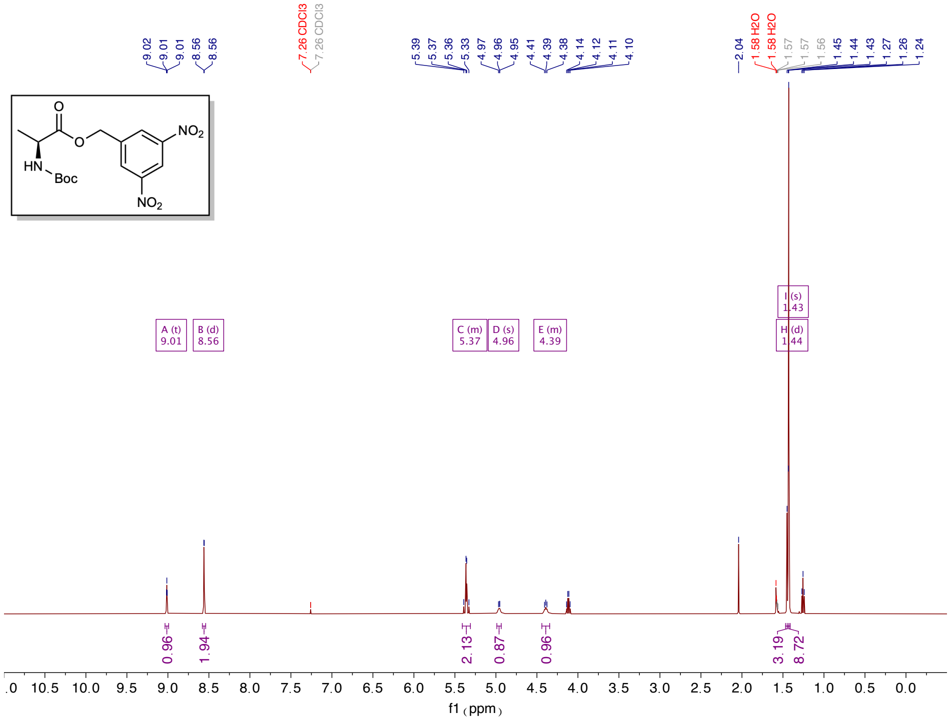

Supplementary Figure 24. ^{1}H NMR (500 MHz, $CDCl_3$) of Boc-Ala-DNB.

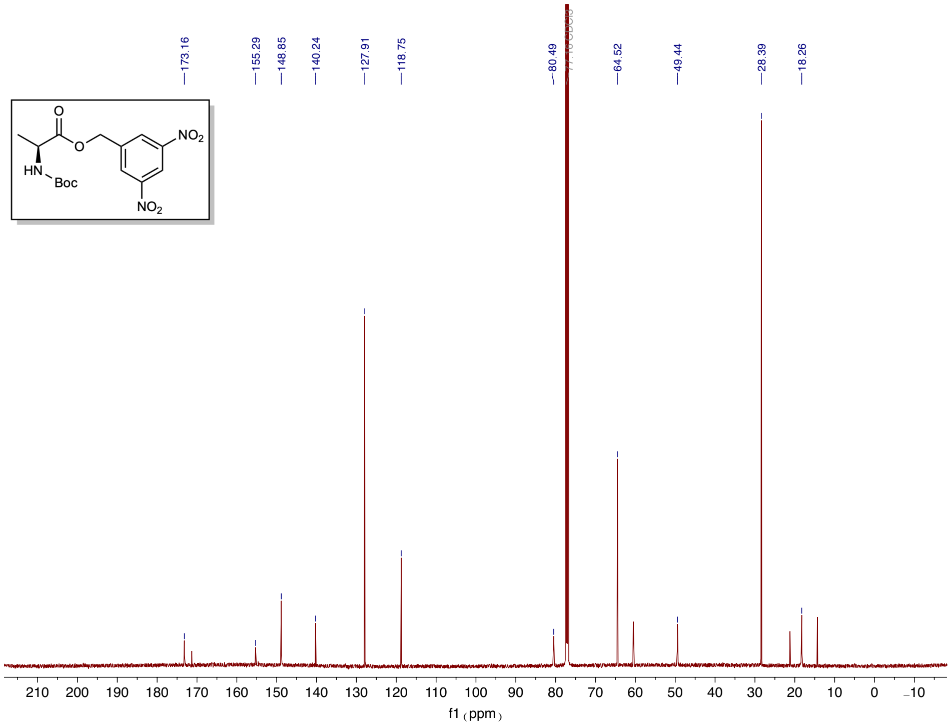

Supplementary Figure 25. ^{13}C NMR (126 MHz, $CDCl_3$) of Boc-Ala-DNB.

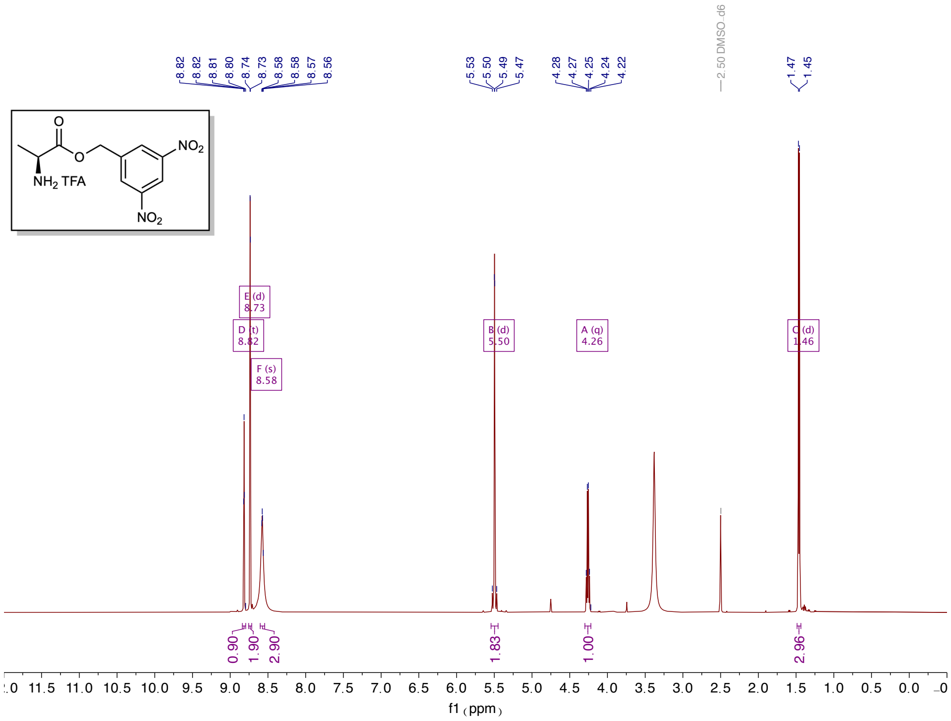

Supplementary Figure 26. ^{1}H NMR (500 MHz, DMSO-d_6) of Ala-DNB.

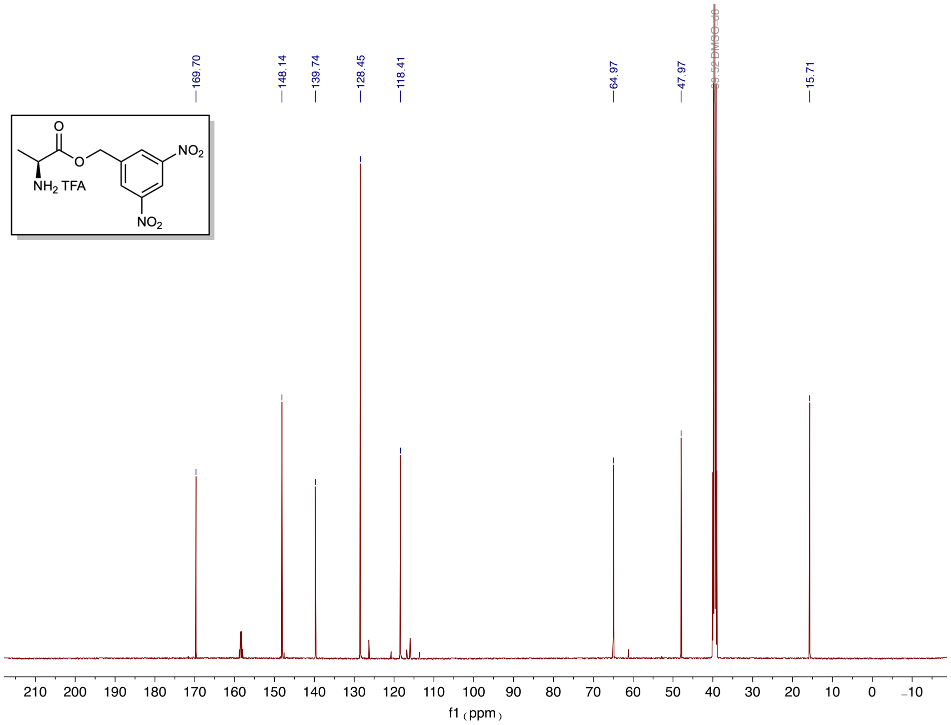

Supplementary Figure 27. ^{13}C NMR (126 MHz, DMSO-d_6) of Ala-DNB.

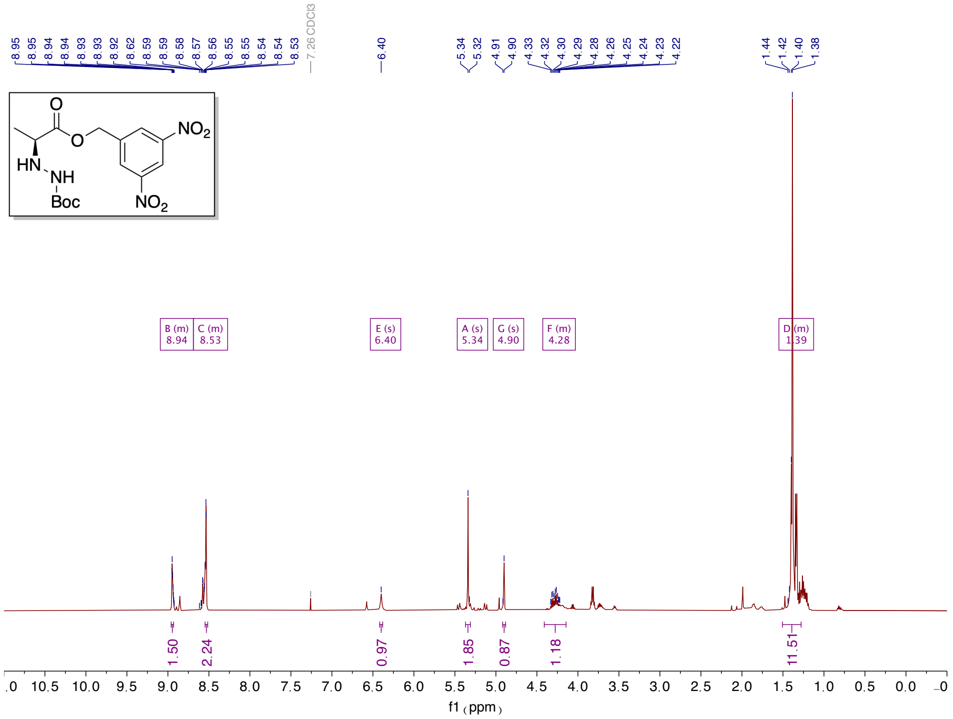

Supplementary Figure 28. ^{1}H NMR (500 MHz, $CDCl_3$) of Boc-**6a**.

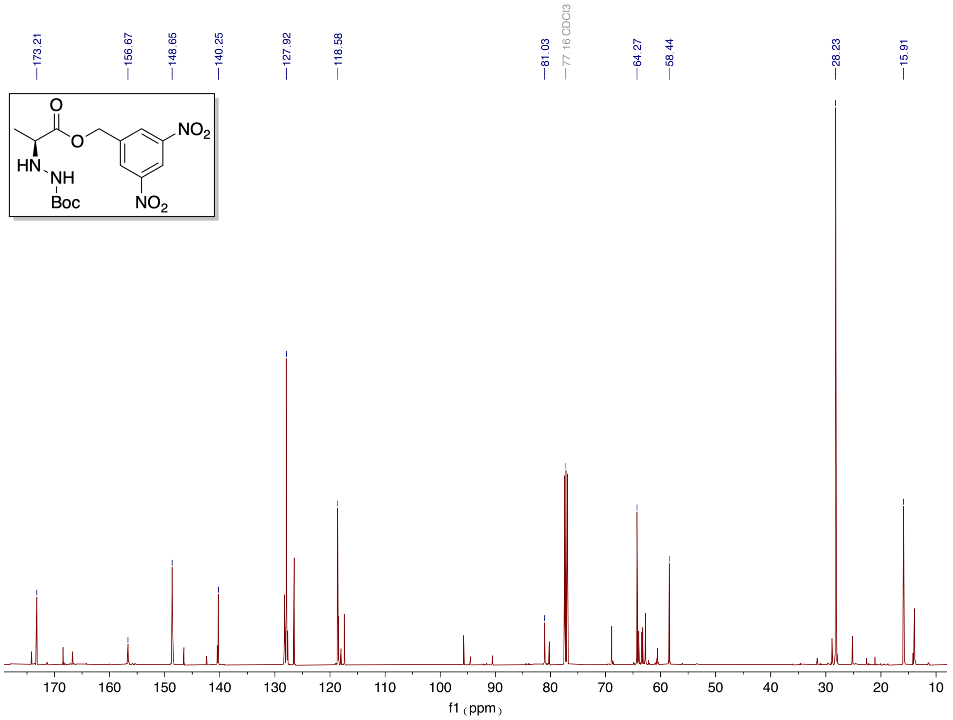

Supplementary Figure 29. ^{13}C NMR (126 MHz, $CDCl_3$) of Boc-**6a**.

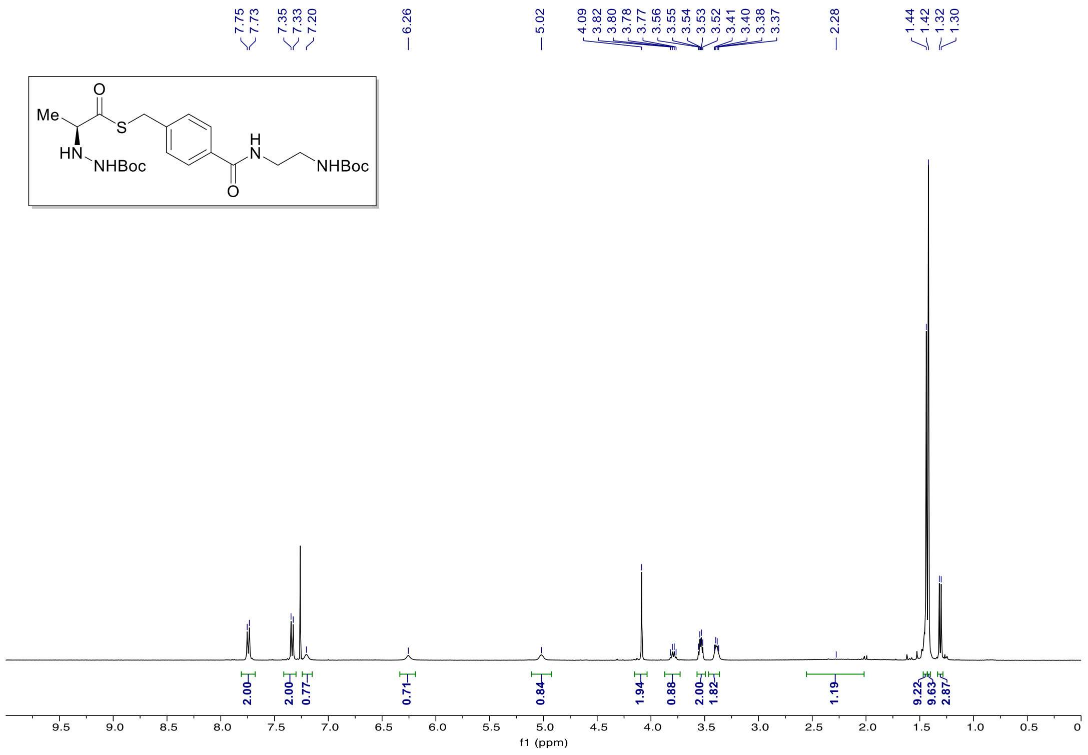

Supplementary Figure 30. ^{1}H NMR (400 MHz, CD_3OD) of **6b**.

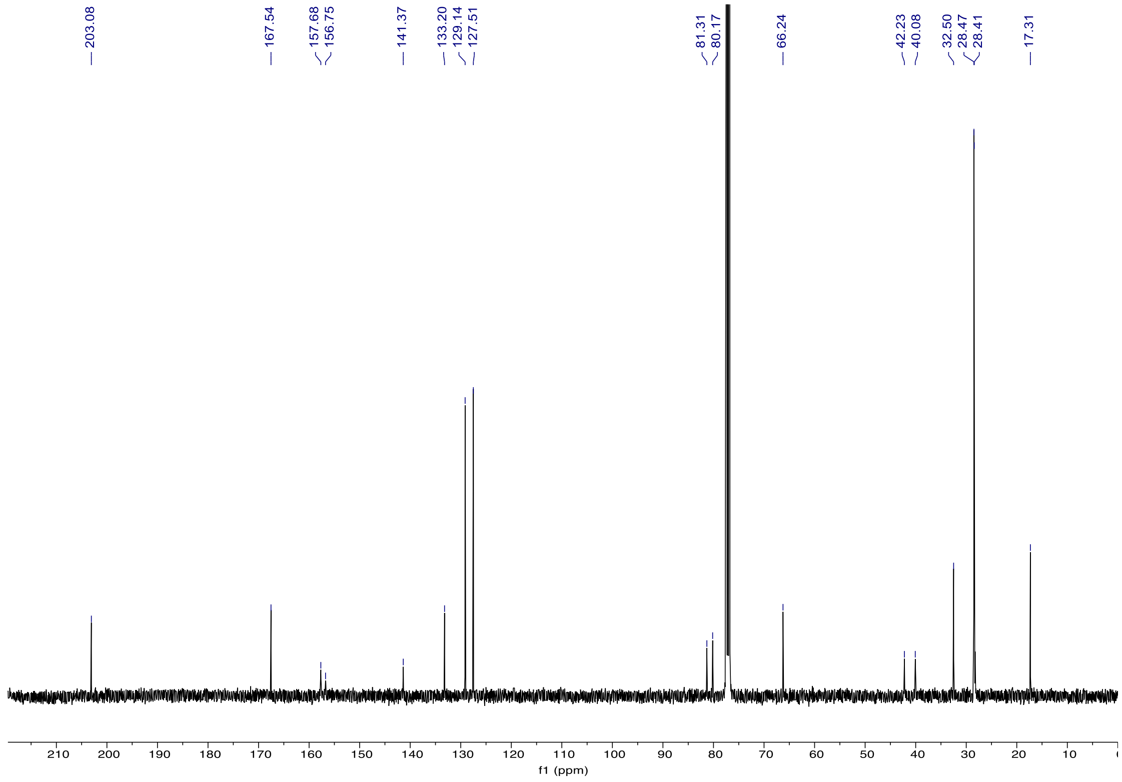

Supplementary Figure 31. ^{13}C NMR (125 MHz, DMSO-d6) of **6b**.

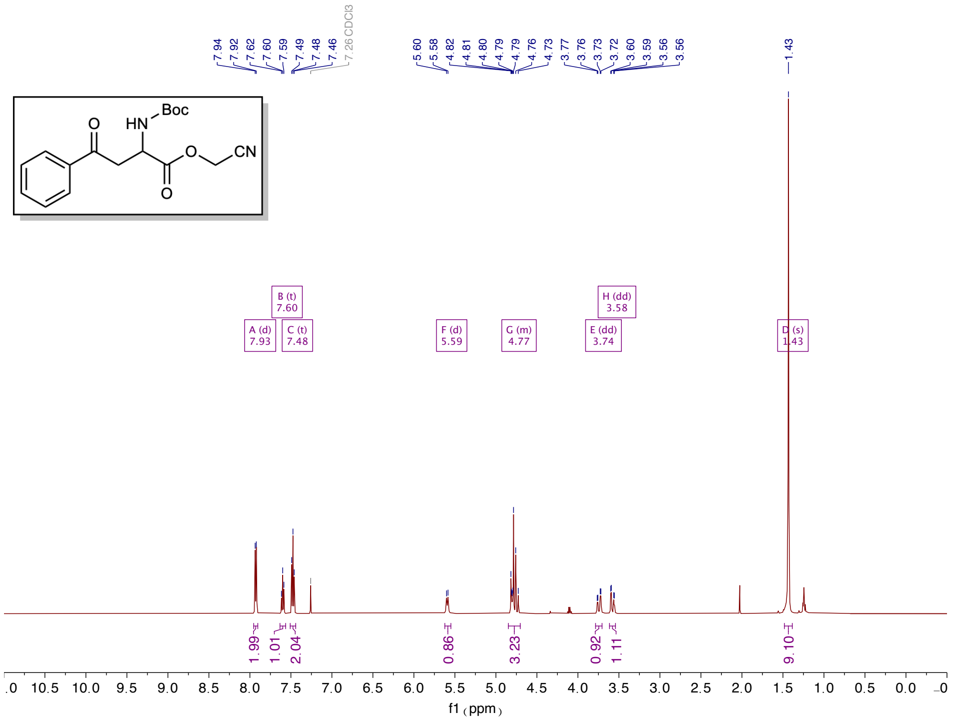

Supplementary Figure 32. ^{1}H NMR (500 MHz, $CDCl_3$) of Boc-**7**.

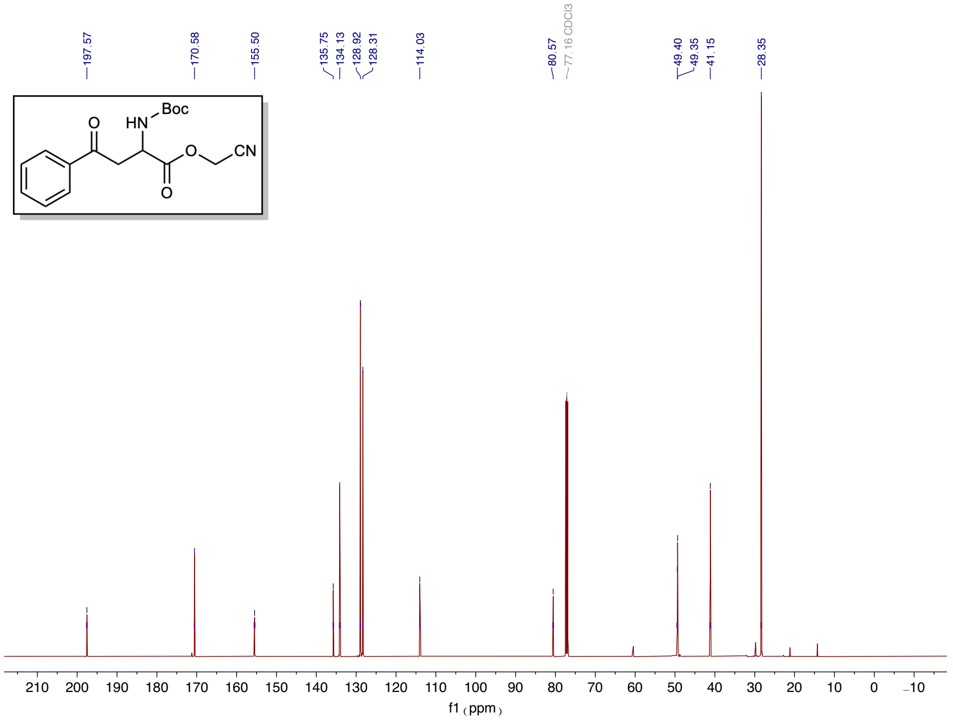

Supplementary Figure 33. ^{13}C NMR (126 MHz, $CDCl_3$) of Boc-**7**.

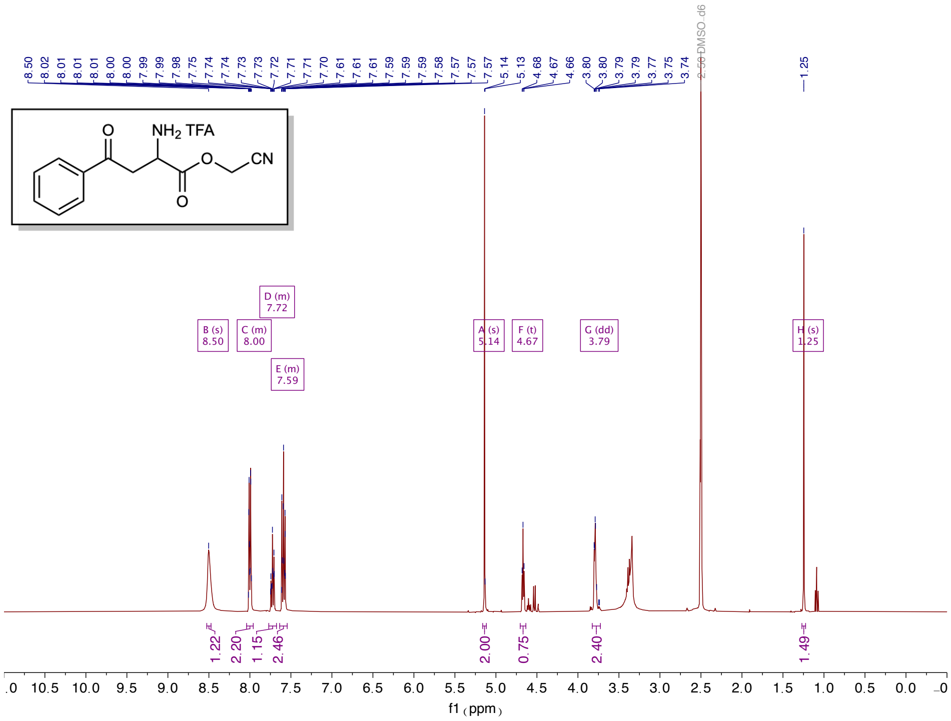

Supplementary Figure 34. ^{1}H NMR (400 MHz, DMSO-d6) of **7**.

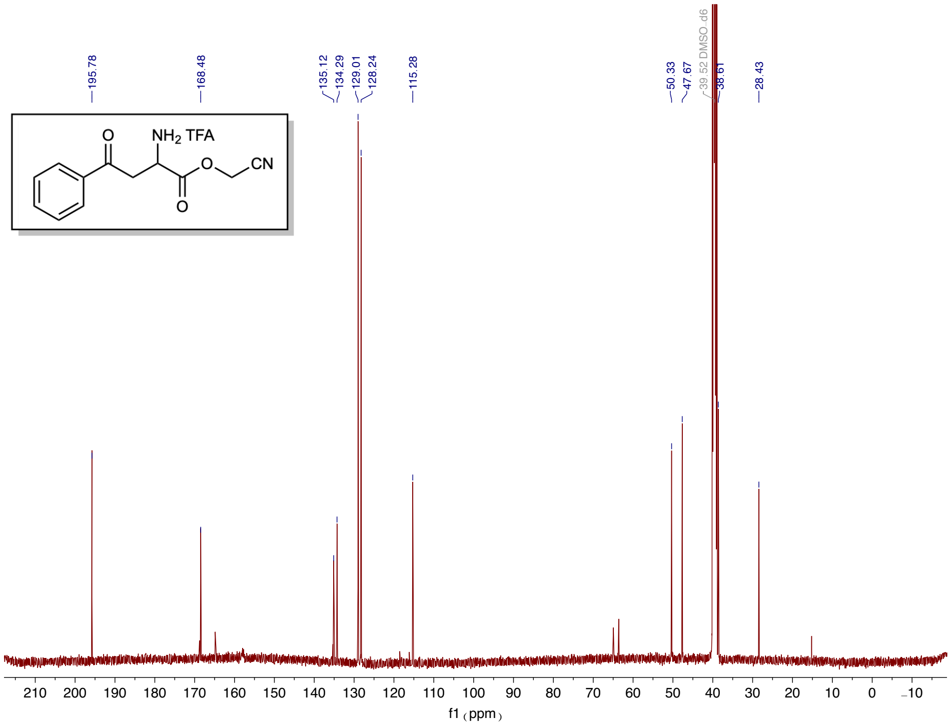

Supplementary Figure 35. ^{13}C NMR (125 MHz, DMSO-d6) of **7**.

High Resolution Mass Spectrometry

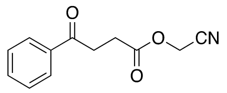

Chemical Formula: $C_{12}H_{11}NO_3$
Exact Mass: 217.0739

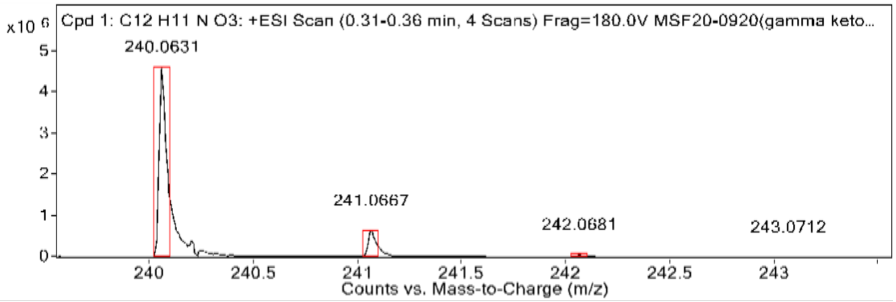

MS Spectrum Peak List

Obs. m/z	Calc. m/z	Charge	Abundance	Formula	Ion Species	Tgt Mass Error (ppm)
240.0631	240.0631	1	4591683	C12H11NO3	(M+Na) +	0.18
241.0667	241.0663	1	652943	C12H11NO3	(M+Na) +	-1.32
242.0681	242.0686	1	66064	C12H11NO3	(M+Na) +	2.06
243.0712	243.0712	1	5727	C12H11NO3	(M+Na) +	-0.12

Supplementary Figure 36. HRMS of Cyanomethyl 4-oxo-4-phenylbutanoate (**1**).

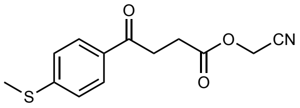

Chemical Formula: $C_{13}H_{13}NO_3S$
Exact Mass: 263.0616

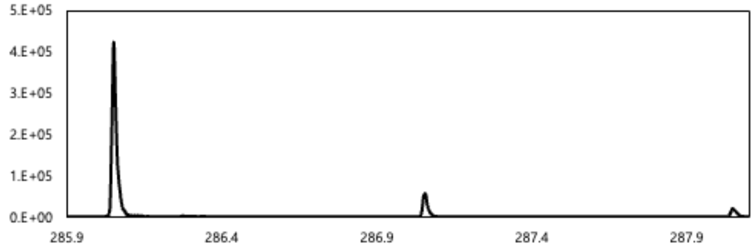

calc. for $C_{13}H_{13}NO_3S$ [M + Na]+ = 286.0513; Found 286.0511

Supplementary Figure 37. HRMS of γKPheSMe-CME (**2**).

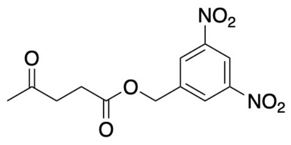

Chemical Formula: $C_{12}H_{12}N_2O_7$
Exact Mass: 296.0645

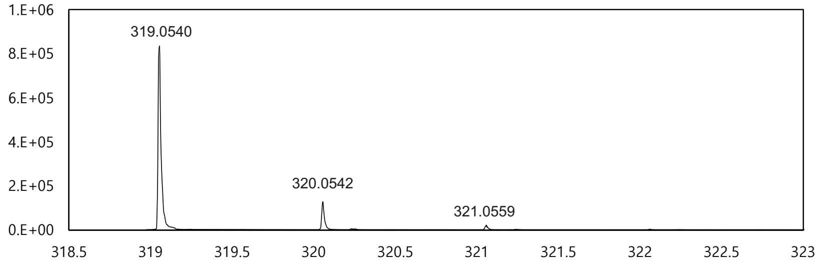

calc. for $C_{12}H_{12}N_2O_7$ [M + Na]+ = 319.0543; Found 319.0540

Supplementary Figure 38. HRMS of γKMe-DNBE (**3a**).

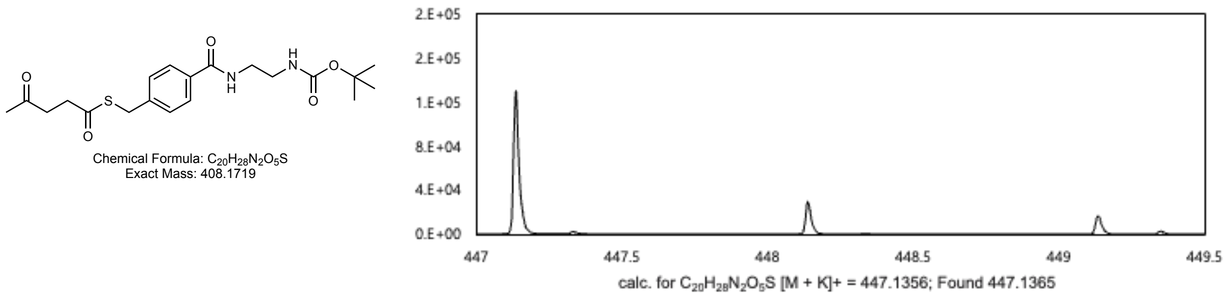

Supplementary Figure 39. HRMS of γKMe-ABT-Boc (**3b**).

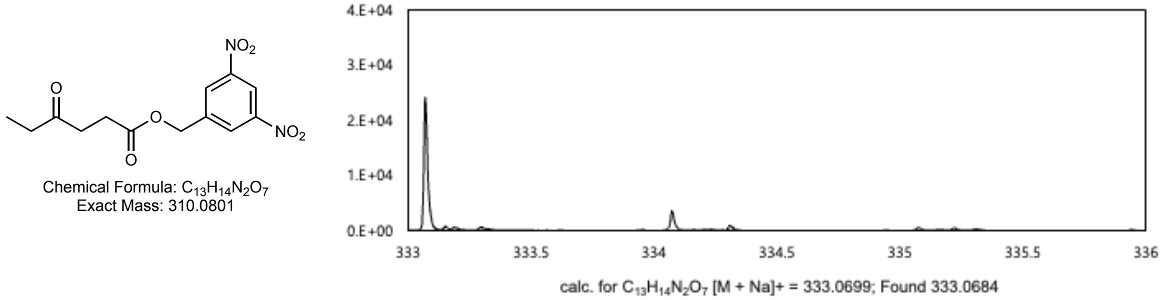

Supplementary Figure 40. HRMS of γKEt-ABT-Boc (4).

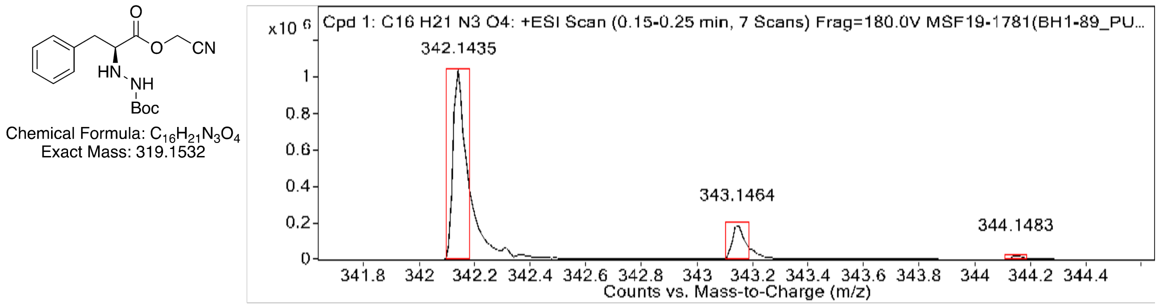

MS Spectrum Peak List

Obs. m/z	Calc. m/z	Charge	Abundance	Formula	Ion Species	Tgt Mass Error (ppm)
342.1435	342.1424	1	1044488	C16H21N3O4	(M+Na) +	-3.28
343.1464	343.1455	1	191286	C16H21N3O4	(M+Na) +	-2.66
344.1483	344.1479	1	26011	C16H21N3O4	(M+Na) +	-1.21
345.1513	345.1504	1	2303	C16H21N3O4	(M+Na) +	-2.58

Supplementary Figure 41. HRMS of Boc-HzPhe-CME (Boc-**5**).

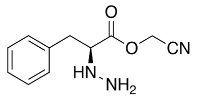

Chemical Formula: $C_{11}H_{13}N_3O_2$
Exact Mass: 219.1008

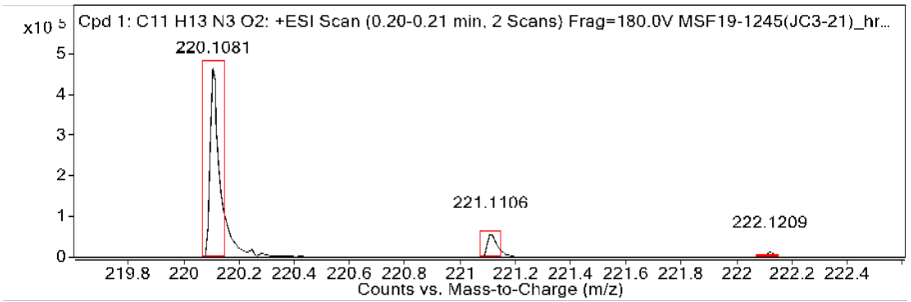

MS Spectrum Peak List

Obs. m/z	Calc. m/z	Charge	Abundance	Formula	Ion Species	Tgt Mass Error (ppm)
135.0918			1095740			
220.1081	220.1081	1	477578	C11H13N3O2	(M+H)+	0
221.1106	221.1109	1	61373	C11H13N3O2	(M+H)+	1.34
222.1209	222.1133	1	13093	C11H13N3O2	(M+H)+	-34.19
223.0482	223.1157	1	384	C11H13N3O2	(M+H)+	302.21

Supplementary Figure 42. HRMS of HzPhe-CME (**5**).

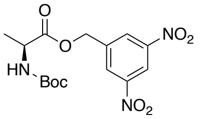

Chemical Formula: $C_{15}H_{19}N_3O_8$
Exact Mass: 369.1172

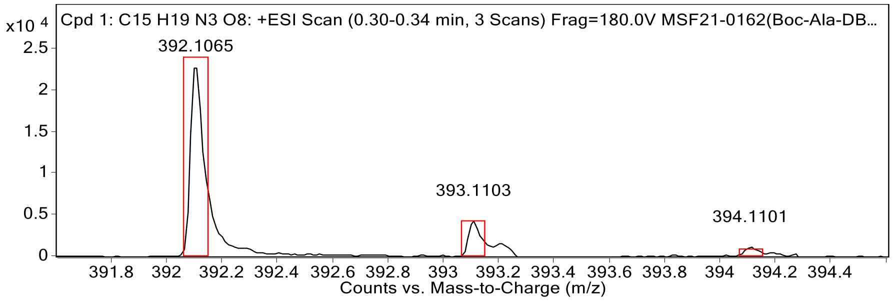

MS Spectrum Peak List

Obs. m/z	Calc. m/z	Charge	Abundance	Formula	Ion Species	Tgt Mass Error (ppm)
389.2096			23650			
392.1065	392.1064	1	23338	C15H19N3O8	(M+Na)+	-0.22
393.1103	393.1095	1	4346	C15H19N3O8	(M+Na)+	-2.13
394.1101	394.1115	1	1190	C15H19N3O8	(M+Na)+	3.62

Supplementary Figure 43. HRMS of Boc-Ala-DNB.

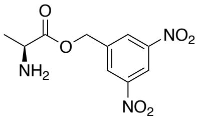

Chemical Formula: $C_{10}H_{11}N_3O_6$
Exact Mass: 269.0648

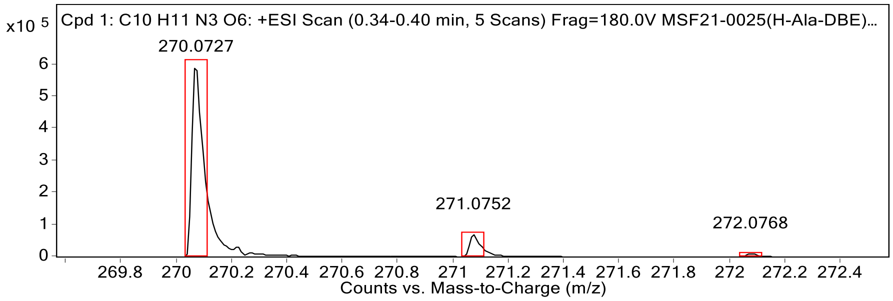

MS Spectrum Peak List

Obs. m/z	Calc. m/z	Charge	Abundance	Formula	Ion Species	Tgt Mass Error (ppm)
270.0727	270.0721	1	611509	C10H11N3O6	(M+H)+	-2.36
271.0752	271.0749	1	71404	C10H11N3O6	(M+H)+	-0.92
272.0768	272.0768	1	11673	C10H11N3O6	(M+H)+	0.07
273.0052	273.0793	1	595	C10H11N3O6	(M+H)+	271.37

Supplementary Figure 44. HRMS of Ala-DNB.

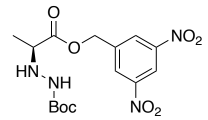

Chemical Formula: $C_{15}H_{20}N_4O_8$
Exact Mass: 384.1281

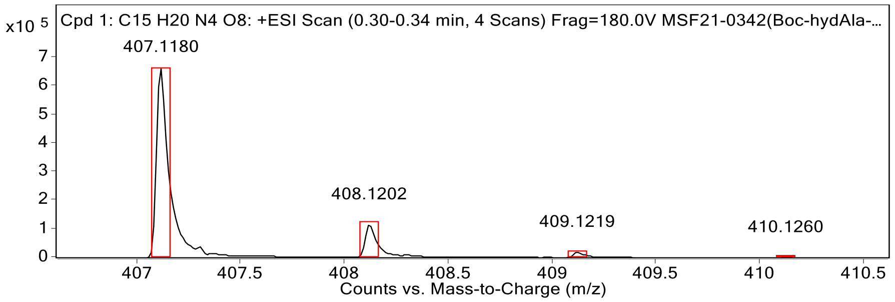

MS Spectrum Peak List

Obs. m/z	Calc. m/z	Charge	Abundance	Formula	Ion Species	Tgt Mass Error (ppm)
407.1180	407.1173	1	661935	C15H20N4O8	(M+Na)+	-1.65
408.1202	408.1202	1	117138	C15H20N4O8	(M+Na)+	0.1
409.1219	409.1223	1	20735	C15H20N4O8	(M+Na)+	1.07
410.1260	410.1248	1	2287	C15H20N4O8	(M+Na)+	-3.03
411.1052	411.1270	1	2719	C15H20N4O8	(M+Na)+	52.97
412.0974	412.1293	1	1137	C15H20N4O8	(M+Na)+	77.4

Supplementary Figure 45. HRMS of Boc-HzAla-DNB (Boc-**6a**).

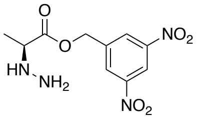

Chemical Formula: $C_{10}H_{12}N_4O_6$
Exact Mass: 284.0757

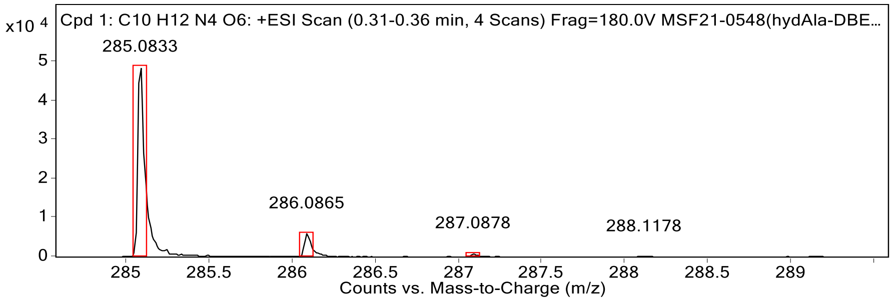

MS Spectrum Peak List

Obs. m/z	Calc. m/z	Charge	Abundance	Formula	Ion Species	Tgt Mass Error (ppm)
285.0833	285.0830	1	48802	C10H12N4O6	(M+H)+	-1.33
286.0865	286.0856	1	6123	C10H12N4O6	(M+H)+	-3
287.0878	287.0876	1	1008	C10H12N4O6	(M+H)+	-0.7
288.1178	288.0900	1	223	C10H12N4O6	(M+H)+	-96.44

Supplementary Figure 46. HRMS of Ala-DNB (**6a**).

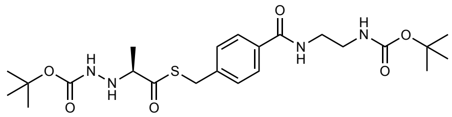

Chemical Formula: $C_{23}H_{36}N_4O_6S$
Exact Mass: 496.2356

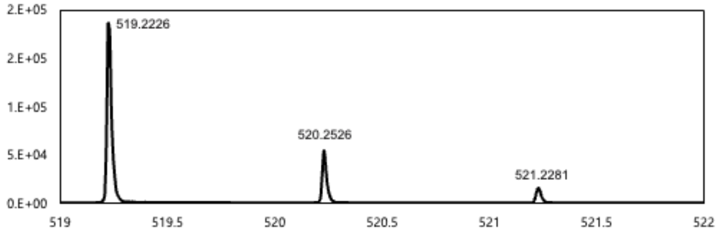

calc. for $C_{23}H_{36}N_4O_6S$ [M + Na]+ = 519.2253; Found 519.2226

Supplementary Figure 47. HRMS of Boc-HzAla-ABT-Boc (**6b**).

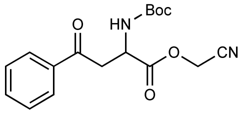

Chemical Formula: $C_{17}H_{20}N_2O_5$
Exact Mass: 332.14

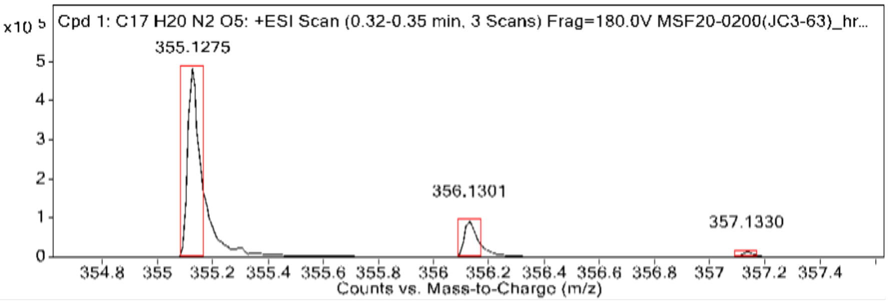

MS Spectrum Peak List

Obs. m/z	Calc. m/z	Charge	Abundance	Formula	Ion Species	Tgt Mass Error (ppm)
355.1275	355.1264	1	487170	C17H20N2O5	(M+Na)+	-2.89
356.1301	356.1296	1	92992	C17H20N2O5	(M+Na)+	-1.44
357.1330	357.1320	1	13370	C17H20N2O5	(M+Na)+	-2.67
358.1330	358.1346	1	1749	C17H20N2O5	(M+Na)+	4.29
359.2038	359.1370	1	899	C17H20N2O5	(M+Na)+	-185.91

Supplementary Figure 48. HRMS of Boc-AOP-CME (Boc-**7**).

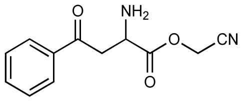

Chemical Formula: $C_{12}H_{12}N_2O_3$
Exact Mass: 232.08

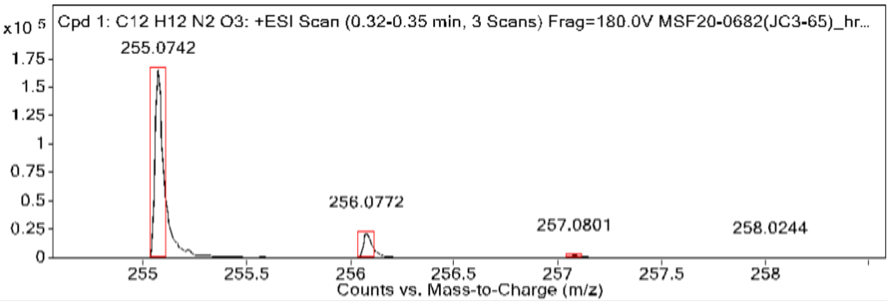

MS Spectrum Peak List

Obs. m/z	Calc. m/z	Charge	Abundance	Formula	Ion Species	Tgt Mass Error (ppm)
255.0742	255.0740	1	166586	C12H12N2O3	(M+Na) +	-0.54
256.0772	256.0771	1	22968	C12H12N2O3	(M+Na) +	-0.35
257.0801	257.0794	1	2897	C12H12N2O3	(M+Na) +	-3.06
258.0244	258.0818	1	240	C12H12N2O3	(M+Na) +	222.7
355.1268			1714403			

Supplementary Figure 49. HRMS of AOP-CME (**7**).

Plasmid map

GGATCCTGCAGTTGAGATCCTTTTTTTCTGCGCGTAATCTGCTGCTTGCAAACAAAAAAACCACCGCTACCAGC
GGTGGTTTGTTTGCCGGATCAAGAGCTACCAACTCTTTTTCCGAAGGTAACTGGCTTCAGCAGAGCGCAGATAC
CAAATACTGTCCTTCTAGTGTAGCCGTAGTTAGGCCACCACTTCAAGAACTCTGTAGCACCGCCTACATACCTC
GCTCTGCTAATCCTGTTACCAGTGGCTGCTGCCAGTGGCGATAAGTCGTGTCTTACCGGGTTGGACTCAAGACG
ATAGTTACCGGATAAGGCGCAGCGGTCGGGCTGAACGGGGGGTTCGTGCACACAGCCCAGCTTGGAGCGAACGA
CCTACACCGAACTGAGATACCTACAGCGTGAGCATTGAGAAAGCGCCACGCTTCCCGAAGGGAGAAAGGCGGAC
AGGTATCCGGTAAGCGGCAGGGTCGGAACAGGAGAGCGCACGAGGGAGCTTCCAGGGGGAAACGCCTGGTATCT
TTATAGTCCTGTCGGGTTTCGCCACCTCTGACTTGAGCGTCGATTTTTGTGATGCTCGTCAGGGGGGCGGAGCC
TATGGAAACGAATTCAGATCTCGATCCCGCGAAATTAATACGACTCACTATAGGGAGACCACAACGGTTTCCCT
CTAGAAATAATTTTGTTTAACTTTAAGAAGGAGATATA[***CATATG*****TGGTCTCATCCGCAGTTCGAAAAATAGTA**
A***GTCGAC*****]**CGGCTGCTAACAAAGCCCGAAAGGAAGCTGAGTTGGCTGCTGCCACCGCTGAGCAATAACTAGCAT
AACCCCTTGGGGCCTCTAAACGGGTCTTGAGGGGTTTTTTGCTGAAAGCCAATTCTGATTAGAAAAACTCATCG
AGCATCAAATGAAACTGCAATTTATTCATATCAGGATTATCAATACCATATTTTTGAAAAAGCCGTTTCTGTAA
TGAAGGAGAAAACTCACCGAGGCAGTTCCATAGGATGGCAAGATCCTGGTATCGGTCTGCGATTCCGACTCGTC
CAACATCAATACAACCTATTAATTTCCCCTCGTCAAAAATAAGGTTATCAAGTGAGAAATCACCATGAGTGACG
ACTGAATCCGGTGAGAATGGCAAAAGCTTATGCATTTCTTTCCAGACTTGTTCAACAGGCCAGCCATTACGCTC
GTCATCAAAATCACTCGCATCAACCAAACCGTTATTCATTCGTGATTGCGCCTGAGCGAGACGAAATACGCGAT
CGCTGTTAAAAGGACAATTACAAACAGGAATCGAATGCAACCGGCGCAGGAACACTGCCAGCGCATCAACAATA
TTTTCACCTGAATCAGGATATTCTTCTAATACCTGGAATGCTGTTTTCCCGGGGATCGCAGTGGTGAGTAACCA
TGCATCATCAGGAGTACGGATAAAATGCTTGATGGTCGGAAGAGGCATAAATTCCGTCAGCCAGTTTAGTCTGA
CCATCTCATCTGTAACATCATTGGCAACGCTACCTTTGCCATGTTTCAGAAACAACTCTGGCGCATCGGGCTTC
CCATACAATCGATAGATTGTCGCACCTGATTGCCCGACATTATCGCGAGCCCATTTATACCCATATAAATCAGC
ATCCATGTTGGAATTTAATCGCGGCTTCGAGCAAGACGTTTCCCGTTGAATATGGCTCATAACACCCCTTGTAT
TACTGTTTATGTAAGCAGACAGTTTTATTGTTCATGATGATATATTTTTATCTTGTGCAATGTAACATCAGAGA
TTTTGAGACACAACGT

***CATATG*: NdeI**
***GTCGAC:* SalI**
TGGTCTCATCCGCAGTTCGAAAAA: strep tag
TAGTAA: stop

>pJL1_MT_StrepII
[*CATATGTCC*TGGTCTCATCCGCAGTTCGAAAAATAGTAA*GTCGAC*]
fMetThrTrpSerHisProGlnPheGluLys

>pJL1_StrepII_TI2
[*CATATG*TGGTCTCATCCGCAGTTCGAAAAATCC*ACCATCACCATC*TAGTAA*GTCGAC*]
fMetTrpSerHisProGlnPheGluLysSerThrIleThrIle

>pJL1_StrepII_TI3
[*CATATG*TGGTCTCATCCGCAGTTCGAAAAATCC*ACCATCACCATCACCATC*TAGTAA*GTCGAC*]
fMetTrpSerHisProGlnPheGluLysSerThrIleThrIleThrIle

List of primers

Fx-T7F	GGCGTAATACGACTCACTATAG
Fx_F	GTAATACGACTCACTATAGGATCGAAAGATTTCCGC
eFX-R1	ACCTAACGCTAATCCCCTTTCGGGGCCGCGGAAATCTTTCGATCC
dFX-R1	ACCTAACGCCATGTACCCTTTCGGGGATGCGGAAATCTTTCGATCC
aFX-R1	ACCTAACGCCACTTACCCCTTTCGGGGGTGCGGAAATCTTTCGATCC
eFx-R2	ACCTAACGCTAATCCCCT
dFx-R2	ACCTAACGCCATGTACCCT
aFx-R2	ACCTAACGCCACTTACCCC
fMetE-F	GTAATACGACTCACTATAGGCGGGGTGGAGCAGCCTGGTAGCTCGTCGG
fMetE-R1	GAACCGACGATCTTCGGGTTATGAGCCCGACGAGCTACCAGGCT
fMetE-R2	TGGTTGCGGGGGCCGGATTTGAACCGACGATCTTCGGG
fMetE-R3	TGGTTGCGGGGGCCGGATTT
Pro1E2-F	GTAATACGACTCACTATAGGGTGATTGGCGCAGCCTGGTAGCGCACTTC
Pro1E2-R1	GAACCCCTGACCCCTTCGTTACCAACGAAGTGCGCTACCAGGCT
Pro1E2-R2	TGGCGGGTGATAGGGGATTCGAACCCCTGACCCCTTCG
Pro1E2-R3	TGGCGGGTGATAGGGGATTC

References

1. Niwa, N., Yamagishi, Y., Murakami, H. & Suga, H. A flexizyme that selectively charges amino acids activated by a water-friendly leaving group. *Bioorg Med Chem Lett* **19**, 3892-3894 (2009).
2. Kang, C.W., Sarnowski, M.P., Elbatrawi, Y.M. & Del Valle, J.R. Access to Enantiopure alpha-Hydrazino Acids for N-Amino Peptide Synthesis. *J Org Chem* **82**, 1833-1841 (2017).
3. Armstrong, A., Jones, L.H., Knight, J.D. & Kelsey, R.D. Oxaziridine-mediated amination of primary amines: scope and application to a one-pot pyrazole synthesis. *Org Lett* **7**, 713-716 (2005).
4. Lee, J. et al. Expanding the limits of the second genetic code with ribozymes. *Nat Commun* **10**, 5097 (2019).
5. Maini, R. et al. Protein Synthesis with Ribosomes Selected for the Incorporation of beta-Amino Acids. *Biochemistry* **54**, 3694-3706 (2015).
6. Katoh, T., Wohlgemuth, I., Nagano, M., Rodnina, M.V. & Suga, H. Essential structural elements in tRNA(Pro) for EF-P-mediated alleviation of translation stalling. *Nat Commun* **7**, 11657 (2016).
7. Tuckey, C., Asahara, H., Zhou, Y. & Chong, S. Protein synthesis using a reconstituted cell-free system. *Curr Protoc Mol Biol* **108**, 16 31 11-22 (2014).
