# Supplementary material for: Ribosome-mediated biosynthesis of pyridazinone oligomers in vitro
Source: Nat Commun. 2022 Oct 24;13:6322. doi: 10.1038/s41467-022-33701-2 (PMC9592601; doi:10.1038/s41467-022-33701-2)
Supplement: Supplementary file 2 — Reporting Summary [file 41467_2022_33701_MOESM2_ESM.pdf]

nature portfolio

Corresponding author(s): Michael Jewett

Last updated by author(s): Sep 24, 2022

Reporting Summary

Nature Portfolio wishes to improve the reproducibility of the work that we publish. This form provides structure for consistency and transparency in reporting. For further information on Nature Portfolio policies, see our Editorial Policies and the Editorial Policy Checklist.

Statistics

For all statistical analyses, confirm that the following items are present in the figure legend, table legend, main text, or Methods section.

n/a	Confirmed	
[ ]	[x]	The exact sample size (n) for each experimental group/condition, given as a discrete number and unit of measurement
[ ]	[x]	A statement on whether measurements were taken from distinct samples or whether the same sample was measured repeatedly
[x]	[ ]	The statistical test(s) used AND whether they are one- or two-sided<br>*Only common tests should be described solely by name; describe more complex techniques in the Methods section.*
[x]	[ ]	A description of all covariates tested
[x]	[ ]	A description of any assumptions or corrections, such as tests of normality and adjustment for multiple comparisons
[ ]	[x]	A full description of the statistical parameters including central tendency (e.g. means) or other basic estimates (e.g. regression coefficient) AND variation (e.g. standard deviation) or associated estimates of uncertainty (e.g. confidence intervals)
[x]	[ ]	For null hypothesis testing, the test statistic (e.g. F, t, r) with confidence intervals, effect sizes, degrees of freedom and P value noted<br>*Give P values as exact values whenever suitable.*
[x]	[ ]	For Bayesian analysis, information on the choice of priors and Markov chain Monte Carlo settings
[x]	[ ]	For hierarchical and complex designs, identification of the appropriate level for tests and full reporting of outcomes
[x]	[ ]	Estimates of effect sizes (e.g. Cohen's d, Pearson's r), indicating how they were calculated

Our web collection on statistics for biologists contains articles on many of the points above.

Software and code

Policy information about availability of computer code

Data collection	All data were collected using stated instruments and associated commercially available software. The nuclear magnetic resonance spectra (NMR) were processed using TopSpin v4.0.5 (Bruker) or ACD v12.01 (ACD/Labs). ESI mass spectra were obtained using HyStar v4.1 (Bruker) or MassLynx 4.1 (Waters). MALDI mass spectra was obtained using FlexControl v2.0 (Bruker). The yields of microhelix acylation reaction were determined by densitometrically using ImageJ software. No in-house algorithms or softwares were used to collect the data.
Data analysis	All data were analyzed using stated instruments and associated commercially-available softwares. Mass spectra were recorded on a Bruker Rapiflex, Bruker Autoflex, AmaZon SL, or Waters Q-TOF Ultima for electron-spray ionization (ESI) and Impact-II or Waters 70-VSE for electron impact (EI). High resolution mass spectrometry (HRMS) analysis was performed by an Agilent 6530 Accurate Mass Q-TOF LC/MS system. 1H and 13C NMR spectra were collected either using the Bruker AVANCE III HD 500 MHz cryoprobe NMR spectrometer and processed by TopSpin or MestReNova. No custom algorithms or softwares were used for analysis.

For manuscripts utilizing custom algorithms or software that are central to the research but not yet described in published literature, software must be made available to editors and reviewers. We strongly encourage code deposition in a community repository (e.g. GitHub). See the Nature Portfolio guidelines for submitting code & software for further information.

Data

Policy information about availability of data

All manuscripts must include a data availability statement. This statement should provide the following information, where applicable:

- Accession codes, unique identifiers, or web links for publicly available datasets
- A description of any restrictions on data availability
- For clinical datasets or third party data, please ensure that the statement adheres to our policy

All data generated or analyzed in this study are included in this published article (and its supplementary files) or are available from the corresponding authors upon reasonable request.

Field-specific reporting

Please select the one below that is the best fit for your research. If you are not sure, read the appropriate sections before making your selection.

- [x] Life sciences
- [ ] Behavioural & social sciences
- [ ] Ecological, evolutionary & environmental sciences

For a reference copy of the document with all sections, see nature.com/documents/nr-reporting-summary-flat.pdf

Life sciences study design

All studies must disclose on these points even when the disclosure is negative.

Sample size	Quantification of flexizyme-mediated tRNA acylation yield (%) in the supplementary materials were obtained from the mean of multiple (n=1-3) independent reactions. Biological triplicates of each sample were used to verify reproducibility. We provide the relevant citations using the same sample sizes. (References: Nat. Commun. 2020, 11, 4304, Nat. Commun. 2019, 10, 5097, Chem. Commun. 2020, 56, 5597, Chem. Commun. 2021, 57, 2661.) All pyridazinone oligomer synthesis data were also acquired from multiple (n=1-3) independent in vitro protein express reactions. The protein expression was performed using a commercially available in vitro protein expression package (PURExpress, NEB) and the kit was validated by the vendor. The produced peptides were identified by MALDI analysis using multiple (n>3) samples. The pyridazinone oligomers produced from multiple experiments were collected and analyzed at the same time at once.<br>This is standard practice for characterization of acylation yield and peptides produced in cell-free protein synthesis system. The mass spectroscopic analysis allowed us to confirm the site-specifically introduced pyridazinones in a peptide, indicating that the sample size did not impact quantification.
Data exclusions	No data were excluded.
Replication	All attempts for flexizyme mediated tRNA charging and in vitro pyridazinone synthesis at replication were successful. Triplicates of each sample were used to verify reproducibility.
Randomization	All pyridazinone oligomers containing non-canonical chemical substrate were analyzed by mass spectrometry. Samples were analyzed equally as obtained, then fully characterized. There was no requirement for randomization.
Blinding	Blinding was not relevant because animal or human participants were not used in this study.

Reporting for specific materials, systems and methods

We require information from authors about some types of materials, experimental systems and methods used in many studies. Here, indicate whether each material, system or method listed is relevant to your study. If you are not sure if a list item applies to your research, read the appropriate section before selecting a response.

Materials & experimental systems

n/a	Involved in the study	
[ ]	[x]	Antibodies
[x]	[ ]	Eukaryotic cell lines
[x]	[ ]	Palaeontology and archaeology
[x]	[ ]	Animals and other organisms
[x]	[ ]	Human research participants
[x]	[ ]	Clinical data
[x]	[ ]	Dual use research of concern

Methods

n/a	Involved in the study	
[x]	[ ]	ChIP-seq
[x]	[ ]	Flow cytometry
[x]	[ ]	MRI-based neuroimaging

Antibodies

Antibodies used	MagStrep type3 XT Beads 5% suspension (iba GmbH)

Validation	We used commercially available Strep-Tactin protein coated ferri-magnetic beads for purification of produced peptides containing a StrepII tag (WSHPQFEK) and a non-canonical chemical substrate. This product was validated by the vendor. No secondary antibodies were used.
