# Supplementary material for: Ribosome-mediated biosynthesis of pyridazinone oligomers in vitro
Source: Nat Commun. 2022 Oct 24;13:6322. doi: 10.1038/s41467-022-33701-2 (PMC9592601; doi:10.1038/s41467-022-33701-2)
Supplement: Supplementary file 3 — Source Data [file 41467_2022_33701_MOESM3_ESM.zip › gels.pptx]

## Slide 1
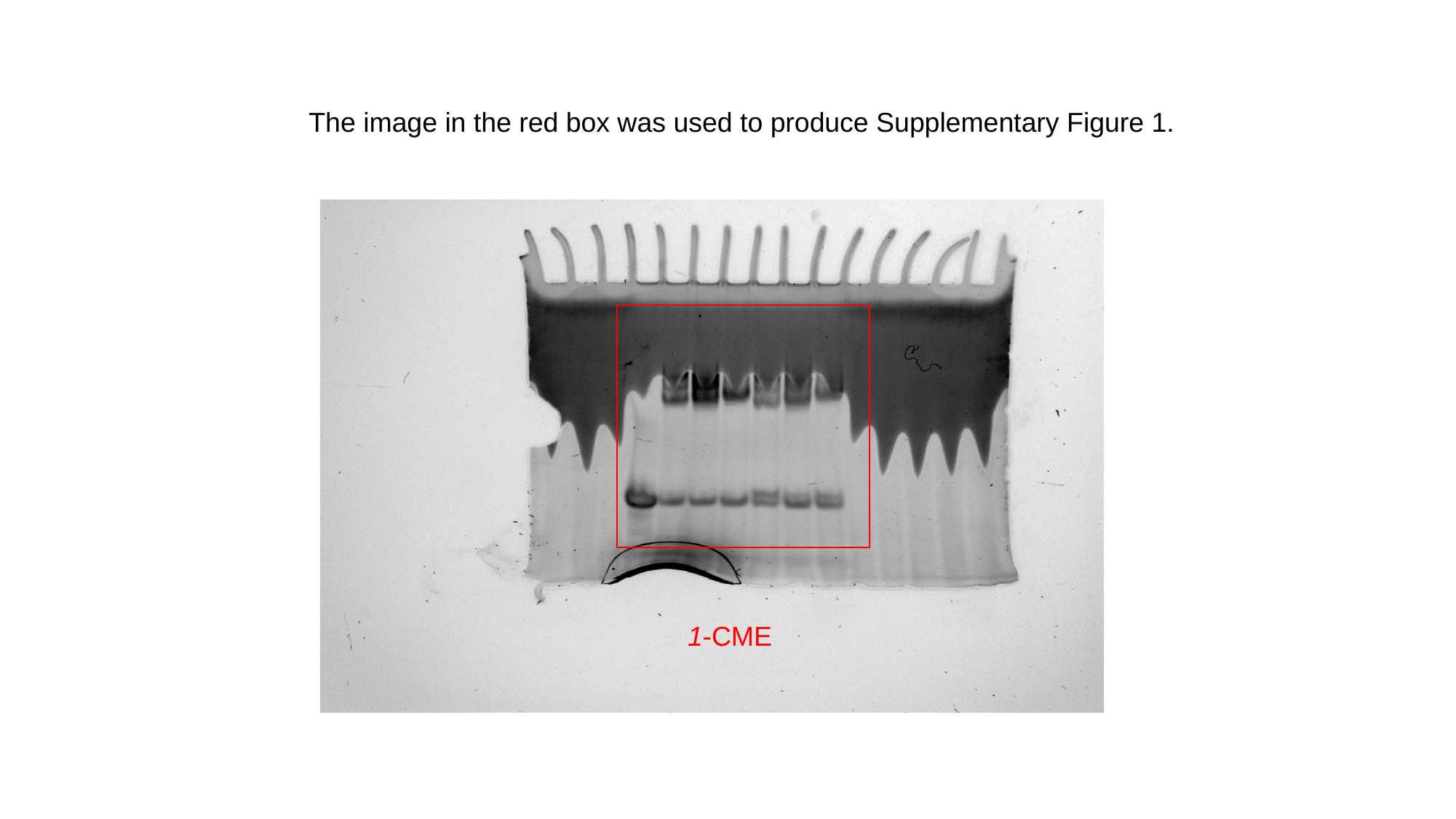

The image in the red box was used to produce Supplementary Figure 1.
1-CME

## Slide 2
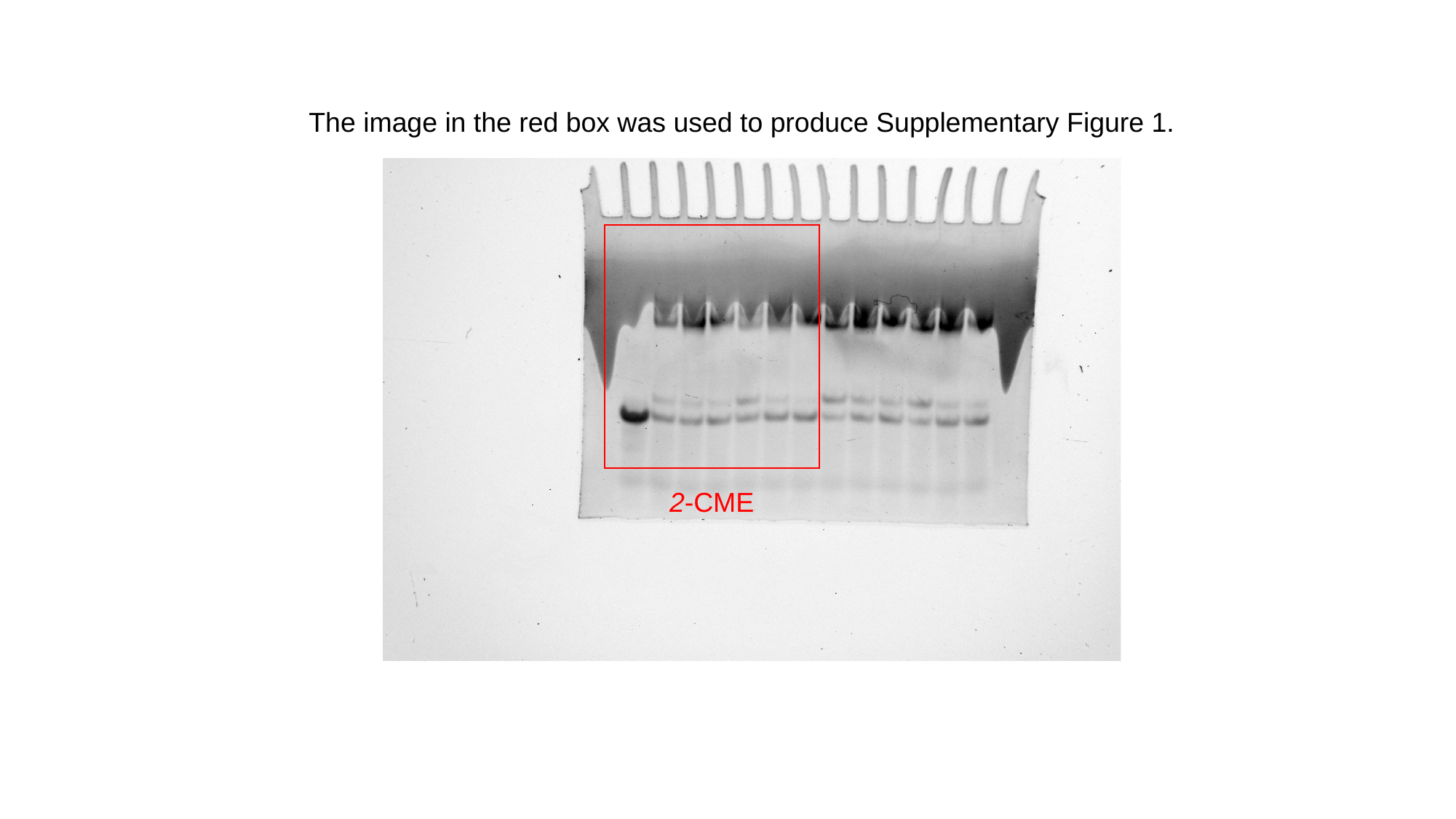

The image in the red box was used to produce Supplementary Figure 1.
2-CME

## Slide 3
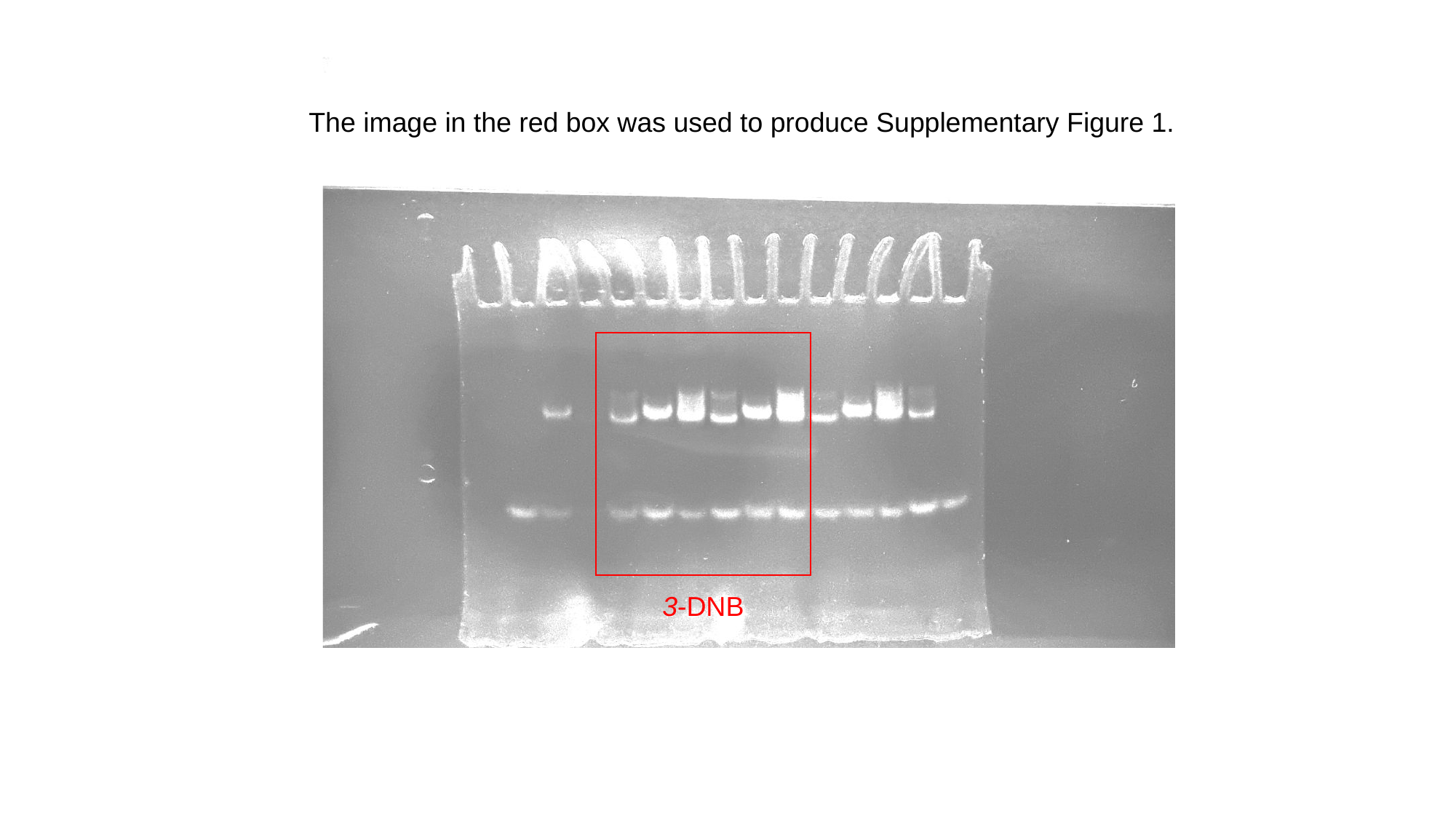

The image in the red box was used to produce Supplementary Figure 1.
3-DNB

## Slide 4
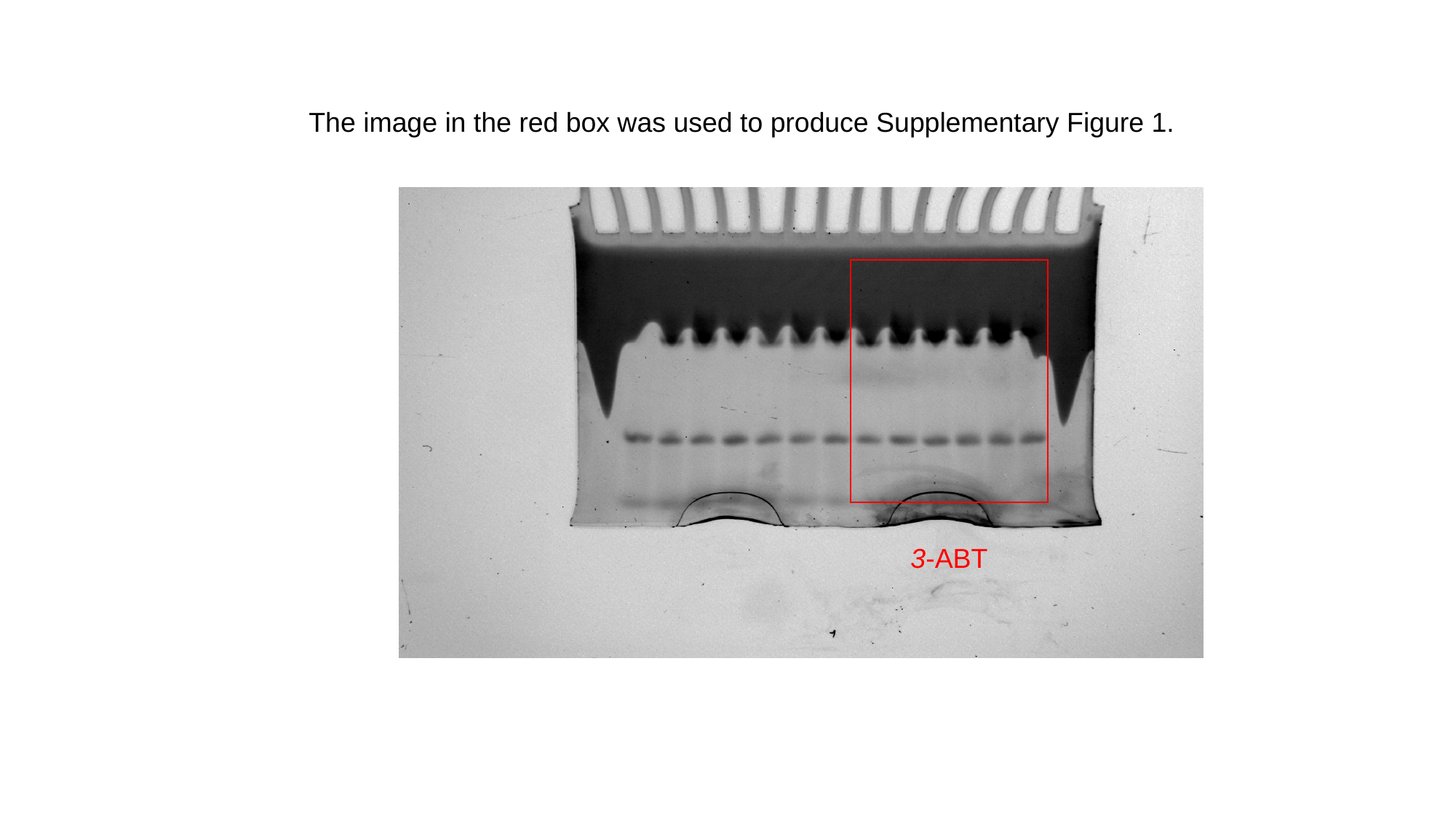

The image in the red box was used to produce Supplementary Figure 1.
3-ABT

## Slide 5
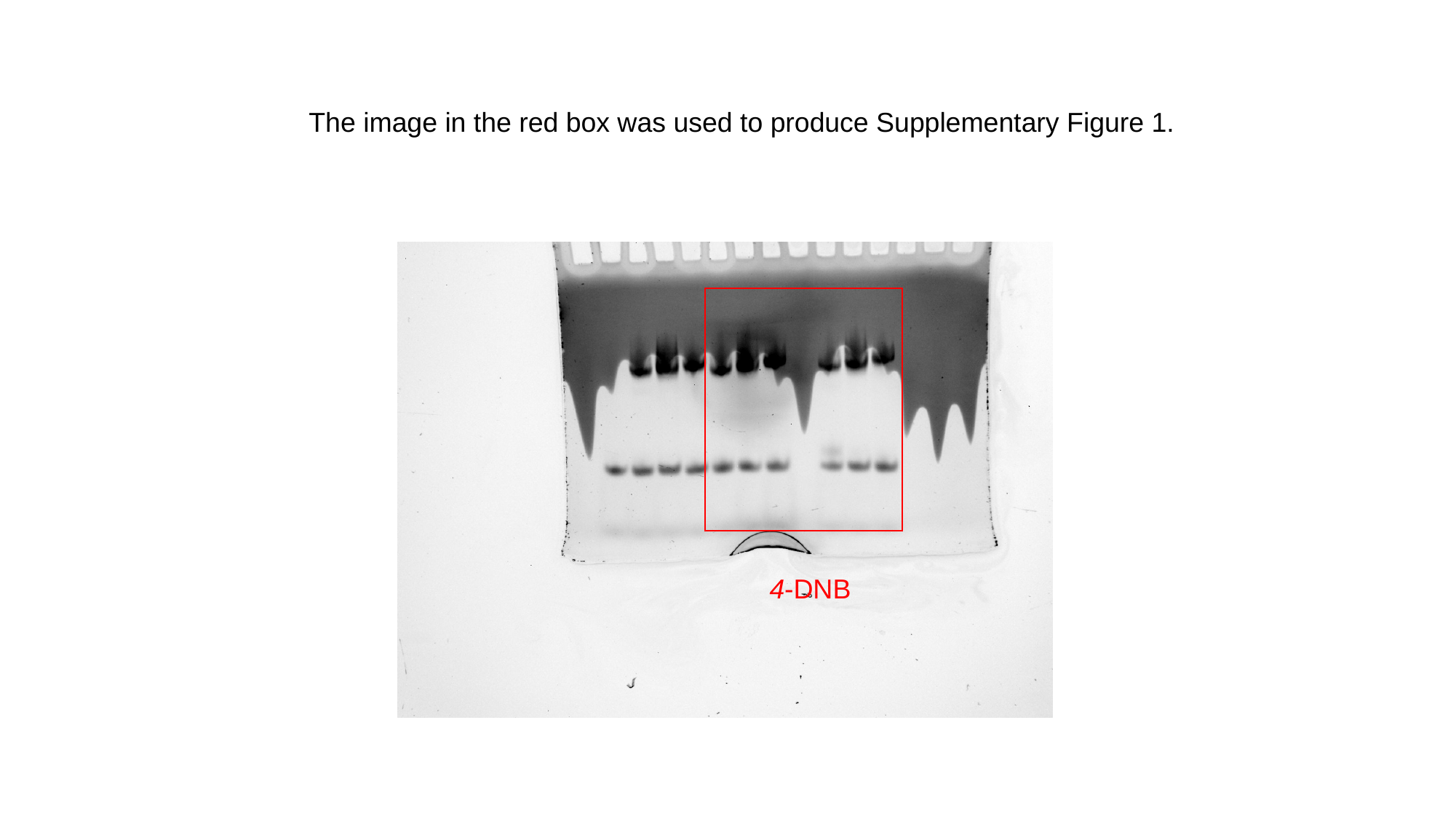

The image in the red box was used to produce Supplementary Figure 1.
4-DNB

## Slide 6
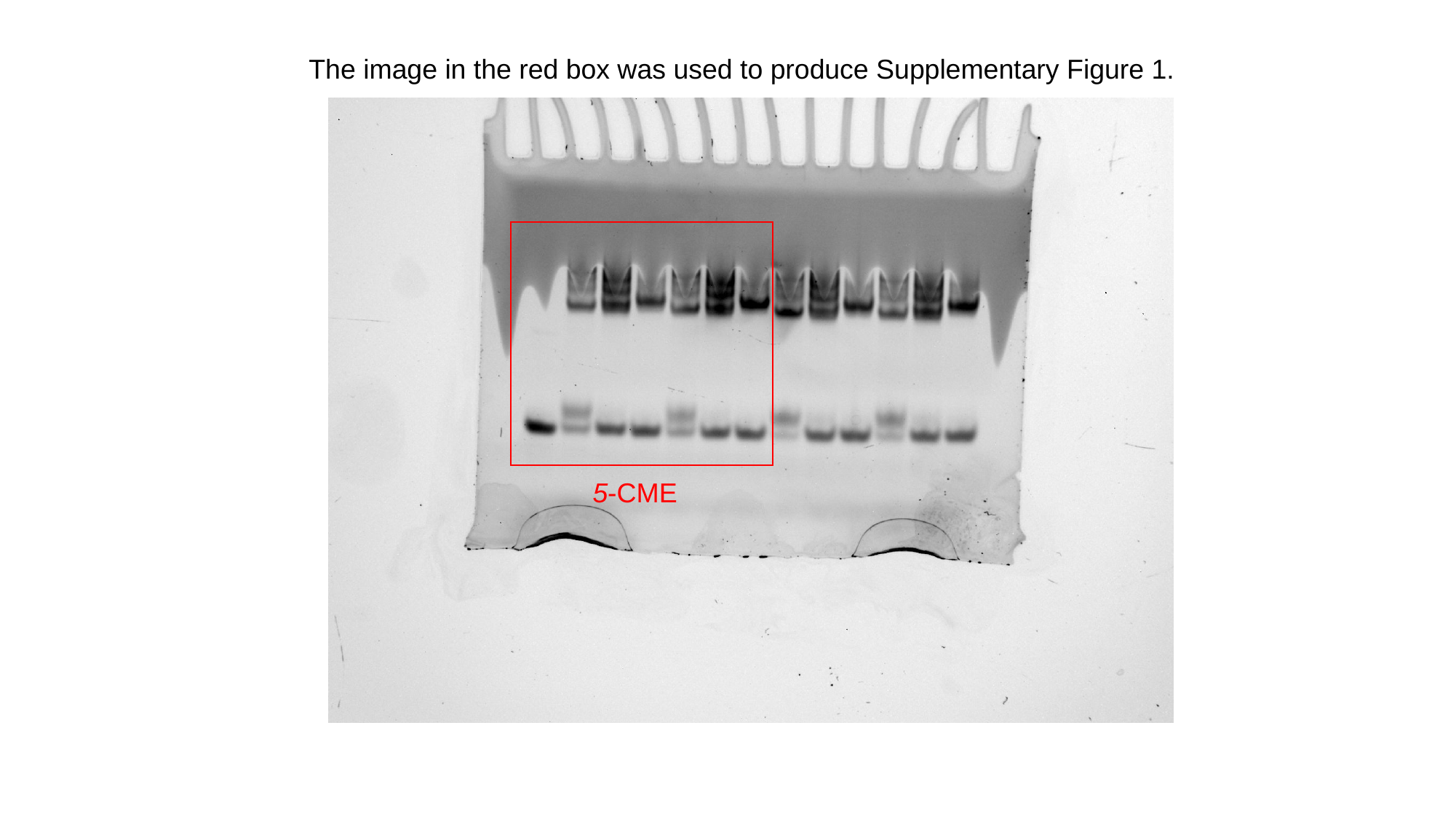

The image in the red box was used to produce Supplementary Figure 1.
5-CME

## Slide 7
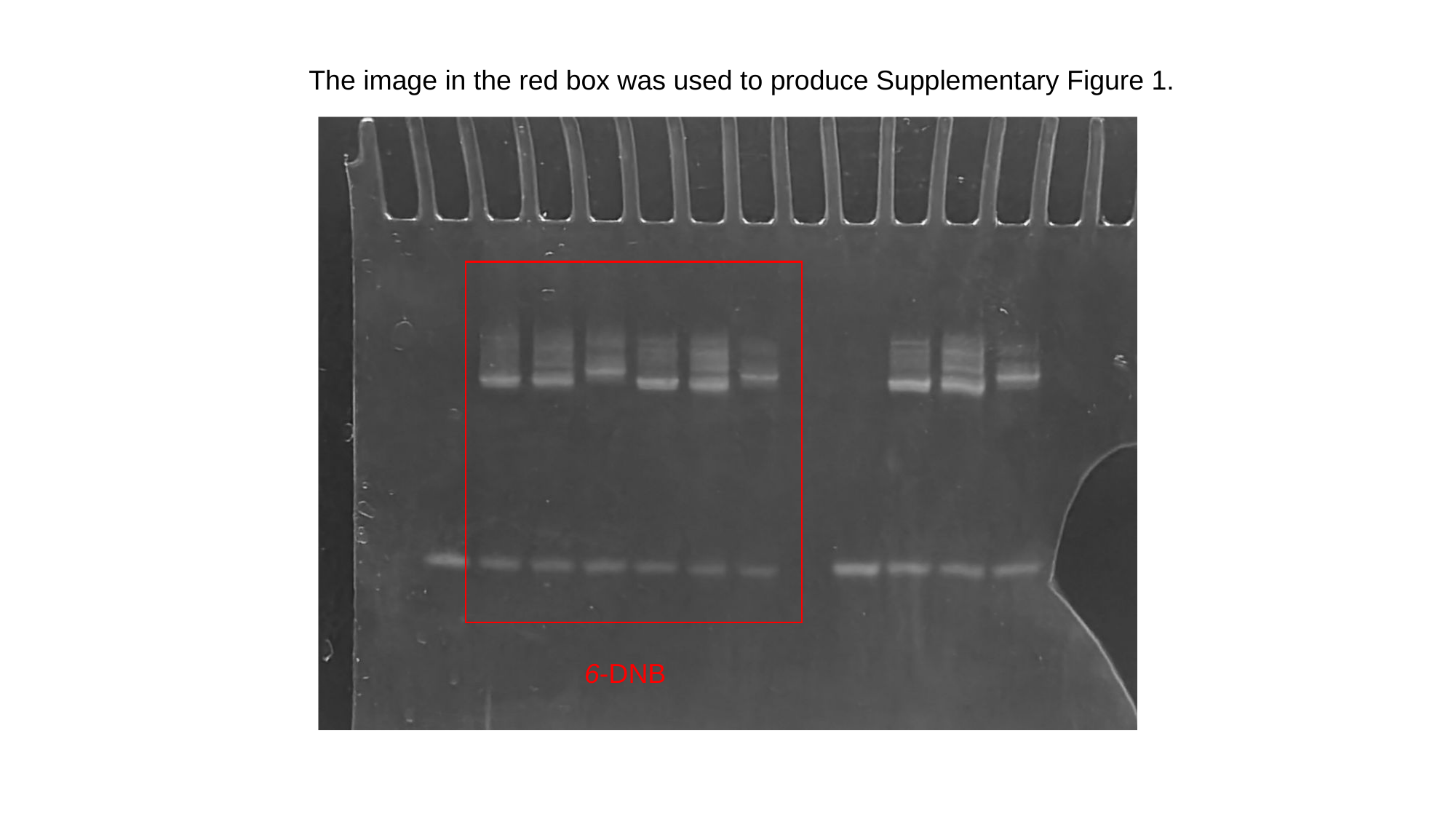

The image in the red box was used to produce Supplementary Figure 1.
6-DNB

## Slide 8
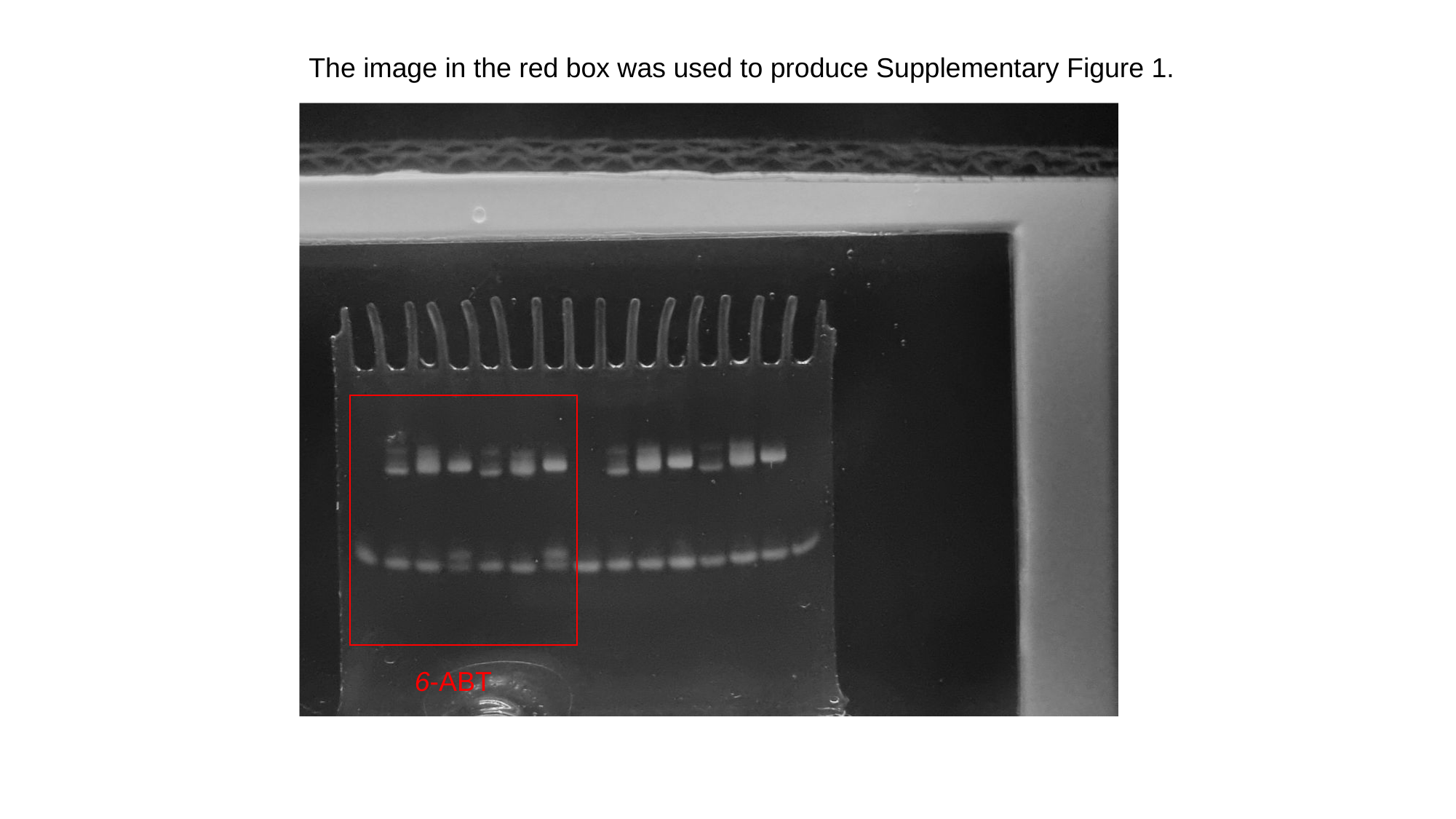

The image in the red box was used to produce Supplementary Figure 1.
6-ABT

## Slide 9
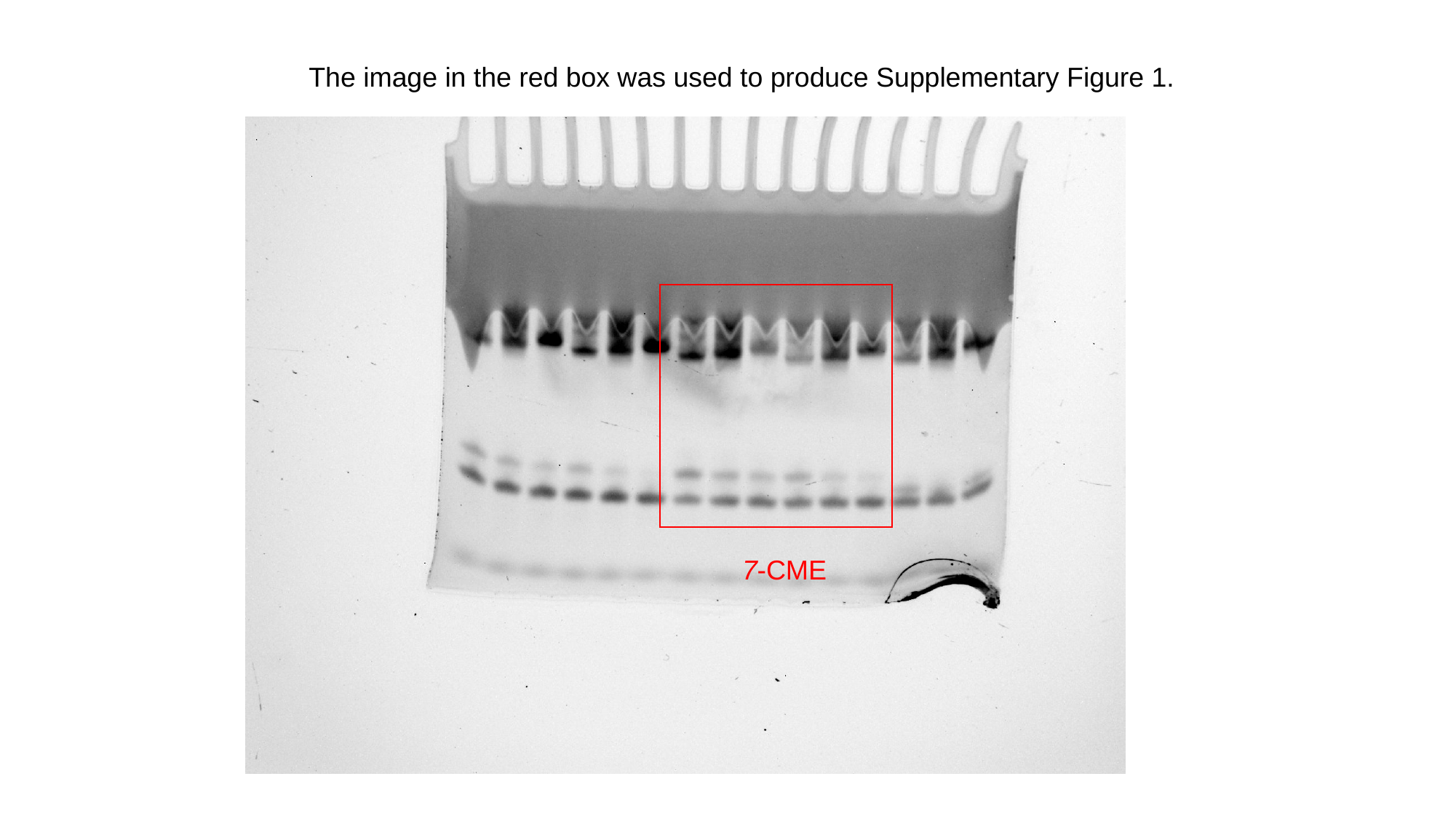

The image in the red box was used to produce Supplementary Figure 1.
7-CME
